# Tuning Pt^II^‐Based Donor–Acceptor Systems through Ligand Design: Effects on Frontier Orbitals, Redox Potentials, UV/Vis/NIR Absorptions, Electrochromism, and Photocatalysis

**DOI:** 10.1002/chem.201903700

**Published:** 2020-01-22

**Authors:** Sebastian Sobottka, Maite Nößler, Andrew L. Ostericher, Gunter Hermann, Noah Z. Subat, Julia Beerhues, Margarethe Behr‐van der Meer, Lisa Suntrup, Uta Albold, Stephan Hohloch, Jean Christophe Tremblay, Biprajit Sarkar

**Affiliations:** ^1^ Institut für Chemie und Biochemie Anorganische Chemie Freie Universität Berlin Fabeckstrasse 34–36 14195 Berlin Germany; ^2^ Current address: Department of Chemistry and Biochemistry University of California San Diego 9500 Gilman Drive La Jolla CA 92093 USA; ^3^ QoD Technologies GmbH c/o Freie Universität Berlin Altensteinstrasse 40 14195 Berlin Germany; ^4^ Current address: Department of Chemistry University of Massachusetts Boston 100 Morrissey Boulevard Boston MA 02125 USA; ^5^ Current address: University of Paderborn Warburger Strasse 100 33098 Paderborn Germany; ^6^ Laboratoire de physique et chimie théoriques CNRS/Université de Lorraine—UMR 7019 1 bd Arago 57070 Metz France; ^7^ Institut für Anorganische Chemie Universität Stuttgart Pfaffenwaldring 55 70569 Stuttgart Germany

**Keywords:** donor–acceptor systems, electrochemistry, platinum, redox-active ligands, spectroelectrochemistry

## Abstract

Asymmetric platinum donor–acceptor complexes [(pimp)Pt(Q^2−^)] are presented in this work, in which pimp=[(2,4,6‐trimethylphenylimino)methyl]pyridine and Q^2−^=catecholate‐type donor ligands. The properties of the complexes are evaluated as a function of the donor ligands, and correlations are drawn among electrochemical, optical, and theoretical data. Special focus has been put on the spectroelectrochemical investigation of the complexes featuring sulfonyl‐substituted phenylendiamide ligands, which show redox‐induced linkage isomerism upon oxidation. Time‐dependent density functional theory (TD‐DFT) as well as electron flux density analysis have been employed to rationalize the optical spectra of the complexes and their reactivity. Compound **1** ([(pimp)Pt(Q^2−^)] with Q^2−^=3,5‐di‐*tert*‐butylcatecholate) was shown to be an efficient photosensitizer for molecular oxygen and was subsequently employed in photochemical cross‐dehydrogenative coupling (CDC) reactions. The results thus display new avenues for donor–acceptor systems, including their role as photocatalysts for organic transformations, and the possibility to introduce redox‐induced linkage isomerism in these compounds through the use of sulfonamide substituents on the donor ligands.

## Graphical abstract

**Outcomes of ligand design**: A series of platinum‐based donor–acceptor complexes is presented. The effect of the donor ligands on the frontier orbitals, redox potentials, redox‐induced isomerism, and photocatalysis is probe.

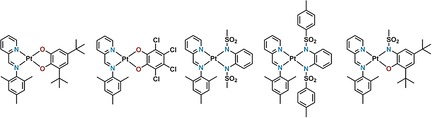

## Introduction

Group 10 metals in their d^8^ electronic configuration have served to synthesize a range of donor–acceptor metal complexes.^1^, ^2^, ^3^, ^4^, ^5^ These compounds are usually characterized by intense ligand‐to‐ligand charge transfers (LL′CTs), which impart unique photochemical and photophysical properties on the resulting metal complexes.^2^, ^6^ Applications range from dye‐sensitized solar cells to small molecule activation and catalysis.^4^, ^7^, ^8^

**Figure 1:**
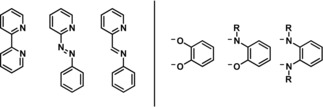
Typical acceptor (left) and donor (right) ligands.

To obtain a compound with a strong LL′CT transition, it is desirable to use a strong π‐acceptor and a strong σ‐ and/or π‐donor ligand, with favorable orbital energies. Figure 1 shows prototypical ligands that have been successfully employed in this regard. Well‐established acceptor ligands are 2,2′‐bipyridine (bpy), phenylazopyridine (pap), and also phenyliminomethylpyridine (pimp), with the latter only sparingly used in the construction of such systems.^9^, ^10^, ^11^ Another interesting application for compounds with optoelectronically switchable properties are electrochromic devices.6c, 12

As donor ligands, catecholate/semiquinone/quinone ligands have been well‐established due to their strong donor properties and well‐defined redox behavior.^7^, ^13^ A change in oxidation state for this ligand system results in drastically altered donor and acceptor properties with the catecholate ligand being a strong π‐ and σ‐donor and poor acceptor ligand, whereas the fully oxidized quinone is a strong π‐acceptor and a very weak π‐donor ligand. If the oxygen donor atom is replaced by an isolobal [N‐R] residue, the quinones can be sterically and electronically tuned rather easily.^14^ All of the depicted ligands are redox‐active and thus can act in a potentially non‐innocent manner, when coordinated to a metal center. The stabilization of additional charges or charge separation becomes important, if one wishes to harvest solar energy. Our previous work revealed that such compounds show interesting and diverse reactivity, if different donor ligands were employed.^3^, ^4^

Special emphasis is put on the redox‐induced reactivity of the systems featuring *o*‐bis(sulfonamide) ligands in this work. Although this ligand class was described for the first time more than half a century ago,^15^ the application of this highly tunable ligand class is still rather limited^16^, ^17^ and only one platinum complex has been reported.^18^ For the most part, these reports discuss fundamental structural aspects of the complexes. Recently, we reported mono‐ and dinuclear cobalt(II) complexes with chelating and bridging bis(sulfonamido)benzene ligands resulting in air‐stable single molecular magnets with high switching barriers, highlighting the potential of this ligand class.^19^

Perutz and co‐workers studied rhodium(III) complexes with symmetrically and asymmetrically sulfonylated bis(amido)benzenes for transfer hydrogenation, showing the catalytic applications of these ligands.^20^, ^21^, ^22^ Interestingly, they also observed the dimerization of the aforementioned rhodium compounds, in which the oxygen atoms of the sulfonyl group bridge two rhodium centers.^20^ Kavallieratos and co‐workers observed the formation of coordination polymers using lead(II) salts, emphasizing the versatile and dynamic coordination chemistry these ligands may engage in.^17^

## Results and Discussion

### Synthesis and structural characterization

**Scheme 1:**
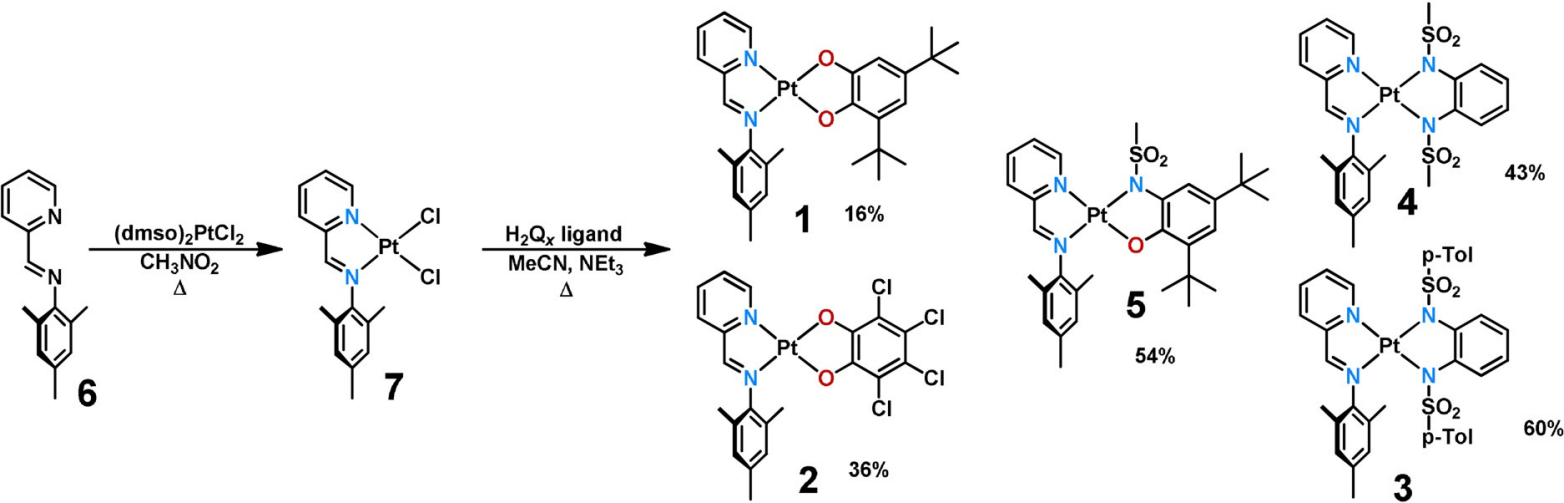
Syntheses of the precursor (pimp)PtCl_2_ (**7**) and the complexes **1**–**5** (*p*‐Tol=*para*‐tosyl).

Complexes **1**–**5** were prepared by following a previously established route (Scheme 1).^3^, ^5^ The ligands were prepared by reported reactions.^23^, ^24^ The reaction of phenyliminomethylpyridine (**6**) with (dmso)_2_PtCl_2_ yielded the platinum dichloride complex **7** in good yield. In the presence of triethylamine, the respective quinoid ligands H_2_Q_x_ were deprotonated under inert conditions in acetonitrile to give the title complexes in low to acceptable yields, as shown in Scheme 1. The compounds were initially characterized by means of ^1^H‐ and ^13^C NMR spectroscopy, mass spectrometry, and elemental analysis. All complexes are stable towards air and moisture in the solid state and in solution and can be stored for several months without detectable decomposition.

For complexes **1** and **5**, two regioisomers can be formed; however, only one isomer was isolated in contrast to previous studies.^3^ For complex **5**, even four possible isomers are conceivable, if the position of the methylsulfonyl group relative to the plane spanned by the two binding pockets and the platinum center is taken into account. However, the barrier for the rotation is probably so low that the isomers interconvert too quickly at room temperature. A systematic screening of various reaction conditions (time, temperature, solvent, and type of base) did not result in the formation of the other regioisomer or a mixture of both isomers. This is not observed for the strongly related pap (phenylazopyridine) complexes of platinum and palladium, which we have reported on earlier.^3^

This result is quite surprising; however, it may be partly explained, if the *trans*‐influence of the pyridine and imino fragment are compared. From the crystal structure of (pimp)PtCl_2_ (**7**), we can see that the platinum–chloride bond in the *trans*‐position to the imino function is slightly elongated compared with the platinum–chloride bond in the *trans*‐position to the pyridine. Assuming that the imino function is an overall stronger donor than the pyridyl fragment, the first substitution is favored here. The aminosulfonyl function is more acidic and thus will likely coordinate first, which would explain the stereochemistry, with the amidosulfonyl function *trans* to the imino group, resulting in complex **5**. The same argument can be used for complex **1**, just that the difference in acidity for the hydroxy groups is less pronounced.

This finding is reproduced by DFT calculations, which predict the experimentally isolated isomer to be 0.2 eV lower in energy for pimp and thus to be thermodynamically more stable. This is in contrast to an energy difference of 0.02 eV for the pap derivatives.^3^ This suggests that a small change from the azo group to the imino group can have a significant influence on the stereochemistry of the resulting compound.

**Figure 2:**
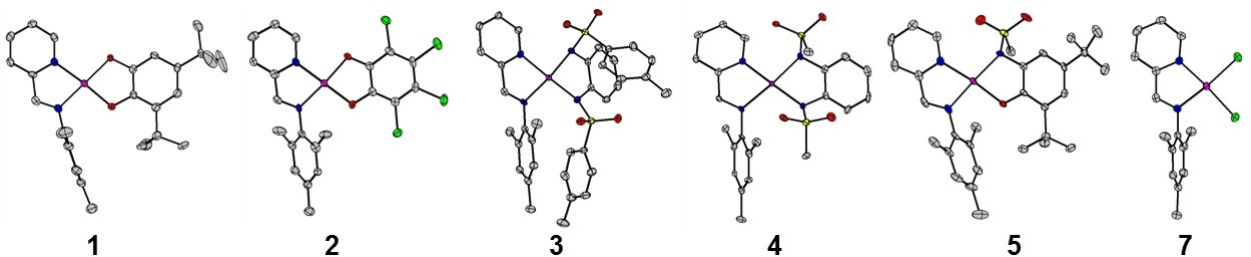
Crystal structures of compounds **1**–**5** and **7**. Color code: grey, C; pink, Pt; green, Cl; blue, N; red, O; yellow, S. Hydrogen atoms are omitted for clarity.

Additionally, all complexes and precursors were characterized by means of single‐crystal X‐ray diffraction (see Figure 2). This was especially useful for the determination of the stereochemistry of **1** and **5**. The single crystals were obtained by either vapor diffusion or evaporation of the solvent (for details, see the Experimental Section in the Supporting Information). All complexes display a distorted square planar geometry, as expected for diamagnetic platinum(II) metal centers. Compounds **1**, **3**, and **6** crystallize in the monoclinic *P*2_1_/*c* space group, whereas **2** crystallizes in the monoclinic *P*2_1_/*n* space group and **4** and **5** both crystallize in the triclinic space group *P* $\bar{1}$ .

**Figure 3:**
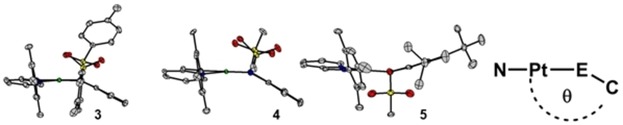
Tilt (designated by the angle *θ*; E=O or N) of the donor ligand relative to the pimp acceptor ligand for complexes **3**, **4**, and **5**. The complexes are arranged such that the mesyl group of pimp is in the foreground. Color code: grey, C; pink, Pt; green, Cl; blue, N; red, O; yellow, S. Hydrogen atoms are omitted for clarity.

Almost perfectly coplanar aromatic π‐systems for the donor and acceptor ligands are observed for **1** and **2**, featuring two oxygen donor atoms (measured between the planes spanned by the two chelates). For compounds **3** and **4** featuring a NSO_2_R donor group, we observe a deviation from coplanarity of up to 22° (designated by angle *θ*), and for compound **5** featuring an oxygen and nitrogen donor, we observe an intermediate deviation (see Figure 3). Table 1 shows selected bond lengths and angles for the discussed complexes **1**–**5**, the precursor, and the free pimp ligand. A close inspection of the bond lengths shows that the aromaticity of the catecholato ligands is retained, with bond lengths around 1.40 Å. The C1−O1 and C2−O2 bond lengths of around 1.35 Å and C1−N1 and C2−N2 of around 1.44 Å are normally observed for C−O/C−N single bonds, thus pointing to a fully reduced catecholate Q^2−^ form in all cases.^3^ The bond lengths for complexes **3** and **4** with diamidosulfonyl ligands also show bond lengths that are in good agreement with the bond lengths of the free ligand.^23^

**Table 1:** Selected bond lengths and angles for complexes **1**–**5**, (pimp)PtCl_2_ (**7**), and the free ligand **6**.

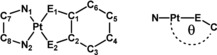							
	**1**	**2**	**3**	**4**	**5**	**6**	**7**
Pt−E_1_ [Å]^[a]^	1.991(3)	1.996(5)	2.029(2)	2.042(5)	2.036(3)	–	2.297(1)
Pt−E_2_ [Å]^[a]^	1.965(3)	1.982(5)	2.042(2)	2.033(5)	1.971(3)	–	2.292(1)
Pt−N_1_ [Å]	1.997(4)	1.968(6)	2.023(2)	2.011(5)	2.003(3)	–	2.016(3)
Pt−N_2_ [Å]	1.969(3)	1.982(6)	2.048(2)	2.046(5)	1.997(3)	–	2.001(3)
E_1_−C_1_ [Å]	1.369(5)	1.328(8)	1.450(3)	1.449(8)	1.443(5)	–	–
E_2_−C_2_ [Å]	1.354(5)	1.337(8)	1.430(3)	1.431(8)	1.352(4)	–	–
C_1_−C_2_ [Å]	1.397(6)	1.409(10)	1.390(4)	1.396(9)	1.401(5)	–	–
C_2_−C_3_ [Å]	1.404(6)	1.383(10)	1.400(4)	1.380(9)	1.401(5)	–	–
C_3_−C_4_ [Å]	1.394(6)	1.397(10)	1.391(4)	1.377(10)	1.406(6)	–	–
C_4_−C_5_ [Å]	1.397(6)	1.387(11)	1.393(4)	1.386(10)	1.402(6)	–	–
C_5_−C_6_ [Å]	1.403(6)	1.396(10)	1.393(4)	1.384(10)	1.386(6)	–	–
C_6_−C_1_ [Å]	1.386(6)	1.403(10)	1.393(4)	1.382(9)	1.389(5)	–	–
N_1_−C_7_ [Å]	1.368(4)	1.366(9)	1.368(4)	1.366(8)	1.378(5)	1.342(2)	1.369(4)
C_7_−C_8_ [Å]	1.448(4)	1.436(10)	1.448(4)	1.453(8)	1.449(6)	1.478(2)	1.448(5)
C_8_−N_2_ [Å]	1.290(5)	1.283(9)	1.290(4)	1.278(8)	1.289(5)	1.259(2)	1.289(5)
*θ*_exp_ [°]	176	176	158	161	167	–	–
*θ*_DFT_ [°]	180	180	161	168	169	–	–
*ϕ*_torsion_ [°]	−99.21	−70.48	−75.79	−99.18	72.11	−77.81	−76.87

[a] E_1_ and E_2_ are O or N.

An inspection of the bond lengths for the free pimp acceptor ligand^25^ suggests an unreduced ligand because the N1−C7, C7−C8, and C8−N2 bond lengths do not drastically change (only around 0.03 Å) upon coordination with the [PtCl_2_] fragment or subsequent catecholate coordination. This also applies to the N1−Pt and the N2−Pt bonds, which get slightly shorter when an OO‐donor ligand is employed as compared to the NN‐donors. The torsion angle *ϕ* in the pimp ligand ranges from 70° to almost 100°. The observed values agree with previous literature reports for related donor–acceptor systems^5^, ^26^ and also for related platinum chloride complexes.^9^, ^10^, ^27^

The nitrogen donors of the diamidosulfonyl ligands in complexes **3** and **4** show a different coordination geometry. A trigonal planar coordination is expected for sp^2^‐hybridized nitrogen atoms, which is characterized by a sum of 360° for all surrounding angles. The nitrogen atom in the *trans*‐position to the pyridine shows a trigonal planar coordination with angles of 355.0° (DFT: 357.1°) for **3** and 352.7° (DFT: 350.1°) for **4**. The nitrogen in the *trans*‐position to the imino function, however, shows a tetrahedral distortion as evidenced by an angle of 341.9° (DFT: 338.8°) for **3**, 339.7° (DFT: 341.5°) for **4**, and 338.6° (DFT: 343.0°) for complex **5**. This is attributed to an absence of π‐bonding of the diamidosulfonyl ligands to the metal center, when compared to unsubstituted diamidobenzenes, because similar deviations are observed for platinum(II) and rhodium(III) complexes with symmetric co‐ligands.^18^ A steric effect can most likely be ruled out, given that the less bulky mesyl (methane sulfonyl; Ms) group in **4** shows almost the same deviations as the tosyl groups in **3**.

### Cyclic voltammetry

**Figure 4:**
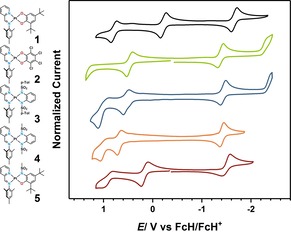
Cyclic voltammograms of complexes **1**–**5** in CH_2_Cl_2_/NBu_4_PF_6_ measured with a glassy carbon working electrode at 100 mV s^−1^ (FcH=ferrocene; FcH^+^=ferrocenium).

**Scheme 2:**
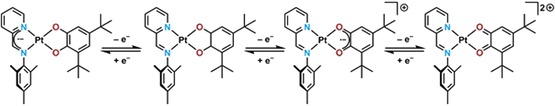
Redox processes for complex **1**.

Cyclic voltammetry reveals that complexes **1**–**5** show at least one reversible oxidation and one reversible reduction in a 0.1 m NBu_4_PF_6_ dichloromethane solution (see Figure 4). The precursor **7** shows only a single, reversible reduction at −1.35 V and no oxidation processes. This observation indicates that the reduction is centered on the pimp ligand. Depending on the substitution pattern of the donor ligand and the respective donor atoms, different additional redox events are observable within the solvent window. For cathodic processes, an electron‐deficient donor ligand renders the observation of a second reduction more likely, as observed for complexes **2** and **4**. The redox potentials for all complexes are shown in Table 2. Compound **1** shows two reversible oxidation processes most likely due to the strongly electron‐donating 3,5‐di‐*tert*‐butylcatecholato ligand, which renders the complex easy to oxidize with potentials at −0.21 V and +0.79 V vs. ferrocene/ferrocenium, with both oxidations centered on the catecholato ligand. The reduction is (as already stated) centered on the pimp ligand, which may be formally reduced twice, with the formal second reduction shifted outside of the solvent window owing to the strong donation of the catecholato ligand. Interestingly, complex **1** is the only one in the series that exhibits a significant shift in the reduction potential. The redox processes are exemplified for **1** in Scheme 2 and are also valid analogously for complexes **2**–**5**; the second reduction was not always observed under the measurement conditions.

**Table 2:** Redox potentials (*E*_1/2_ in V) vs. FcH/FcH^+^ measured in CH_2_Cl_2_ at 100 mV s^−1^ with 0.1 m Bu_4_NPF_6_ at room temperature.^[a]^

	*E*_1/2_ (1st ox.)	*E*_1/2_ (2nd ox.)	*E*_1/2_ (1st red.)	*E*_1/2_ (2nd red.)
**1**	−0.21	0.79	−1.65	–
**2**	–	0.47	−1.38	−2.36^[b]^
**3**	0.54	1.11^[b]^	−1.43	−2.40^[b]^
**4**	0.67	1.06^[b]^	−1.40	–
**5**	0.17	0.99^[b]^	−1.49	–
**6**	–	–	−1.35	–

[a] All measured with a glassy carbon electrode. [b] Potential of the peak current.

Complex **2** shows two additional redox processes (oxidation and reduction), which are completely irreversible and likely result in a decomposition of the compound. The second reduction for **2** is probably irreversible due to the imine proton, considering that the second reduction on isolobal azo‐functions is often reversible. The oxidation for all compounds likely takes place on the donor ligand, which is reflected in the fact that the oxidation potentials are dependent on the nature of the donor ligand. For complexes **3**–**5** a second oxidation is observed, which exhibits a certain degree of reversibility. The processes do not become more reversible at faster scan rates (see page S21, Supporting Information). This subset of the investigation also nicely demonstrates the tunability of the oxidation potential by variation of either donor atoms (**3** and **4** versus **5**) or the substitution pattern on the nitrogen atom (**3** versus **4**) for the investigated series.

**Figure 5:**
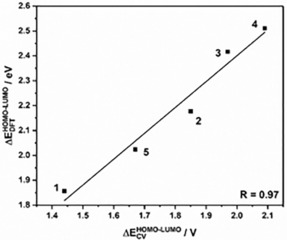
Correlation of the electrochemical HOMO–LUMO gap (ΔEHOMO-LUMOCV ) with the calculated HOMO–LUMO gap (ΔEHOMO-LUMODFT ) for complexes **1**–**5**.

The “electrochemical” HOMO–LUMO gaps of the complexes correlate almost perfectly with the calculated HOMO–LUMO gaps (B3LYP/def2‐TZVP), owing to the strong localization of the HOMO on the donor ligand and the LUMO on the acceptor ligand (see Figure 5). However, it should be mentioned that there is a slight offset in values if they are directly compared. The magnitude of the HOMO–LUMO gap is determined by the donor ligand, considering that the acceptor ligand is not changed. The more strongly electron‐withdrawing the substituents of the donor ligand are, the lower is the HOMO energy and thus the bigger the HOMO–LUMO gap. With this information, a series can be established on how electron‐withdrawing the ligands are, with the least electron‐withdrawing (or strongest donor) to the most electron‐withdrawing (or weakest donor): [Q_*t*Bu_^2−^<Q_NO_^2−^<Q_Cl_^2−^<Q_Tol_^2−^<Q_Ms_^2−^].

### UV/Vis/NIR spectroelectrochemistry

To probe the interplay of the optical and electrochemical properties, UV/Vis/NIR spectroelectrochemistry using an optically transparent thin layer electrochemical (OTTLE) cell was employed. Because the CV measurements already showed that the higher oxidation states of the compounds likely show follow‐up reactions complicating the electrochemical response, we concentrated on the first oxidation and the first reduction. Early in the investigation we observed a decay in the absorption bands when measuring the spectrum in the OTTLE cell without applying a current. We witnessed a rather quick reaction/decomposition with electromagnetic radiation in the mid‐UV range. Upon removing this part of the spectrum (200–300 nm) by using an appropriate filter, we did not observe any change of the spectrum during a simple absorption measurement in the OTTLE cell. The quantitative UV/Vis/NIR measurements in a standard cuvette did not parallel these observations. If the mid‐UV range is used during the spectroelectrochemical measurement, we observe only irreversible processes, indicating a decomposition for the simultaneous application of mid‐UV radiation and a certain redox potential.

**Figure 6:**
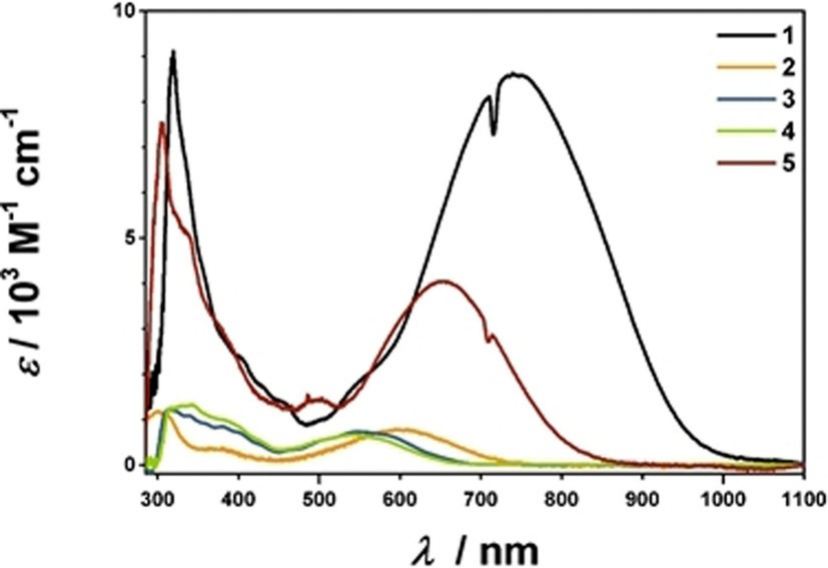
UV/Vis/NIR spectra of complexes **1**–**5** in CH_2_Cl_2_/NBu_4_PF_6_.

The UV/Vis/NIR spectra for complexes **1**–**5** are shown in Figure 6. It is evident that variation of the donor ligand has two distinct effects on the absorption spectrum. First, there is the strong decrease in the extinction coefficient for the long wavelength bands, starting from **1** with a strong donor ligand to **2** with the electron‐deficient tetrachlorocatecholato ligand. This decrease is matched by complexes **3** and **4** for which the donor functions change from “oxido” to amidosulfonyl. The “mixture” of both ligands (i.e., the complex employing the amidosulfonylphenolato ligand **5**) neatly lies in the middle between the two extremes. Second, we see a shift to higher energies (or shorter wavelengths) for complexes with electron‐withdrawing ligands. This nicely agrees with the intuitive assumption that we have to excite electrons from a ligand with an energetically lower‐lying HOMO and hence require more energy to do so. Again, complex **5** is in between the two extremes of **1** and **4**. These broad and intense bands from roughly 540 to 700 nm can be ascribed to an LL′CT process, which is also nicely reproduced by time‐dependent density functional theory (TD‐DFT) calculations.

**Figure 7:**
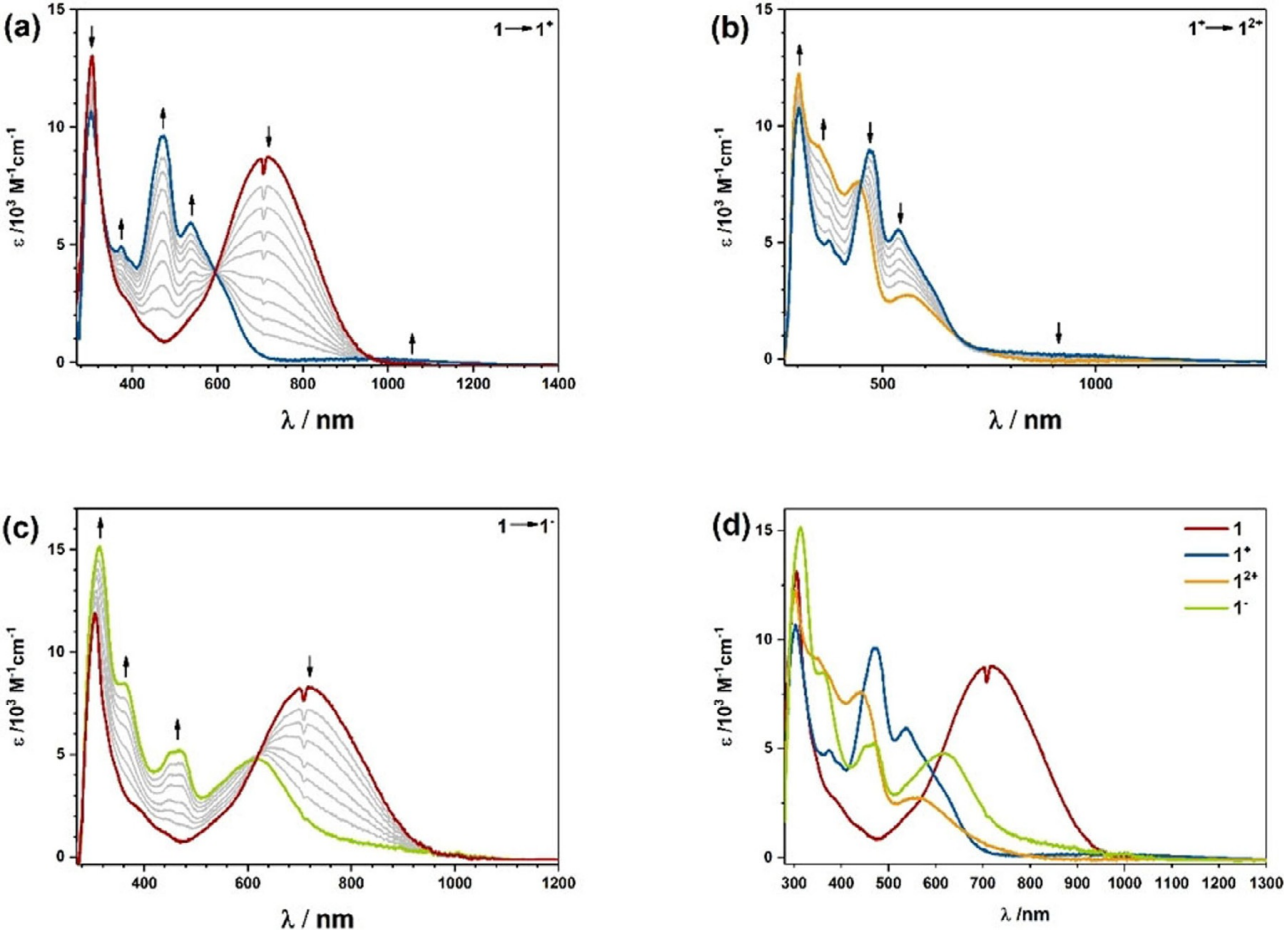
UV/Vis/NIR spectroelectrochemistry for complex **1**. a) Oxidation of 1 to 1+, b) oxidation of 1+ to 12+, c) reduction of 1 to 1−, d) comparison of UV/Vis/NIR spectra of 1, 1+, 12+ and 1−. For a detailed discussion see text.

Figure 7 shows the results of UV/Vis/NIR spectroelectrochemistry for complex **1** upon oxidation and reduction, and comparison of the spectra for **1**, **1** ^−^, **1** ^+^, and **1** ^2+^; for the different densities of the respective transitions, see pages S32–S37 (Supporting Information). Upon oxidation, the LL′CT band at 717 nm loses intensity and shifts to higher energies because less electron density is available on the donor ligand. Simultaneously we observe the increase of a weak band at around 1000 nm, which can be attributed to the intra‐ligand charge transfer (ILCT) process of the semiquinonato ligand. The new bands in the visible region for **1** ^+^ can be attributed to metal‐to‐ligand charge transfers (MLCTs) and π–π* transitions from the mesityl group to the donor ligand. Further oxidation to **1** ^2+^ results in the loss of the NIR band around 1000 nm, and the bands in the visible region shift slightly but maintain their shape. These bands of **1** ^2+^ can be assigned to MLCT processes for the now fully oxidized donor ligand.

The reduction takes place on the acceptor ligand: the LL′CT band again loses intensity, and a couple of new and relatively sharp bands arise in the visible/near UV region of the spectrum. These can be assigned to ILCT processes taking place on the pimp ligand. There are also minor d‐orbital contributions. This is observed for all the complexes, showing again that the reduction is taking place on the ligand, which is also reproduced by our TD‐DFT calculations (see pages S32–S45, Supporting Information).

In contrast to the absorption spectra of the native compounds, which look qualitatively similar, the spectra for the oxidized and reduced complexes differ substantially in some regards (see page S31, Supporting Information). If the spectra of all the singly oxidized compounds are compared, we observe rather intense NIR bands for complexes **3** ^+^, **4** ^+^, and **5** ^+^, which all feature a nitrogen donor on the semiquinonato ligand, whereas complexes **1** ^+^ and **2** ^+^ with oxygen‐only donors show no absorption in this region (Figure S78, Supporting Information). In contrast, complexes **1** ^+^ and **2** ^+^ show strong absorption in the visible region of the spectrum.

The spectra for the reduced species **1** ^−^, **2** ^−^, **3** ^−^, **4** ^−^, and **5** ^−^ look relatively similar (Figure S77, Supporting Information), which again indicates that the reduction steps are primarily pimp‐centered. We observe a relatively sharp double peak between 400 and 500 nm for all compounds, including the precursor **6**. All compounds exhibit NIR bands that also look similar, except for **1** ^−^, for which the band is shifted significantly to higher energy. For **2** ^−^, the band is extremely weak. This indicates a varying degree of influence of the donor ligand on the reduced acceptor ligand, with the influence for **1** ^−^ and **2** ^−^ (with the oxygen‐bearing donor ligands) being the most pronounced.

**Figure 8:**
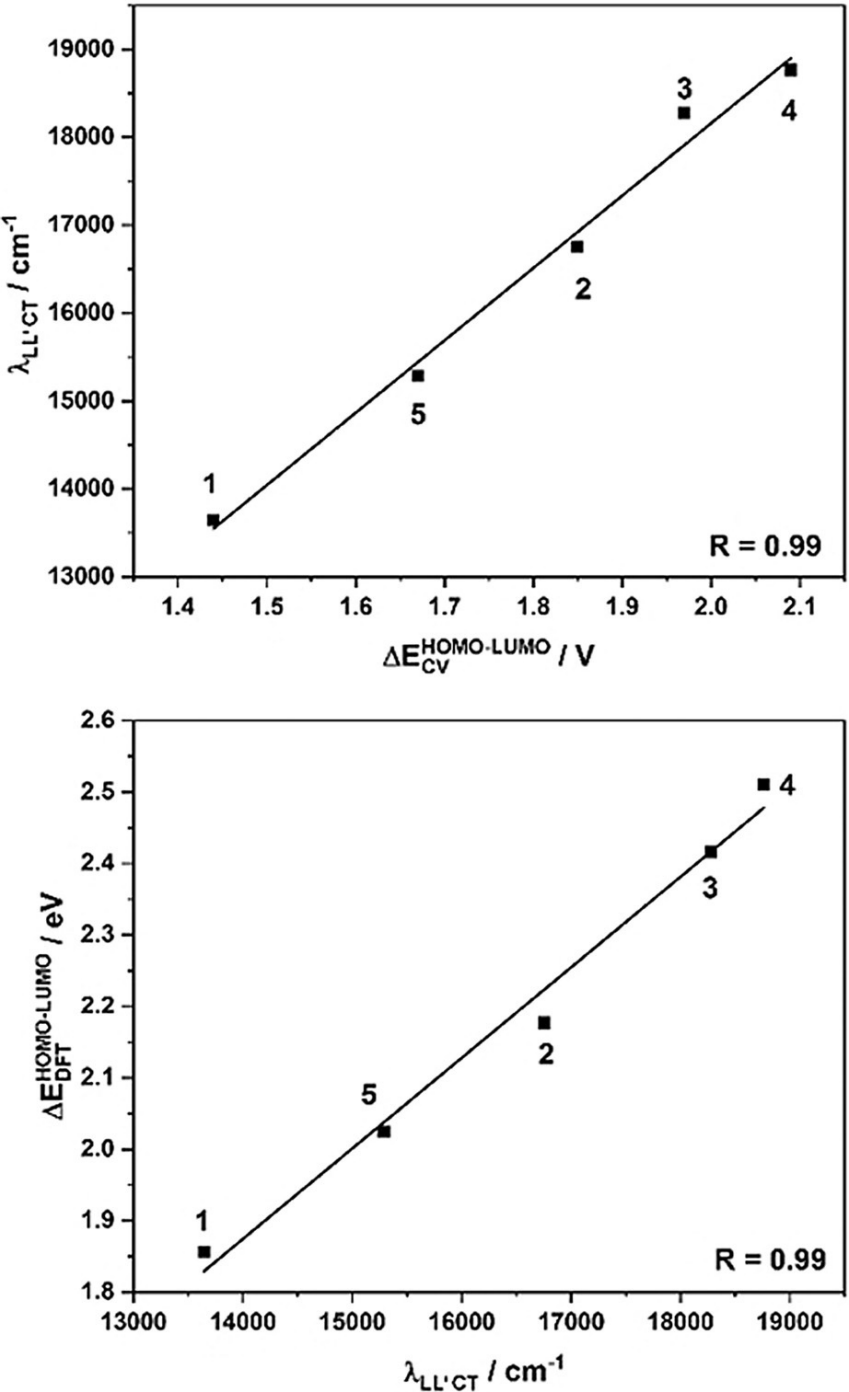
Correlation of the optical HOMO–LUMO gap (LL**′**CT) with the electrochemical HOMO–LUMO gap (left) and correlation of the calculated HOMO–LUMO gap with the optical HOMO–LUMO gap (right).

As discussed above, in these complexes, the LUMO is located on the pimp ligand and the HOMO is located on the donor ligand, and we have additionally confirmed that the low energy band can be assigned to a HOMO–LUMO transition. Thus, we can use UV/Vis/NIR spectroscopy to directly measure the HOMO–LUMO gap. The UV/Vis/NIR data can now be correlated analogously to the CV data with the DFT calculations (B3LYP/def2‐TZVP). As shown in Figure 8, the maximum of the LL′CT correlates almost perfectly with the theoretical calculations and the electrochemical data. These correlations independently validate the trend for the donor strength that we have already concluded from the CV measurements in order of decreasing donor strength: [Q_*t*Bu_^2−^<Q_NO_^2−^<Q_Cl_^2−^<Q_Tol_^2−^<Q_Ms_^2−^].

### Rearrangements in complexes 3 and 4

**Figure 9:**
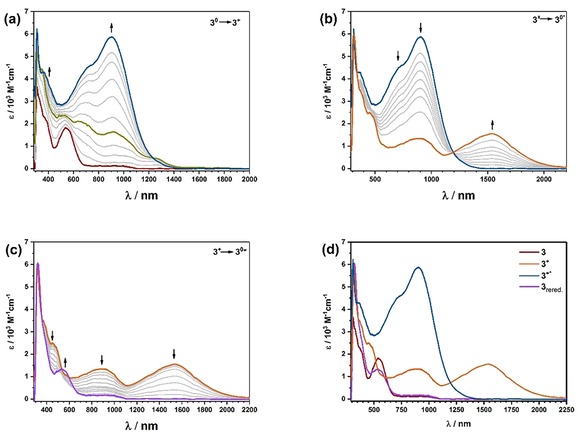
UV/Vis/NIR spectroelectrochemistry for complex **3** in CH_2_Cl_2_ **/**NBu_4_PF_6_ measured with a gold working electrode. a) Oxidation of 3 to 3+, b) re‐reduction of 3+ to rearranged 3*, c) further re‐reduction and appearance of a new band of 3+ to rearranged 3*, d) comparison of 3, 3+, 3+* and re‐reduced 3. For a detailed discussion see text.

Complex **3** showed a particular behavior during the UV/Vis/NIR spectroelectrochemical investigation. Upon oxidation of **3**, a broad and very intense band at 950 nm emerged with a shoulder at around 750 nm (see Figure 9 a). This process can be ascribed to MLCT and ILCT processes, which have been observed for diiminiosemiquinonato ligands coordinated to a metal center.^28^ Reversal of the scan direction resulted in loss of intensity of the newly emerged bands as expected. However, new bands at even lower energies around 1550 nm emerged, which had not been observed at the start of the electrolysis (see Figure 9 b). These intermediate bands vanish once the initial potential has been reached, and the complex is fully re‐reduced (see Figure 9 c). The starting spectrum is regained with roughly 80 % of its initial intensity for the LL′CT transition (see Figure 9 d), indicating a chemical reaction or substantial rearrangement of the investigated complex. A rearrangement is already evident from Figure 9 a, in which a band is observed around 1300 nm, which vanishes again in the course of the constant potential electrolysis (olive trace).

**Scheme 3:**
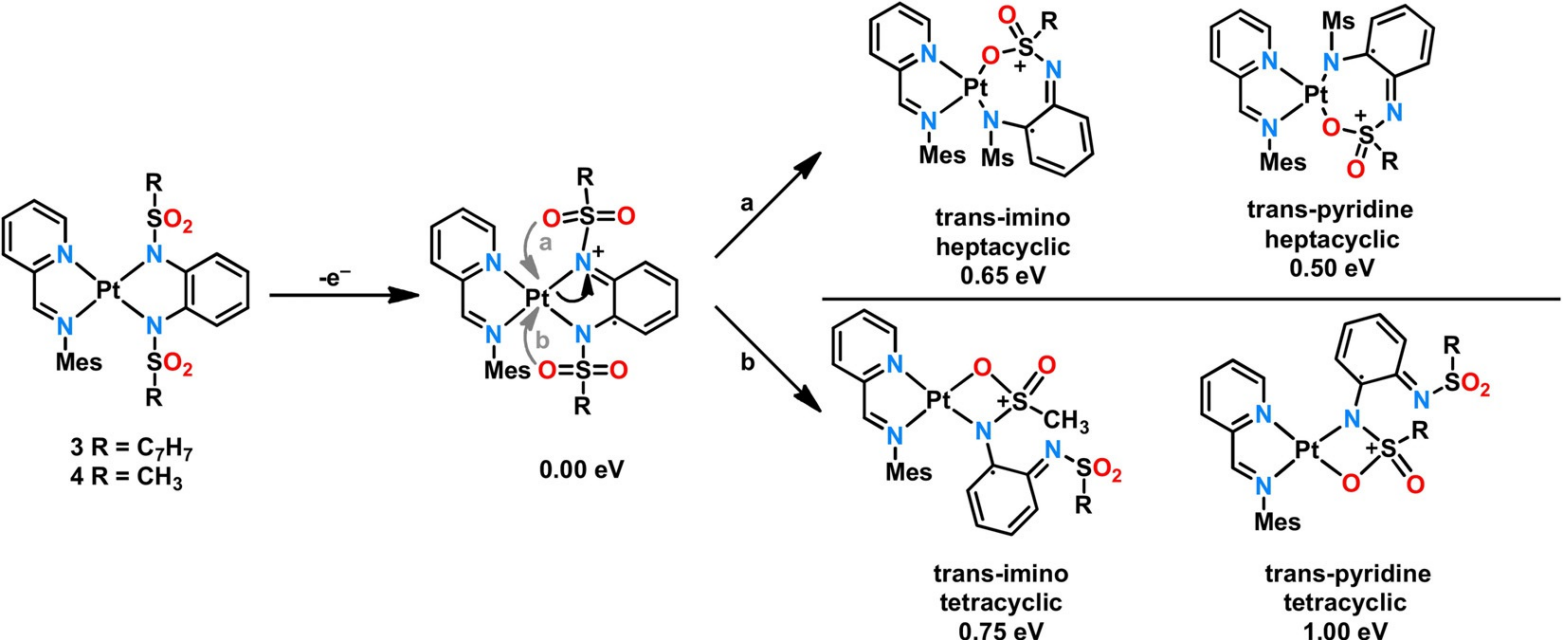
Possible isomerization for compounds **3** ^+^ and **4** ^+^ (Mes=mesityl).

Such time‐ and potential‐dependent NIR absorptions are not observed for the oxidative spectroelectrochemistry of **1**, **2**, and **5**. It is reasonable to assume that these low energy bands are caused by an ILCT^28^ of the Q_Tol_^.−^ ligand and MLCT, which is also corroborated by TD‐DFT calculations. These rearrangements possibly involve a change in the position of the tosyl groups and the coordination geometry of the nitrogen donor atoms (see discussion above). However, it is quite unlikely that simple rotational movements exert such a heavy influence on the absorption spectrum. The two independent isosbestic points at around 1200 and 530 nm also point to two different species that are present during the measurement. An alternative explanation would be the cleavage of one of the platinum–nitrogen bonds and coordination of the oxygen atoms of the sulfonyl groups. Such rearrangements have been proposed for rhodium complexes employing the same ligands by Perutz and co‐workers.^20^, ^21^ Also, a possible dimerization cannot be ruled out, as discussed recently by Chang and co‐workers.^29^ Upon oxidation of compound **3** to **3** ^+^, the electron density on the already electron‐poor bis(amidosulfonyl) ligand is further reduced, resulting in a weaker platinum–nitrogen bond. This possibly results in a linkage isomerization at the platinum center with one of the oxygen atoms of the sulfonyl group coordinating to it instead of the nitrogen atom (Scheme 3). A seven‐membered or a four‐membered chelate ring is possible with the oxygen atom coordinating in the *trans*‐position to either the pyridine or imino donor function of the pimp ligand (Scheme 3). Given that the initial UV/Vis spectrum cannot be fully recovered after re‐reduction, it is reasonable to assume that the resulting compound formed after oxidation is not very stable. Also, the DFT geometry optimization for **3** ^+^ starting from the XRD geometry of **3** did not indicate significant rearrangements. However, if the intermediates shown in Scheme 3 are used as starting points for the geometry optimization, the calculation quickly converges for all intermediates. The calculations have been performed exemplarily for complex **4** only. The relative energies of all investigated rearranged complexes predict **3** ^+^ with its original five‐membered chelate ring to be the most stable and the most unstable tetracyclic *trans*‐pyridine **3** ^+^ to be energetically disfavored by around 1 eV (Scheme 3). Bearing in mind that an energy of 1 eV corresponds to a wavelength of around 1240 nm, the light source could easily trigger this isomerization. Given the fact that the compounds decompose if a wavelength less than 300 nm is used, this photoisomerization likely occurs. For all five isomers, the optical spectra have been computed (see page S23, Supporting Information). Although some changes become apparent from the calculations, they are not sufficient to make a definitive assignment.

**Figure 10:**
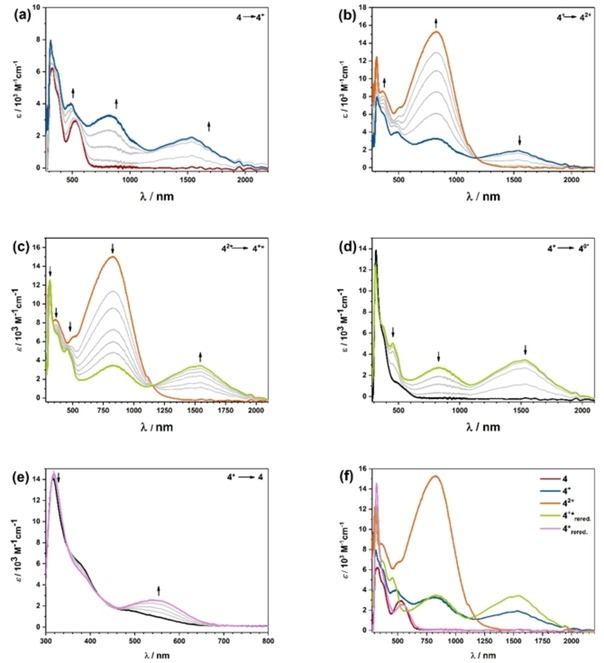
UV/Vis/NIR spectroelectrochemistry for complex **4** in CH_2_Cl_2_ **/**NBu_4_PF_6_ measured with a gold electrode. a) Oxidation of 4 to 4+, b) oxidation of 4+ to 42+, c) re‐reduction of 42+ to rearranged 4+*, d) further re‐reduction of 4+ to 4*, e) further re‐reduction and re‐emergence of the LL′CT band, f) comparison of UV/Vis/NIR spectra of 4, 4+, 42+, re‐reduced and rearranged 4+* and re‐reduced and rearranged 4*. For a detailed discussion see text.

The structurally similar complex **4** also showed some similarities to **3** during oxidative spectroelectrochemistry. For the oxidation of **4** to **4** ^+^, we observe two bands around 900 and 1500 nm of medium intensity. The spectrum for **4** ^+^ (see Figure 10 a) looks very similar to the spectrum that is obtained during the re‐reduction of **3** ^+^ to **3** (see Figure 9 c) and can be interpreted analogously (see above). Upon further oxidation, from **4** ^+^ to **4** ^2+^, the long wavelength band at 1500 nm vanishes (see Figure 10 b), whereas the band at around 900 nm strongly increases, this time resembling the spectrum of **3** ^+^ (Figure 9 a). Interestingly, the starting spectrum is almost perfectly recovered upon re‐reduction; however, the LL′CT band at around 550 nm does not increase simultaneously with the decrease in the NIR bands of **4** ^+^ at 900 and 1500 nm (see Figure 10 d).

Given the different steric demands of the tosyl and mesyl substituents, it makes sense to assume that the rearrangements for complex **4** will be less sterically hindered and thus faster than the ones for **3**. The above results thus strongly point to the operation of redox‐induced linkage isomerism in the sulfonamide‐substituted ligands, which is linked with intriguing changes in the NIR region of their absorption spectrum. To the best of our knowledge, such electron‐transfer‐driven linkage isomerism has never been observed before for metal complexes of diamidobenzenes. Unfortunately, it was not possible to probe these rearrangements by other means (e.g., infrared spectroscopy) on isolated complexes in the absence of light because the complexes were not stable towards chemical oxidizing agents ([NO]BF_4_ or AgPF_6_) under an inert atmosphere (see Figure S81, Supporting Information).

After these observations, the question arises as to why complex **5** does not display this type of linkage isomerism. A tentative explanation may be the *θ* angle between the π‐systems of the donor and acceptor ligands. This is slightly closer to 180° for **5** (see Table 1) and thus results in a better orbital interaction, which leads to a stronger stabilization of **5** ^+^ through back‐bonding of the Pt^II^ d‐orbitals, in comparison to **3** ^+^ and **4** ^+^. Additionally, one can argue that the transition state (which likely involves a tilting of the donor ligand with respect to the acceptor ligand) is already preformed in complexes **3** and **4**, and thus facilitates the rearrangement.

### EPR spectroelectrochemistry

To gain further insights into the electronic structure of the redox intermediates, EPR spectroelectrochemistry was applied. Electrolysis inside the EPR cavity led to the observation of signals for the one‐electron‐reduced and one‐electron‐oxidized forms of all complexes **1**–**5**. All spectra could be simulated (see pages S24 and S25, Supporting Information) and show a coupling to ^195^Pt (abundance of 33.3 % and nuclear spin *I*=1/2 ) with no other resolved hyperfine splitting.

The nature of the donor atoms on the donor ligand has a certain influence on the *g*‐value of the radical cationic compounds, with the *g*‐value being slightly higher for the phenylendiamide ligands than that of the catecholates or the amidophenolate ligands. The hyperfine coupling constants (hfcc) vary rather broadly from 2.73 mT for **1** to 11.90 mT for **3**, indicating varying degrees of contribution from the platinum center (see page S23, Supporting Information).

The *g*‐values of the radical anionic forms are almost the same with the only exception being complex **2**, most likely owing to the tetrachloro substitution. The same applies for the hfcc, which is on average around 9 mT, except for **2**. These values further substantiate that the first reduction is based on the acceptor ligand. The experimental observations are well‐reproduced by a spin population analysis, which correctly predicts the spin to be localized mostly on the pimp acceptor ligand for the reduced species and mostly centered on the quinoid donor ligand for the oxidized species (see page S25, Supporting Information).

### DFT calculations and electron flux

**Figure 11:**
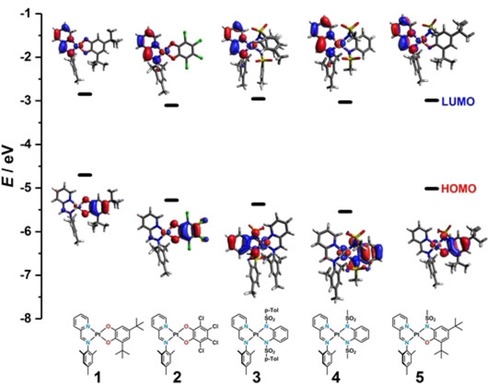
Frontier orbitals of complexes **1**–**5** along with the calculated energies.

To further rationalize the electronic structure of the complexes, DFT calculations on the B3LYP/def2‐TZVP level were employed. The optimized structures (BP86/def2‐TZVP) are in good agreement with the crystallographic data. Figure 11 shows the molecular orbital energies from these calculations along with the HOMO–LUMO gaps of complexes **1**–**5**, which we have already employed for various correlations (see above). The frontier orbitals for all of the complexes show a certain metal character but are nevertheless mostly ligand‐based and display mostly π‐character. The discussed deviation of the two nitrogen donor atoms for **3**, **4**, and **5** is also apparent in the orbital picture. The HOMOs for **1** and **2** exhibit a mirror plane (perpendicular to the π‐system and dissecting the OCCO chelate), whereas we observed a “distorted symmetry” for **3**, **4**, and **5**.

**Figure 12:**
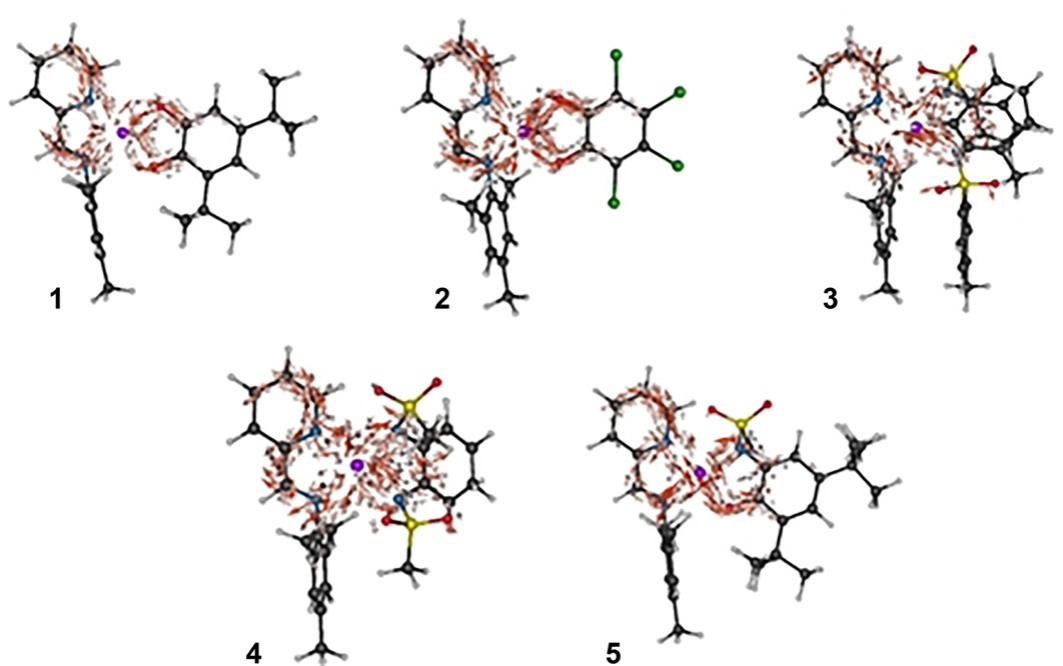
Electronic flux densities for the transition between the ground state and the first absorption band of complexes **1**–**5**. The arrows are colored according to their magnitude. The charge transfer numbers are 0.711, 0.757, 0.784, 0.774, and 0.781) for complexes **1**–**5**, respectively (carbon in black, hydrogen in white, nitrogen in blue, oxygen in red, sulfur in yellow, platinum in purple, and chlorine in green).

To characterize the ligand‐to‐ligand charge transfer mechanism for complexes **1**–**5**, it is insightful to analyze the electronic flux densities for the optically active transitions from the ground state to the first absorption band. The electronic flux density yields space‐resolved information about the flow of electrons during the excitation process. Because the first absorption band at the TD‐DFT/B3‐LYP level is dominated by a HOMO–LUMO transition, the electronic flux densities are calculated in the single‐active electron approximation. All quantities were computed using ORBKIT^30^ and depicted using ZIBAmira,^31^ as shown in Figure 12.

As discussed above, the complexes all reveal an intense ligand‐to‐ligand charge transfer for the lowest energy transition, from the various ligand donors to the iminopyridine acceptor. On the acceptor ligand of all five complexes, the electronic flux exhibits a large degree of delocalization, with a pincer‐type electron flow incoming through the platinum center, over both nitrogen atoms and to the neighboring carbon atoms through the conjugated π‐system. The delocalized flow is particularly laminar for complexes **1**, **2**, and **5**, which correlates with a laminar, pincer‐type electron flow on the donor ligand as well. The planarity between the donor and acceptor ligands leads to a simple *x*‐shaped flow of the electrons from the coordination atoms of the donor ligand over the platinum atom to the nitrogen atoms of the acceptor ligand. Despite their similarities, a stronger, more localized electron flow is observed on complex **2**. This is possibly due to the symmetry of the substituents on the phenyl ring of the donor. In contrast, the electron flow pattern at the donor ligands of complexes **3** and **4** is more intricate due to their nonplanar structure. This indicates that more electrons are available in the space between the donor ligand and the metal center, which in turns favors a through‐space mechanism for the electron transfer. The electron flow on the acceptor ligand is perturbed and reduced in intensity in both complexes but, surprisingly, the spatial extent of the electron transfer remains similar. For a more quantitative measure, the charge transfer (CT) number can be computed as the product of the donor population and the acceptor population, as in Equation (1):

(1) $$\mathrm{CT}=\langle \mathrm{LUMO}|P{}_{\mathrm{acceptor}}|\mathrm{LUMO}\rangle \langle \mathrm{HOMO}|P{}_{\mathrm{donor}}\left|\mathrm{HOMO}\right\rangle$$

where *P*_i_ are the respective Mulliken projectors on the donor and acceptor. Despite the marked differences in the electron flow mechanisms observed above, the charge transfer numbers were found to be similar in complexes **2**–**4**, ranging from 0.757 for complex **2** to 0.784 for complex **3**. Of the three planar structures, only complex **1** was found to have a slightly smaller CT number (0.711). This correlates well with the smaller electron flow observed on the donor ligand. By looking at the three largest CT numbers (complexes **3**–**5**), it appears that choosing a ligand which increases through‐space electron flow can increase the degree of charge transfer. This can come at the expense of a less laminar flow, as in complexes **3**–**4**, which we rather attribute to a structural effect.

### Application in cross‐dehydrogenative coupling reactions

Cross‐dehydrogenative coupling (CDC) has gained considerable popularity among organic chemists as an atom‐efficient method for C−C bond formation.^32^ Inspired by previous works, which utilized platinum for this reaction, we wanted to test the synthesized complexes and investigate their photocatalytic potential.^33^, ^34^ They were employed in the cross‐dehydrogenative coupling of *N*‐phenyltetrahydroisoquinoline (ISQ) with acetone and nitromethane. Special focus was put on the role of the donor atoms, which is why complexes **1**, **4**, and **5** were used for a preliminary study with an [OO], [NN], and [ON] donor ligand, respectively. We optimized the reaction conditions in terms of oxygen saturation and overall irradiation time (*t*, excitation at 360 nm). Pure oxygen was bubbled through the solution for 2 minutes, 30 minutes, or not at all (equal to atmospheric conditions). Interestingly, we observed a high yield for complex **1** after only 2 minutes of bubbling and a long irradiation time of 15 hours in nitromethane, and a considerably lower yield for the same conditions in acetone (see Table 3). If atmospheric conditions or shorter reaction times are used, the yield diminishes drastically, such that only traces of the product are isolated. Complex **4** also showed only traces under similar reaction conditions. Complex **5** interestingly showed considerably lower conversions; however, a still acceptable isolated yield of 40 % in nitromethane and only 18 % in acetone were observed. As a control experiment, the precursor **7** was also tested in the CDC, and it displayed considerable activity for the coupling of ISQ and acetone, whereas complex **1** was still superior for the coupling of ISQ with nitromethane. This may have to do with possible side reactions caused by the substantially labile chloride ligands. These results further highlight the high potential of the underdeveloped (pimp)PtX_2_ system for photocatalysis and other applications.

**Table 3:** Overview of catalytic reactions.

Entry	Catalyst	Substrate	Yield [%]	Conditions
1	**1**	MeNO_2_	88	O_2_: 2 min, *t*: 15 h
2	**1**	acetone	24	O_2_: 2 min, *t*: 15 h
3	**1**	MeNO_2_	traces (<5)	O_2_: –, *t*: 16.5 h
4	**1**	acetone	traces (<5)	O_2_: –, *t*: 16.5 h
5	**1**	MeNO_2_	traces (<5)	O_2_: 2 min, *t*: 2 h
6	**1**	acetone	traces (<5)	O_2_: 2 min, *t*: 2 h
7	**1**	MeNO_2_	stability test	O_2_: –, *t*: 2 h
8	**1**	acetone	stability test	O_2_: –, *t*: 2 h
9	**4**	MeNO_2_	traces (<5)	O_2_: 2 min, *t*: 16.5 h
10	**4**	acetone	traces (<5)	O_2_: 2 min, *t*: 16.5 h
11	no catalyst	MeNO_2_	no conversion	O_2_: 2 min, *t*: 16.5 h
12	no catalyst	acetone	no conversion	O_2_: 2 min, *t*: 16.5 h
13	**4**	MeNO_2_	traces (<5)	O_2_: 2 min, *t*: 16.5 h
14	**4**	acetone	traces (<5)	O_2_: 2 min, *t*: 16.5 h
15	**5**	MeNO_2_	traces (<5)	O_2_: 2 min, *t*: 2 h
16	**5**	acetone	traces (<5)	O_2_: 2 min, *t*: 2 h
17	**5**	MeNO_2_	traces (<5)	O_2_: 2 min, *t*: 16.5 h
18	**5**	acetone	traces (<5)	O_2_: 2 min, *t*: 16.5 h
19	**5**	MeNO_2_	40	O_2_: 30 min, *t*: 16.5 h
20	**5**	acetone	18	O_2_: 30 min, *t*: 16.5 h
21	**7**	MeNO_2_	50	O_2_: 30 min, *t*: 16.5 h
22	**7**	acetone	65	O_2_: 30 min, *t*: 16.5 h
23	**1**	MeNO_2_	no conversion	O_2_: 30 min, *t*: 16.5 h, 700 nm
24	**1**	acetone	no conversion	O_2_: 30 min, *t*: 16.5 h, 700 nm

**Figure 13:**
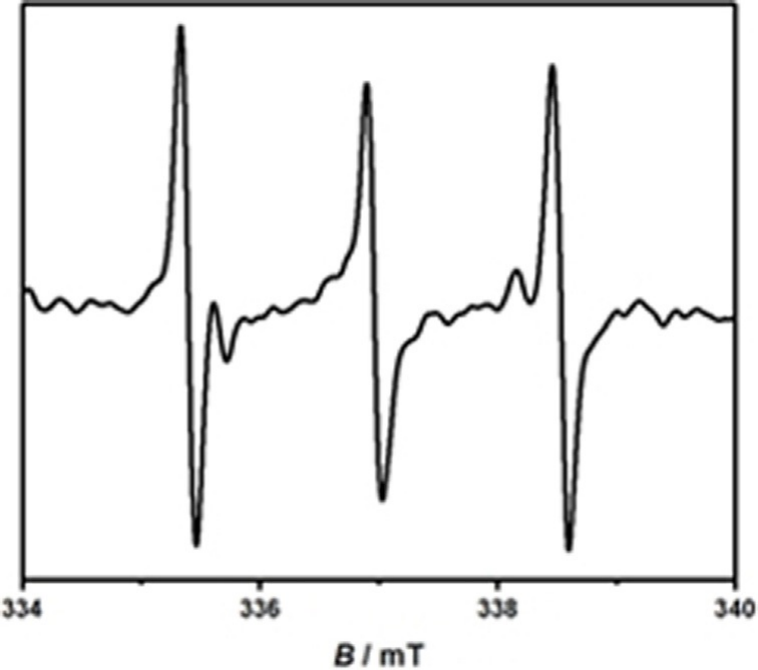
Spin trapping with PBN in an oxygen**‐**saturated DMF solution of **1**.

**Scheme 4:**
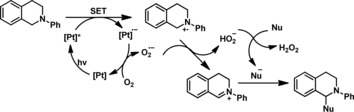
Tentative reaction mechanism for the CDC. Adapted from Chen, Fu, and co**‐**workers.^34^ (SET=single electron transfer).

To further substantiate the involvement of dioxygen as an oxidizing agent, we irradiated a solution of complex **1** in DMF (bubbled for 30 minutes) for 3 minutes with an excess of α‐phenyl‐*N*‐*tert*‐butylnitrone (PBN). The EPR spectrum shows a triplet (Figure 13), indicating the generation of a reactive oxygen species (ROS). Given that PBN is a nonspecific ROS scavenger, complex **1** could serve as a sensitizer for either singlet oxygen or the superoxide radical, as depicted in Scheme 4. To selectively probe the involvement of singlet oxygen (^1^O_2_) in the catalytic cycle, complex **1** was tested in the oxidation of 1,5‐dihydroxynaphthalene to 5‐hydroxy‐1,4‐naphthoquinone (or juglone). The observation of new bands in the visible region (see Figures S14 and S15, Supporting Information) that correspond to juglone are indicative of singlet oxygen being involved in the reaction.^35^ Complex **1** will most likely have a relatively low excited‐state oxidation potential, which will not suffice to oxidize ISQ.^36^ Thus, we repeated the catalysis at a lower excitation wavelength (around 700 nm) and observed no conversion for the reaction of ISQ with nitromethane or acetone. This supports the fact that singlet oxygen is the active species generated with this sensitizer. However, by using an irradiation wavelength of 360 nm, higher energy excited states may be generated, which have a sufficiently high potential to oxidize ISQ. More detailed studies on the photophysics of the complexes will be necessary to answer these questions.

This leads us to the conclusion that a similar catalytic cycle as already described in various literature reports is operating here (see Scheme 4).^34^, ^37^ The platinum complex is photochemically excited and quenched by the isoquinonline, resulting in the monoanionic platinum complex and a cationic tertiary amine. The molecular oxygen oxidizes the reduced platinum complex, recovering the sensitizer and redox‐mediator and generating superoxide radical, which abstracts a hydrogen atom from the oxidized isoquinoline. The hydroperoxyl radical serves as a base for the nucleophile (acetone or nitromethane), generating a monoanionic nucleophile, which combines with the cationic isoquinoline, resulting in the product. Although we have only shown an electron transfer pathway involving ^1^O_2_ in Scheme 4, the operation of an alternative energy transfer pathway cannot be completely ruled out.

Further studies will be targeted towards a deeper understanding of the mechanism and the fine‐tuning of the catalyst.

## Conclusion

We have presented a series of new platinum(II) donor–acceptor systems with the lesser used phenyliminomethylpyridine ligand and a focus on the influence of the donor ligand. The title compounds were extensively characterized by cyclic voltammetry and UV/Vis/NIR‐ and EPR spectroelectrochemistry. All complexes displayed from two up to four redox events of varying reversibility. Density functional theory reproduced the experimental absorption spectra nicely, and the dynamic electron fluxes in such systems were investigated for the first time. UV/Vis/NIR spectroelectrochemistry revealed interesting redox‐driven linkage isomerism during the oxidation of complexes **3** and **4**; an observation that, to the best of our knowledge, has been made for the first time in metal complexes of phenylenediamines. The isomerism leads to intriguing changes in the NIR region of the spectrum of the isomers. The stabilization of these isomers remains a challenge with potential applications in photocatalysts or the development of new materials. The frontier orbitals are strongly localized on the respective ligand, which resulted in an excellent correlation of the “different” HOMO–LUMO gaps. The calculation of dynamic electron flux densities provided an insight into the electron dynamics of these systems for the first time and will help in the design of better photocatalysts and optical materials. Lastly, we showed that these systems are also interesting for catalysis. Complex **1** showed high yield in the cross‐dehydrogenative coupling of nucleophiles to *N*‐phenyltetrahydroisoquinoline. Future investigations will be dedicated to the exploitation of the different redox states of complexes **1**–**5**, exploiting their switching (redox‐induced isomerism) and catalytic potential to the full, and synthesizing the nickel and palladium analogues for their potential use in catalysis and switching.

## Conflict of interest

The authors declare no conflict of interest.

## Supporting information

As a service to our authors and readers, this journal provides supporting information supplied by the authors. Such materials are peer reviewed and may be re‐organized for online delivery, but are not copy‐edited or typeset. Technical support issues arising from supporting information (other than missing files) should be addressed to the authors.

Supplementary
