# Supplementary material for: Tuning PtII‐Based Donor–Acceptor Systems through Ligand Design: Effects on Frontier Orbitals, Redox Potentials, UV/Vis/NIR Absorptions, Electrochromism, and Photocatalysis
Source: Chemistry. 2020 Jan 22;26(6):1314–27. doi: 10.1002/chem.201903700 (PMC7027812; doi:10.1002/chem.201903700)
Supplement: Supplementary file 1 — Supplementary [file CHEM-26-1314-s001.pdf]

CHEMISTRY
A European Journal

Supporting Information

Tuning Pt^{II}-Based Donor–Acceptor Systems through Ligand Design: Effects on Frontier Orbitals, Redox Potentials, UV/Vis/NIR Absorptions, Electrochromism, and Photocatalysis

Sebastian Sobottka,[a] Maite Nößler,[a] Andrew L. Ostericher,[a, b] Gunter Hermann,[c] Noah Z. Subat,[a] Julia Beerhues,[a] Margarethe Behr-van der Meer,[a] Lisa Suntrup,[a, d] Uta Albold,[a] Stephan Hohloch,[a, e] Jean Christophe Tremblay,[f] and Biprajit Sarkar*[a, g]

chem_201903700_sm_miscellaneous_information.pdf

Table of Contents

1 General Remarks and Instrumentation 3
2 Synthesis 6
2.1 Synthesis of Pt(pimp)Cl_2 **7** 6
2.2 Synthesis of Pt(pimp)(Q_{tBu}) **1** 7
2.3 Synthesis of Pt(pimp)(Q_{Cl}) **2** 8
2.4 Synthesis pf Pt(pimp)(Q_{Tos}) **3** 9
2.5 Synthesis of Pt(pimp)(Q_{Ms}) **4** 10
2.6 Synthesis of Pt(pimp)(Q_{NO}) **5** 11
2.7 General procedure for photocatalysis 12
3 NMR Spectroscopy 13
4 X-ray Crystallography 18
5 Photocatalysis 19
6 Cyclic Voltammetry 21
7 DFT calculations 22
7.1 Stereochemistry of **1** and **5** 22
7.2 Rearrangement of **4** 23
8 EPR-Spectroelectrochemistry and Spin Densities 24
9 UV-Vis-NIR-Spectroelectrochemistry 26
9.1 Spectra for **1** 27
9.2 Spectra for **2** 27
9.3 Spectra for **3** 28
9.4 Spectra for **4** 29
9.5 Spectra for **5** 30
9.6 Spectra for **6** 30
9.7 Comparison of UV/Vis/NIR spectra 31
10 (TD)DFT 33
10.1 DFT Calculation for **1** (singlet state) 33
10.2 DFT Calculation for **[1]**$^+$ (doublet state) 34
10.3 DFT Calculation for **1**$^{2+}$ (singlet state) 35
10.4 DFT Calculation for **1**$^-$ (doublet state) 37
10.5 DFT Calculation for **2** (singlet state) 38
10.6 DFT Calculation for **3** (singlet state) 40
10.7 DFT Calculation for **3**$^+$ (singlet state) 41
10.8 DFT Calculation for **4** (singlet state) 43
10.9 DFT Calculation for **4**$^+$ (doublet state) 44
10.10 DFT Calculation for **5** (singlet state) 45
11 Coordinates of optimized structures 47

1 General Remarks and Instrumentation

Unless otherwise noted, all reactions were carried out using standard Schlenk-line techniques under an inert atmosphere of nitrogen or argon (Linde, HiQ Nitrogen 5.0, Argon 5.0, purity ≥ 99.999 %). Commercially available chemicals were used without further purification. The precursor $[Ru(dmso)_4Cl_2]$[17] and the ligands[3] were synthesized according to literature procedures. Methanol was distilled from magnesium methoxide and degassed prior to use. Dry DMF was available from Acros Organics (99.8 % extra dry) and was used as received. ^{1}H NMR and $^{13}C\{^{1}H\}$ NMR were recorded on a JEOl ECS 400 spectrometer or a JEOL ECZ 400R spectrometer. Chemical shifts are reported in ppm (relative to the TMS signal) with reference to the residual solvent peaks.[18] Multiplets are reported as follows: singlet (s), duplet (d), triplet (t) quartet (q), quintet (quint), and combinations thereof. Mass spectrometry was performed on an Agilent 6210 ESI-TOF. The photocatalytic experiments have been performed with a Rayonet photochemical reactor (RMR-600) equipped with eight tube lamps (RMR-3500A, 350 nm, 4 W).

Electrochemistry

Cyclic voltammograms were recorded with a PAR VersaStat 4 potentiostat (Ametek) by working in anhydrous and degassed DMF (99.8 % extra dry, Acros Organics) or anhydrous and degassed acetonitrile ($H_2O \leq 0.005\%$, puriss., Sigma Aldrich) distilled from P_2O_5 with 0.1 M NBu_4PF_6 (dried, > 99.0 %, electrochemical grade, Fluka) as electrolyte. Concentrations of the complexes were about $1x10^{-4}$m. A three-electrode setup was used with a glassy carbon, gold or platinum working electrode, a coiled platinum wire as counter electrode, and a coiled silver wire as a pseudoreference electrode. The ferrocene/ferrocenium couple was used as internal reference.

UV/Vis/NIR spectroscopy and spectroelectrochemistry

UV/Vis/NIR spectra were recorded with an Avantes spectrometer consisting of a light source (AvaLight-DH-S-Bal), a UV/Vis detector (AvaSpec-ULS2048), and an NIR detector (AvaSpec-NIR256-TEC). Spectroelectrochemical measurements were carried out in an optically transparent thin-layer electrochemical (OTTLE)[7] cell (CaF_2 windows) with a platinum-mesh or a gold-mesh working electrode, a platinum-mesh counter electrode, and a silver-foil pseudoreference electrode. The OTTLE cell with the gold working electrode was build analogous to the one with the platinum working electrode (100 mesh woven from 0.064 mm diameter wire; 99.99% (metals basis)). Anhydrous and degassed DMF (99.8 % extra dry, Acros Organics) or anhydrous and degassed acetonitrile ($H_2O \leq 0.005\%$, puriss., Sigma Aldrich) distilled from P_2O_5 with 0.1 M NBu_4PF_6 as electrolyte was used as the solvent.

Electron paramagnetic resonance spectroelectrochemistry

EPR spectra at X-band frequency (ca. 9.5 GHz) were obtained with a Magnettech MS-5000 benchtop EPR spectrometer equipped with a rectangular TE 102 cavity. The measurements were carried out in synthetic quarz glass tubes. For EPR spectroelectrochemistry a three-electrode setup was employed using two teflon-coated platinum wires (0.005" bare, 0.008" coated) as working (or a teflon-coated gold wire (0.003" bare, 0.0055" coated) as working electrode) and counter electrode and a Teflon-coated silver wire (0.005" bare, 0.007" coated) as pseudoreference electrode.

Single-Crystal X-ray Diffraction

X-ray data for **2** and **6** were collected on a Bruker D8 Venture system at 100(2) K and for **1, 3,** on a Bruker Smart AXS using graphite-monochromated Mo Kα radiation (λα = 0.710 73 Å). The strategy for the data collection was evaluated by using the APEX2 or SMART software. The data were collected by ω- or ω- and φ-scan techniques and scaled and reduced using the APEX2, APEX3 and SADABS software. The structures were solved by intrinsic phasing or direct methods using SHELXT-2014/5 and refined using SHELXL-2014/7 by full-matrix least-squares, refining on F^2. Non-hydrogen atoms were refined anisotropically.[1]

DFT

The program package ORCA 3.0.0 or ORCA 4.1. was used for all DFT calculations.[19] Starting from the molecular structure obtained from X-ray diffraction geometry optimizations were carried out using the BP86[20] functional and no symmetry restrictions were imposed during the optimization. Subsequent single-point calculations were performed on the optimized geometries using the B3LYP functional.[21]All calculations were run with empirical Van der Waals correction (D3).[22] The restricted and unrestricted DFT methods were employed for closed and open shell molecules respectively unless otherwise stated. Convergence criteria were set to default for geometry-optimization (OPT), and tight for SCF calculations (TIGHTSCF). Relativistic effects were included with the zeroth-order regular approximation (ZORA).[23] Triple- ζ –valence basis sets (TZVP-ZORA)[24] were employed for all atoms. Calculations were performed using resolution of the identity approximation[25] with matching auxiliary basis sets[26] for geometry optimizations and numerical frequency calculations and the RIJCOSX (combination of the resolution of the identity and chain of spheres algorithms) approximation for single point calculations using the B3LYP functional.[25] Low-lying excitation energies were calculated with time-dependent DFT (TD-DFT). Solvent effects were taken into account with the conductor-like screening model (COSMO).[27] For all calculations. Spin densities were calculated according to the Mulliken population analysis.[28] The absence of imaginary frequency Spin densities, molecular orbitals and difference densities were visualized with the modified Avogadro 1.2.0 program with extended ORCA support.[29]

[19] F. Neese, *Wiley Interdiscip. Rev. Comput. Mol. Sci.* **2012**, *2*, 73.

[20] a) A.D. Becke, *Phys. Rev.* A, **1988,** *38*, 3098; b) J. P. Perdew, *Phys. Rev. B* **1986**, *33*, 8822; c) J. P. Perdew, *Phys. Rev. B* **1986**, *34*, 7406.

[21] a) A. D. Becke, *J. Chem. Phys.* **1993**, *98*, 5648; b) C. T: Lee, W. T Yang, R. G. Parr, *Phys. Rev. B,* **1988**,*37*, 785.

[22] a) S.Grimme, S.Ehrlich, L.Goerigk, *J Comput Chem.* **2011**, **32**, 1456; b) S. Grimme, J. Antony, S.Ehrlich and H.Krieg, *J. Chem. Phys.* **2010**, *132*, 154104; c) S. Grimme, *J. Comput. Chem.* **2004**, 25, 1463-1476. d) S. Grimme, *J. Comput. Chem.* **2006**, 27, 1787.

[23] C. Van Wüllen, *J. Chem. Phys.* **1998**, *109*, 392.

[24] D. A. Pantazis, X.-Y. Chen, C. R. Landis, F. Neese, *J. Chem. Theory Comput.* **2008**, *4*, 908.

[1] a)APEX2 and Bruker AXS Inc.: Madison, WI, 2012; b) Sheldrick, G. M. SADABS, Program for Empirical Absorption Correction, Version 2008/1 and University of Gottingen: Germany, **2008**.; c) SAINT+, Data Integration Engine, Version 8.27b and Bruker AXS Inc.: Madison, WI, 2012.; d) Sheldrick, G. M. SHELXL, Program for Chrystal Structure Solution and Refinement, Version 2014/7 and University of Göttingen: Germany, **2014**.; e) C. B. Hübschle, G. M. Sheldrick and B. Dittrich, *J. Appl. Crystallogr.*, 2011, **44**, 1281–1284; f) G. M. Sheldrick, *Acta Cryst. A*, 2008, **64**, 112–122; g) G. M. Sheldrick, *Acta Crystallogr., Sect. C Struct. Chem.*, 2015, **71**, 3–8.

[25] a) F. Neese, *J. Comput. Chem.* **2003**, *24*, 1740; b) F. Neese, F. Wenmohs, A. Hansen, U. Becker, *Chem. Phys.* **2009**, *356*, 98; c) O. Vahtras, J. Almloef, M. W. Feyereisen, *Chem. Phys. Lett.* **1993**, *213*, 514; d) J. L. Whitten, *J. Chem. Phys.* **1973**, *58,* 4496; e) Izsak, R.; Neese, F., *J. Chem. Phys.*, **2011**, *135*, 144105; f) F. Neese, G. Olbrich, *Chem. Phys. Lett.* **2002**, *362*, 170; g) T. Petrenko, S. Kossmann, F Neese, *J. Chem. Phys.* **2011**, 134, 054116.

[26] a) K. Eichkorn, F. Weigend, O. Treutler, R. Ahlrichs, *Theor. Chem. Acc.* **1997**, *97,* 119; b) K. Eichhorn, O. Treutler, H. Öhm, M. Häser, R. Ahlrichs, *Chem. Phys. Lett.* **1995**, *242*, 652.

[27] a) A. Klamt, G. Schüürmann, *J. Chem. Soc., Perkin Trans. 2* **1993**, 799; b) S. Sinnecker, A. Rajendran, A. Klamt, M. Diedenhofen, F. Neese, *J. Phys. Chem. A* **2006**, *110*, 2235.

[28] Mulliken, R. S., *J. Chem. Phys.* **1955**, *23*, 1833.

[29] a) M. D. Hanwell, D. E. Curtis, D. C. Lonie, T. Vandermeersch, E. Zurek and G. R. Hutchison, *J. Cheminform.*, 2012, **4**, 17; b) *Avogadro: an open-source molecular builder and visualization tool. Version 1.2.0. modified version with extended ORCA support*;

2 Synthesis

The ligands were either commercially available (H_2Q_{tBu}, H_2Q_{Cl}) or were synthesized according to literature procedures (H_2Q_{Tos},[2] H_2Q_{Ms},[1] H_2Q_{NO}[3], pimp[4]).

2.1 Synthesis of $Pt(pimp)Cl_2$ 7

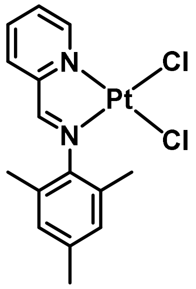

$Pt(DMSO)_2Cl_2$ (400.0 mg, 0.95mmol) and pimp **7** (217 mg, 0.95 mmol) were dissolved in 20 mL of nitromethane under ambient conditions. The yellowish solution was heated to reflux for 6 hours and subsequently cooled to room temperature. The solvent was removed on a rotary evaporator and the resulting red solid was washed with diethyl ether and dried in vacuo. The product was collected as a bright red solid (421 mg, 88%). Slow diffusion of pentane into a solution of the product in chloroform yielded bright red crystals suiTable Sfor X-ray diffraction.
^{1}H-NMR (400 MHz, CD_3CN) δ = 9.91 (dd, J = 5.8, 1.3 Hz, 1H), 8.73 (s,1H), 8.24 (td, J = 7.7, 1.5 Hz, 1H), 7.95 7.90 (m, 1H), 7.83 (ddd,J = 7.5, 5.7, 1.5 Hz,1H), 6.94 (s, 2H), 2.31 (s, 3H), 2.26 (s, 6H) ppm.
^{13}C-NMR (100 MHz, CD_3CN), δ = 172.7 (s, C=N), 156.7 (s, *o*-Pyridin-C), 149.7(s, Pyridin-C), 143.5 (s, Phenyl-C), 140.3 (s, Pyridin-C), 137.8 (s, Phenyl-C), 130.9 (s, Phenyl-C), 129.7(s, Pyridin-C), 129.1(s, Pyridin-C), 128.2 (s, Phenyl-C), 19.9 (s, Methyl-C), 16.9 (s, Methyl-C) ppm.
Elemental Analysis: Calculated for $C_{15}H_{16}Cl_2N_2Pt$: C, 36.75; H, 3.29; N, 5.71%; Found: C, 36.84; H, 3.33; N, 5.80%.
HRMS(ESI): *m/z* calc. for $[M+Na]^+$: 513.0215, found: 513.0226

[2]T. Kato, H. Masu, H. Takayanagi, E. Kaji, K. Katagiri, M. Tominaga and I. Azumaya, *Tetrahedron*, **2006**, *62*, 8458–8462.
[3]O. I. Shadyro, V. L. Sorokin, G. A. Ksendzova, O. V. Savinova, N. I. Pavlova and E. I. Boreko, *Pharm. Chem. J.*, **2012**, *46*, 414–417.
[4]S. Zai, H. Gao, Z. Huang, H. Hu, H. Wu and Q. Wu, *ACS Catal.*, **2012**, *2*, 433–440.

2.2 Synthesis of Pt(pimp)(Q_{tBu}) 1

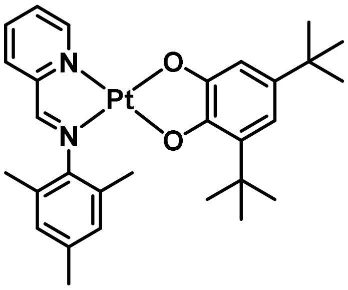

Pt(pimp)Cl_2 **6** (81 mg, 0.17 mmol) and 3,5-di-tert-butylcatechol (37 mg, 0.17 mmol) were dissolved in 20 mL acetonitrile. 0.3 mL triethyl amine (0.22 mg, 0.02 mmol) was added and the mixture was heated to reflux for 48 hours, during which the red solution became dark green. The solvent was removed yielding a dark green solid, which was then purified *via* column chromatography (Al_2O_3, dichloromethane, product eluted with DCM/CH_3CN 4:1). After removal of the solvent, a dark green solid was obtained (17 mg, 16%). Slow diffusion of pentanes into a solution of the product in tetrahydrofurane yielded single crystals suiTable Sfor X-ray diffraction.

^{1}H-NMR (400 MHz, $CDCl_3$), δ = 9.68 (d, J = 5.9 Hz, 1H), 8.82 (s, 1H), 7.96 (t, J = 8.1Hz, 1H), 7.67 (d, 1H), 7.54 (ddd, J = 7.5, 5.8, 1.3Hz, 1H), 2.36 (s, 3H), 2.31 (s,6H), 1,25 (s, 9H), 1,21 (s, 9H) ppm.

^{13}C-NMR (100 MHz, $CDCl_3$), δ = 150.4 (s, o-Pyridin-C), 144.1 (s, Phenyl-C), 137.8, 136.8 (s, Pyridin-C), 131.0, 130.8 (s, Phenyl-C), 128.7 (s, Phenyl-C), 128.1 (s, Pyridin-C), 127.0 (s, Pyridin-C), 34.8 (s, tButyl-C), 34.4 (s, tButyl-C), 29.2 (s, tButyl-C), 21.0 (s, Methyl-C), 18.0, 17.6 (s, Methyl-C) ppm.

Elemental Analysis: Calculated for $C_{29}H_{36}N_2O_2Pt$: C 54.45; H 5.67; N, 4.38%; Found: C, 54.43; H, 5.67; N, 4.40%.

HRMS(ESI): *m/z* calc. for $[M+Na]^+$: 662.2319, found: 662.2196.

2.3 Synthesis of Pt(pimp)(Q_{Cl}) 2

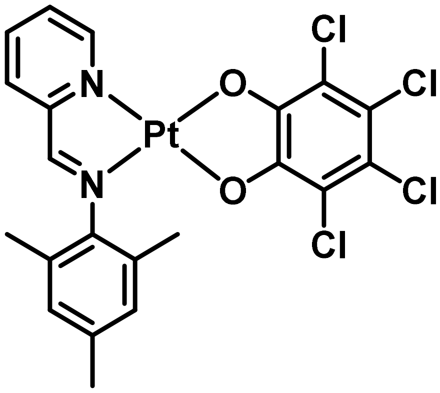

Pt(pimp)Cl_2 **6** (49 mg, 0.99 mmol) and tetrachlorocatechol H_2Q_{Cl} (25 mg, 0.99 mmol) were dissolved in 20 mL of acetonitrile, forming a reddish solution. 0.15 mL triethyl amine was added and the solution was heated to reflux, which was accompanied by a color change to dark purple. After 9 hours of reflux, the mixture was cooled to room temperature and the solvent was removed on a rotary evaporator. The resulting purple solid was then purified *via* column chromatography (Al_2O_3, dichloromethane). The product was collected as a purple fraction and yielded a purple solid after removal of the solvent (24 mg, 36%). Slow diffusion of diethyl ether into a solution of the product in acetonitrile yielded dark purple crystals suiTable Sfor X-ray diffraction.

$_1$H-NMR (400 MHz, $CDCl_3$) δ = 9.52 (d, J = 6.0 Hz, 1H), 8.74 (s, 1H), 8.03 7.97 (m, 2H), 7.75 (d, J = 7.6 Hz, 1H), 7.03 (s, 2H), 2.37 (s, 3H), 2.35 (s, 6H) ppm. Elemental Analysis: Calculated for $C_{21}H_{16}Cl_4N_2O_2Pt$: C 37.91; H 2.42; N 4.21%; Found: C 37.87; H 2.64; N 4.12%.

HRMS(ESI): *m/z* calc. for $[M+Na]^+$: 685.9506, found: 686.9488

2.4 Synthesis pf Pt(pimp)(Q_{Tos}) 3

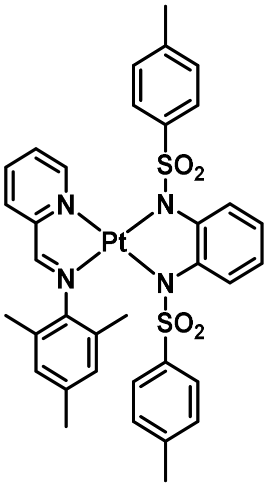

Pt(pimp)Cl_2 **6** (70.8mg, 0.14mmol) and *N*,*N'*-(1,2-phenylene)bis(*p*-toluoylsulfonamide) H_2Q_{Tos} (60.1 mg, 0.14 mmol) were dissolved in 20 ml acetonitrile. 0.3 mL triethyl amine (0.22 mg, 0.02 mmol) was added and the solution was heated under reflux for 20 h, which was accompanied by a color change from light reddish to crimson red. The reaction mixture is filtered and the solvsent in removed in vacuo. Residues of unreacted Pt(pimp)Cl_2 **6** have been removed by crystallization in dichloromethane layered with methanol. Afterwards the raw product was subjected to column chromatography (Al_2O_3, DCM, the product was dissolved with a few drops of acetonitrile). After removal of the solvent a dark violet solid was obtained (69 mg, 60%). Slow diffusion of pentanes into a solution of the product in acetone yielded dark violet crystals suitable for X-ray diffraction.

^{1}H-NMR (400 MHz, $CDCl_3$), δ = 9.95 (d, J = 6.1Hz, 1H), 8.39 (s, 1H), 8.16 (td, J = 8.1, 7.7,1.7 Hz, 1H), 7.82-7.70 (m,5H), 7.57 (d, J = 7.6Hz, 1H), 7.10 (dd, J = 8.2, 1.9 Hz, 4H), 6.92 (d, J = 8.3 Hz, 2H), 6.69-6.63 (m, 2H), 6.57 (d, J = 3.1 Hz, 2H), 6.50 (s, 2H), 2.31 (s, 3H), 2.27 (s, 6H) ppm.

^{13}C-NMR (100 MHz, $CDCl_3$), δ = 170.6 (s, C=N), 155.0 (s, o-Pyridin-C), 154,53 (s, Pyridin-C), 148.5 (s, Phenyl-C), 139.2 (s, Pyridin-C), 131.0 (s, Phenyl-C), 129.13 (s, Phenyl-C), 128.7 (s, Phenyl-C), 128.4 (s, Phenyl-C), 128.2 (s, Phenyl-C), 127.8 (s, Phenyl-C), 123.7 (s, Phenyl-C), 122.6 (s, Phenyl-C), 121.9 (s, Pyridin-C), 121.1(s, Phenyl-C) 21.6 (s, Methyl-C), 20.88 (s, Methyl-C), 18.8 (s, Methyl-C)ppm

Elemental analysis calc. for $C_{35}H_{34}N_4O_4PtS_2$: C 50.41; H 4.11; N 6.72; S 7.69%, found: C 49.67; H 4.14; N 6.06; S 7.15%,

HRMS(ESI): *m/z* calc. for [**3**+Na]$^+$: 856.1564, found:856.1548.

2.5 Synthesis of Pt(pimp)(Q_{Ms}) 4

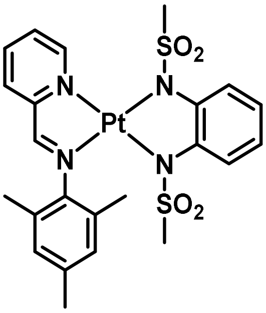

Pt(pimp)Cl_2 **6** (74 mg, 0.15 mmol) and *N*, *N*'-(1,2-phenylene)dimethanesulfonamide H_2Q_{Ms} (40 mg, 0.15 mmol) were dissolved in 10 ml acetonitrile. 0.3 mL triethyl amine (0.22mg, 0.02mmol) was added and the solution was heated under reflux for 6 h, which was accompanied by a color change from light reddish to crimson red. The solvent is removed in vacuo resulting in a dark red residue. Residues of unreacted Pt(pimp)Cl_2 **6** have been removed by crystallization in dichloromethane layered with methanol. Afterwards the raw product was subjected to column chromatography (alumina, DCM). After removal of the solvent a dark red solid was obtained (44.5mg, 43,5%). Slow diffusion of pentanes into a solution of the product in acetone yielded dark violet crystals suitable for X-ray diffraction.

^{1}H-NMR (500 MHz, $CDCl_3$), δ = 9.75 (d, J = 6.7 Hz, 1H), 8.51 (s, 1H), 8.15 (dt, J = 7.7, 1.4 Hz, 1H), 7.81 (d, J = 8.5 Hz, 1H), 7.73 (ddd, J = 7.4, 5.8, 1.5 Hz, 1H), 7.52, 7.00 (s, 2H), 6.86 (d, J = 1.4Hz, 2H), 6.82-6.78 (m, 4H), 3.01 (s, 6H), 2.40 (s, 6H), 2.32 (s, 3H) ppm.
^{13}C-NMR (CDCl, 100 MHz): δ = 169.9 (s, C=N), 155.2 (s, Pyridin-C), 154.4 (s, o-Pyridin-C), 149.1 (s, Phenyl-C), 146.8 (s, Pyridin-C), 145.4 (s, Phenyl-C), 139.2 (s, Pyridin-C), 128.34 (s,1C,Phenyl-C), 127.1 (s, Phenyl-C), 124.9 (s, Pyridin-C), 124.5 (s, Phenyl-C), 123.18(s,2C,Phenyl-C), 120.5 (s, Phenyl-C), 43.6, 40.5 (s, Methyl-C), 20.9 (s, Methyl-C), 18.7 (s, Methyl-C) ppm.
Elemental analysis calc. for $C_{23}H_{26}N_4O_4PtS_2$: C 40.52; H 3.84; N 8.22; S 9.41%, found: C 40.78; H 3.93; N 8.21; S 9.44%,
HRMS(ESI): *m/z* calc. for $[M+H]^+$: 682.1118, found: 682.1091.

2.6 Synthesis of Pt(pimp)(Q_{NO}) 5

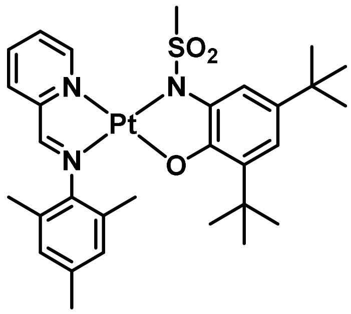

The synthesis was carried out analogously to **1**. Pt(pimp)Cl_2 **6** (49 mg, 0.1 mmol) and H_2Q_{NO} (26 mg, 0.1 mmol) are dissolved in 15 ml acetonitrile, triethyl amine (0.3 mL) is added. The reaction mixture is heated under reflux for 20 h and the solvent is evaporated afterwards. The green raw product is subjected to column chromatography (alumina, DCM/acetonitrile). The reaction yielded 39 mg (54%).

Slow diffusion of hexanes into a solution of the product in acetone and subsequent evaporation of the solvent yielded dark blue crystals suitable for X-ray diffraction.

^{1}H-NMR (400 MHz, $CDCl_3$), δ = 9.85 (d, J = 5.0 Hz, 1H), 8.80 (s, 1H), 8.09-8.04 (m, 1H) 7.78 (d, J = 7.6 Hz, 1H), 7.65-7.60 (m, 1H), 6.96 (s, 2H), 6.72 (d, 2H), 2.75 (s, J = 2.3 Hz, 3H); 2.35 (s, 3H), 2.29 (s, 6H), 2.00 (s, 9H), 1.25 (s, 9H) ppm.

^{13}C-NMR (100 MHz, $CDCl_3$): 167,13 (1C, C=N); 156,04 (1C *o*-Pyridin-C); 143,16 (1C, Phenyl-C); 138,14 (1C, Phenyl-C); 137,65 (2C, Phenyl-C); 130,46 (2C, Phenyl-C); 129,27 (1C, Pyridin-C); 128,55 (1C, Pyridin-C); 126,89 (1C, tert-butyl-C); 118,74 (2C, Phenyl-C); 38,74 (1C, Methyl-C); 34,43 (1C, tert-butyl-C); 32,13 (3C, tert-butyl-C); 21, 08 (1C, Methyl-C); 17,92 (2C, Methyl-C) ppm.

Elemental analysis calc. for $C_{30}H_{39}N_3O_3PtS$•0.5 C_6H_{14}: C 52.16; H 6.10; N 5.53; S 4.22%, found: C 52.02; H 5.96; N 5.51; S 4.00%

HRMS(ESI): *m/z* calc. for $[M+K]^+$: 755.1991, found: 755.1876.

2.7 General procedure for photocatalysis

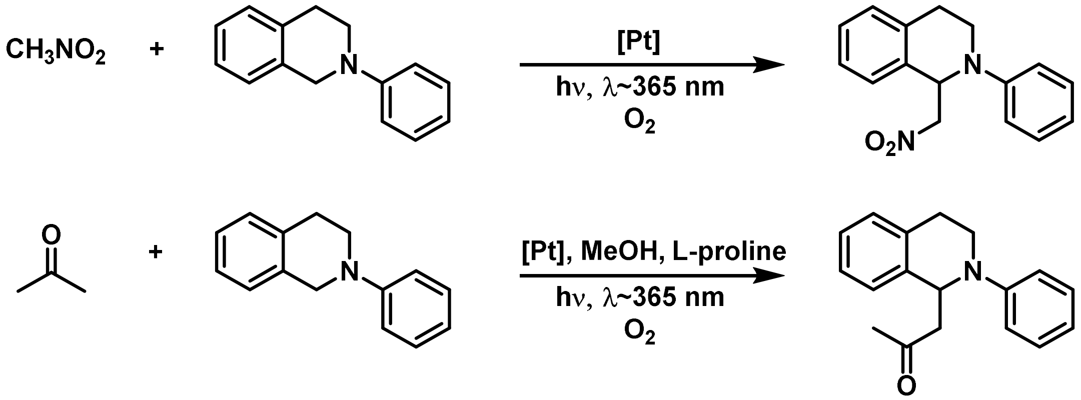

Figure S1. Photocatalytic reactions. [Pt] signifies the respective catalysts (**1**, **4** or **5**).

The photocatalytic experiments have been conducted with the photochemical reactor RMR-600 Rayonet, Southern New England Ultraviolet Company). The photocatalyst (complex **1**, **4** or **5**) and *N*-phenyl-1,2,3,4-tetrahydroisoquinoline and auxilaries were dissolved in either 10 ml nitromethane or acetone/methanol (6 ml / 4 ml), respectively. The solution was purged for a certain time with elemental oxygen (see table) and –depending on the experiment– irradiated for a specific time (see manuscript). For isolation of the product the solvent was evaporated with a rotary evaporator and was subsequently subjected to column chromatography (silica, hexanes/ethyl acetate gradient 1:0 to 0:1).

The NMRs of the products are consistent with the literature[5] and are depicted below.

[5] D. P. Shelar, T.-T. Li, Y. Chen and W.-F. Fu, *ChemPlusChem*, 2015, **80**, 1541.

3 NMR Spectroscopy

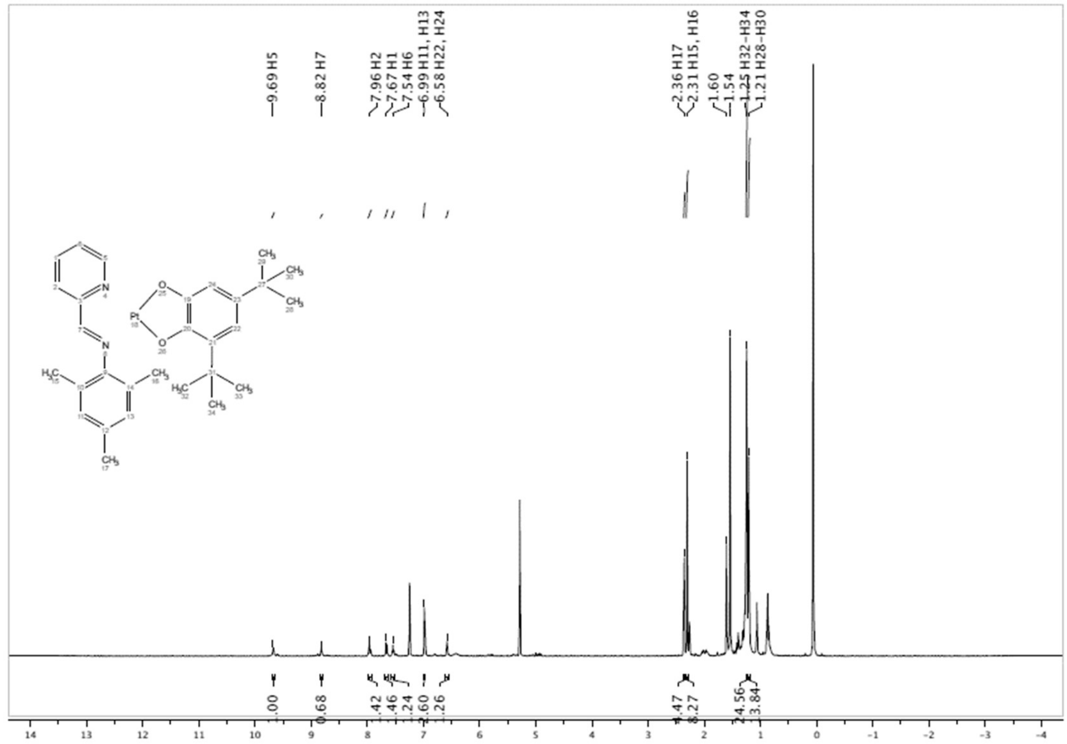

Figure S2. ^{1}H-NMR of **1** in $CDCl_3$.

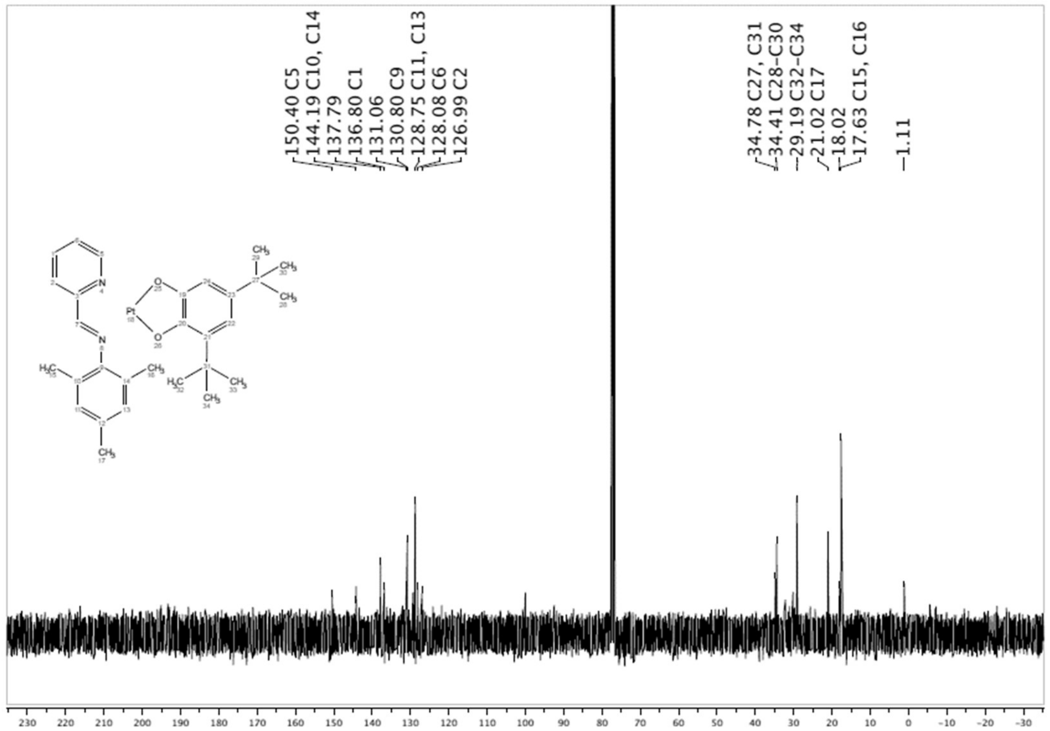

Figure S3. ^{13}C-NMR of **1** in $CDCl_3$.

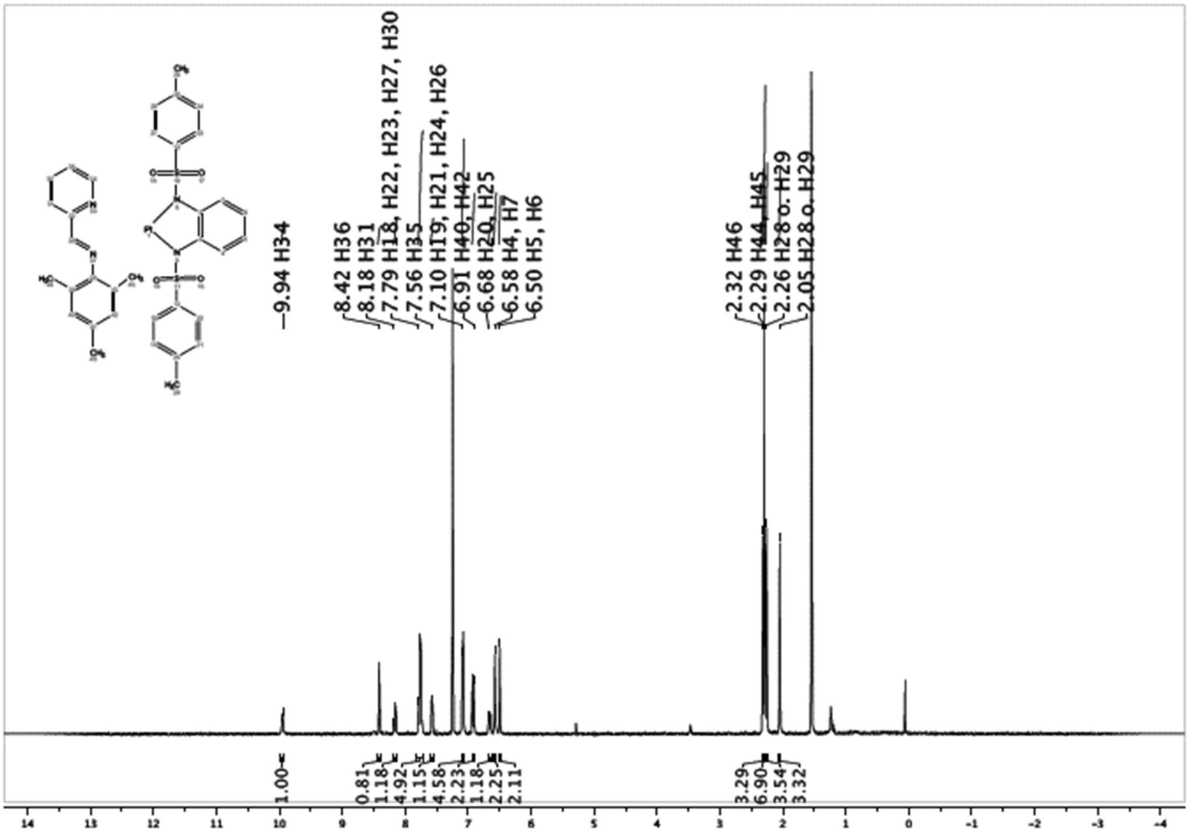

Figure S4. ^{1}H-NMR of **3** in $CDCl_3$.

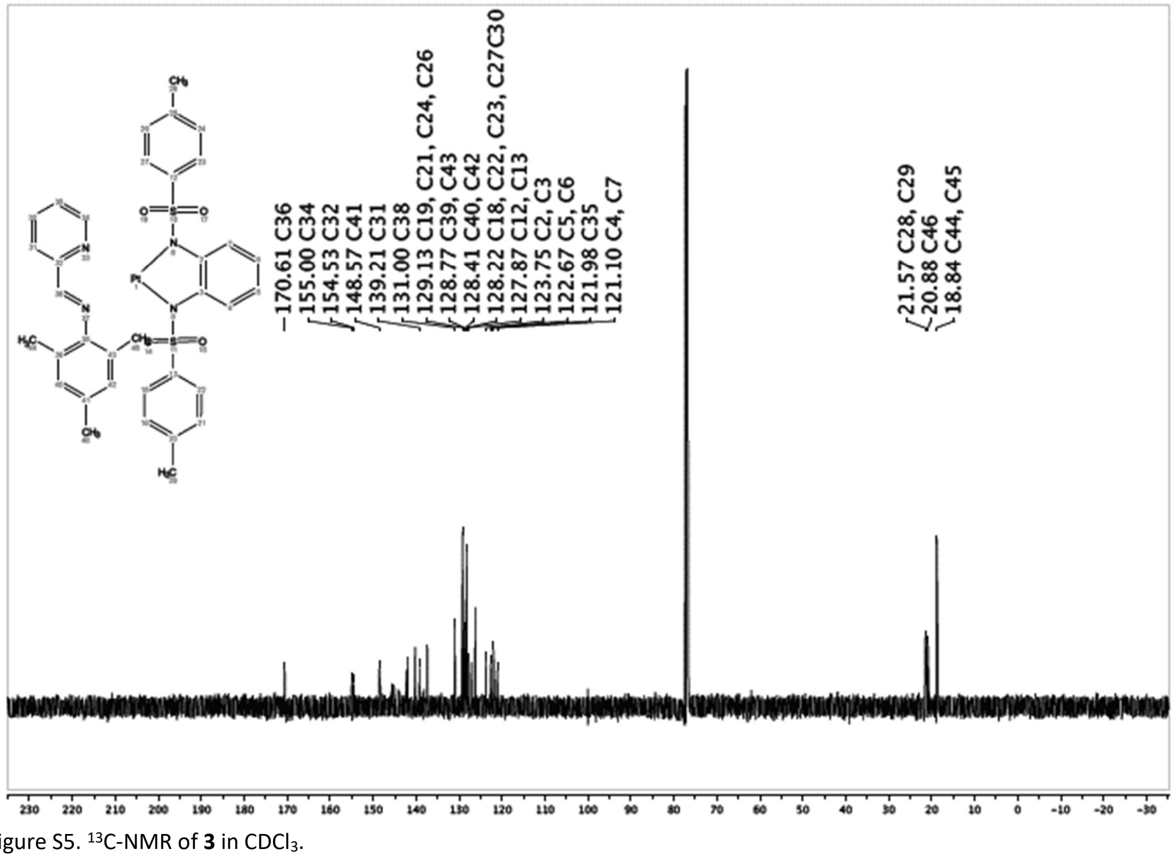

Figure S5. ^{13}C-NMR of **3** in $CDCl_3$.

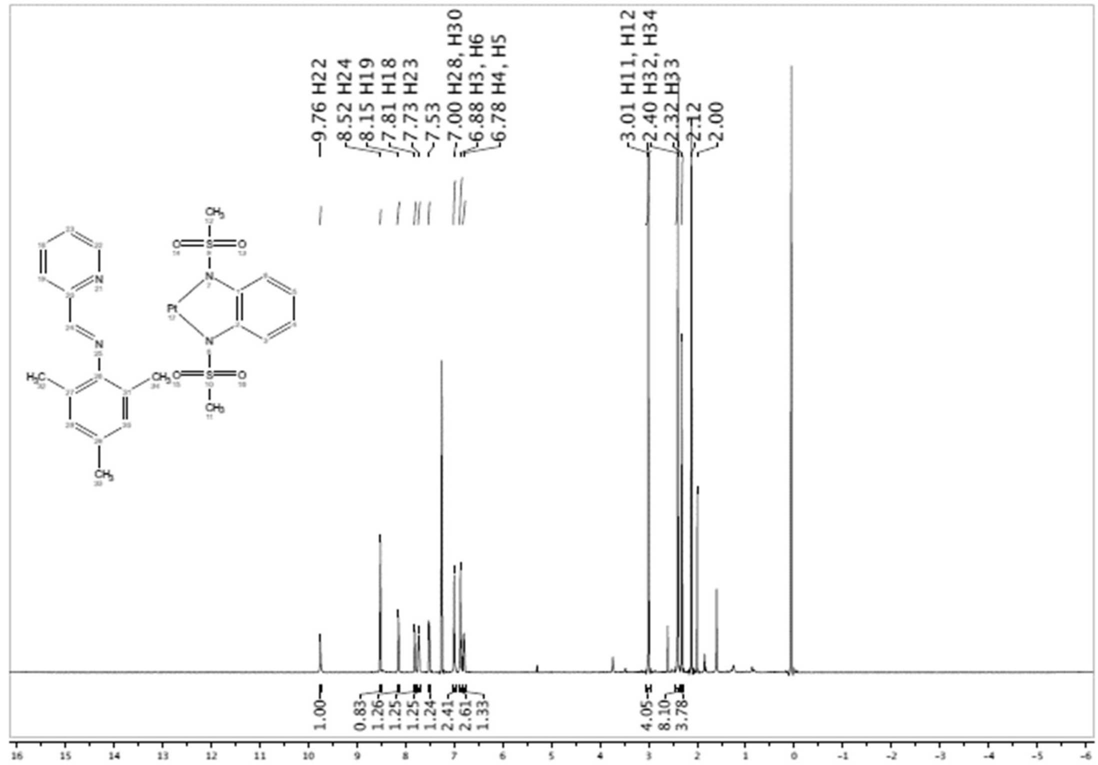

Figure S6. ¹H-NMR of 4 in $CDCl_3$.

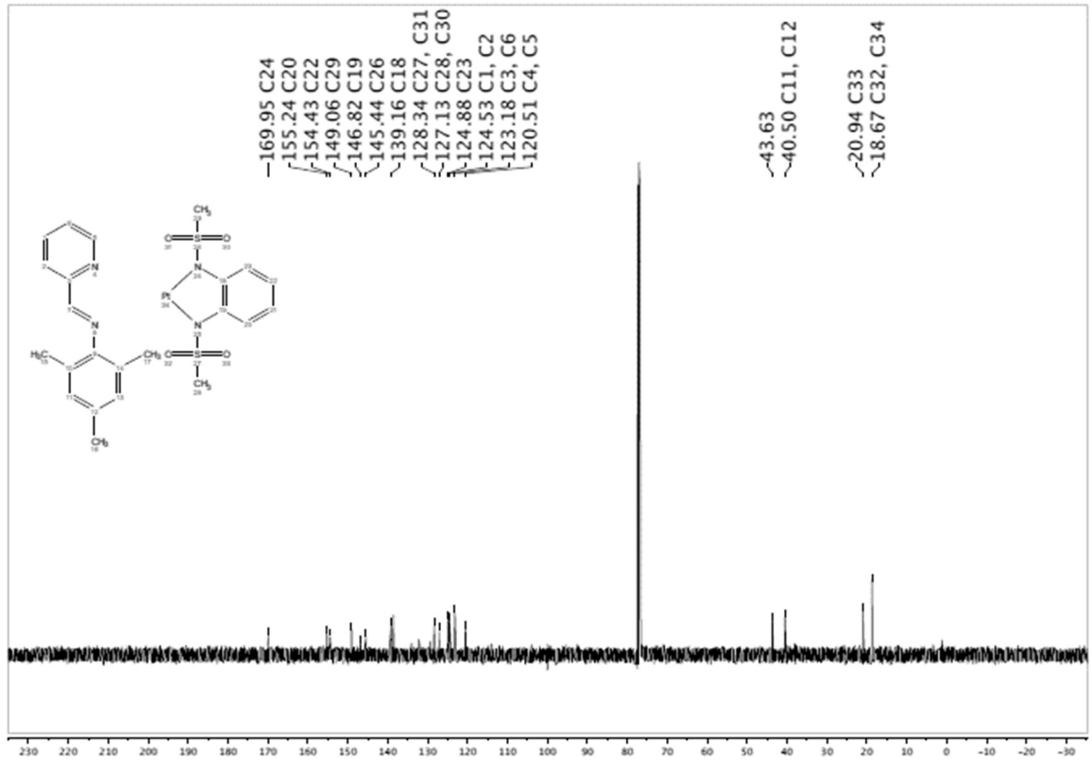

Figure S7. ¹³C-NMR of **4** in $CDCl_3$.

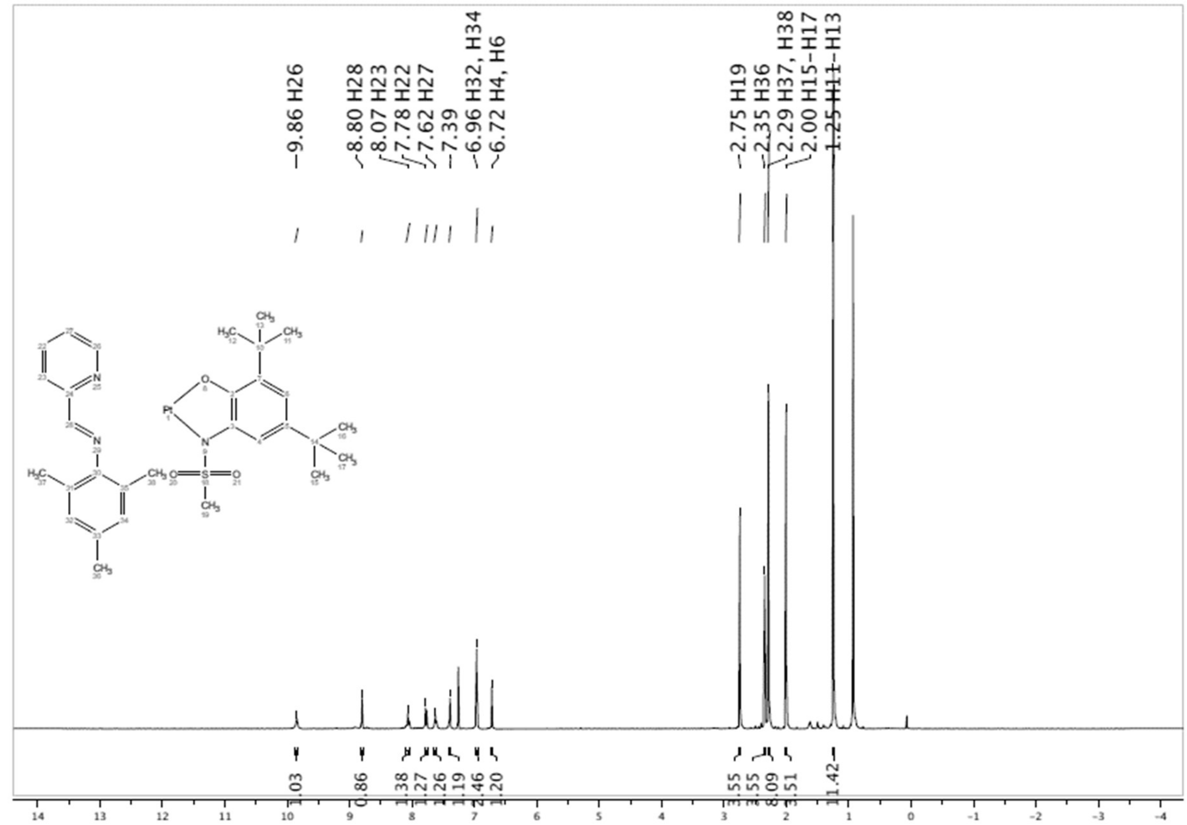

Figure S8. ^{1}H-NMR of **5** in $CDCl_3$.

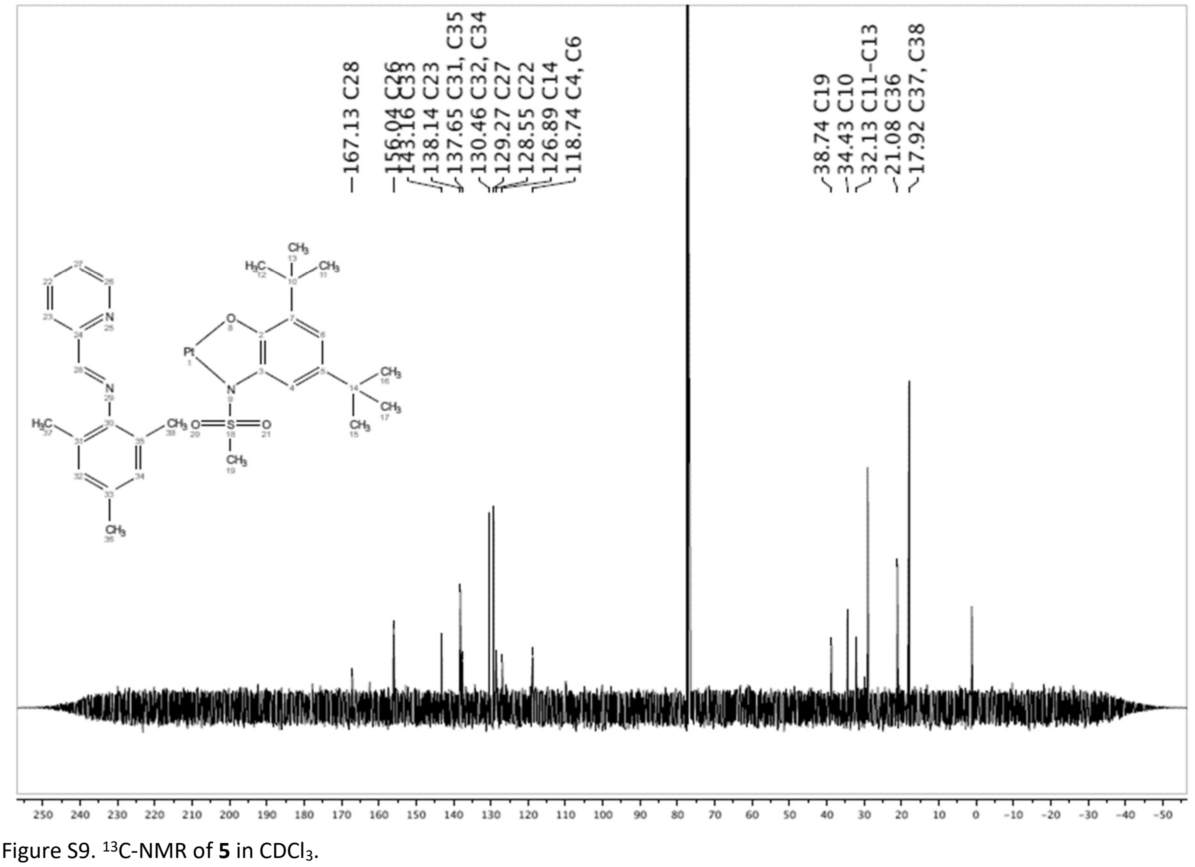

Figure S9. ^{13}C-NMR of **5** in $CDCl_3$.

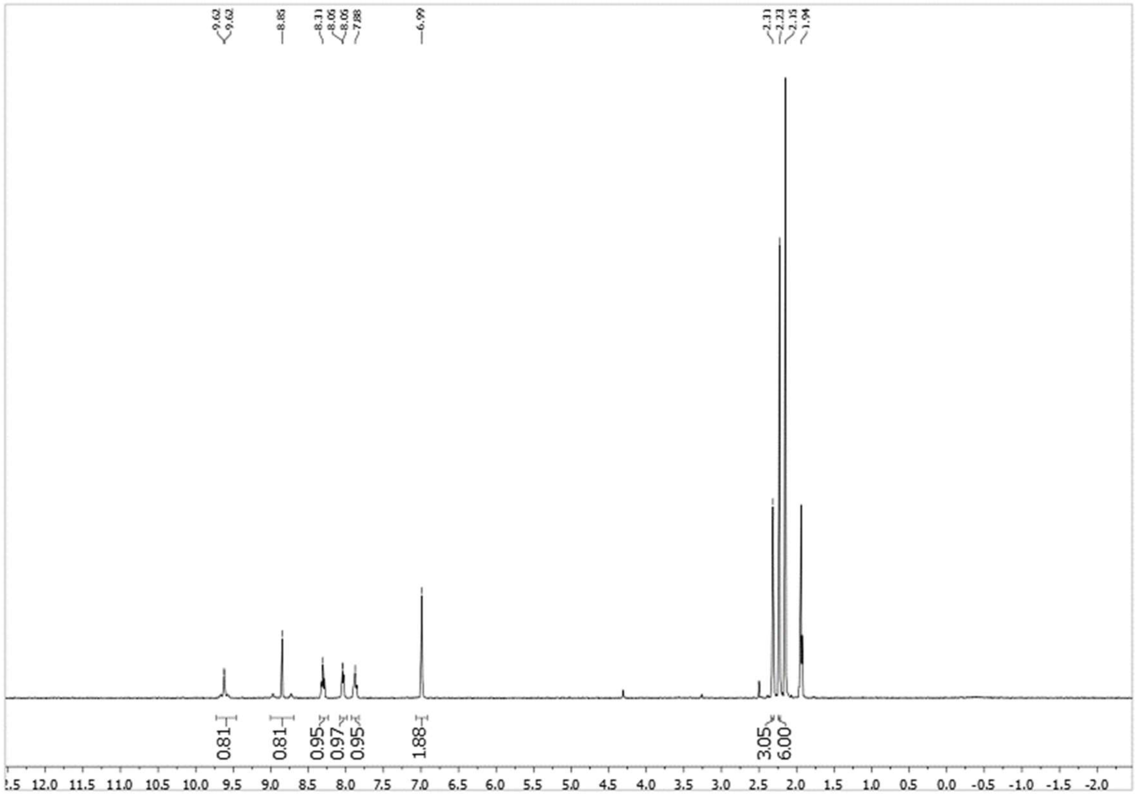

Figure S10. ^{1}H-NMR of **6** in $CDCl_3$.

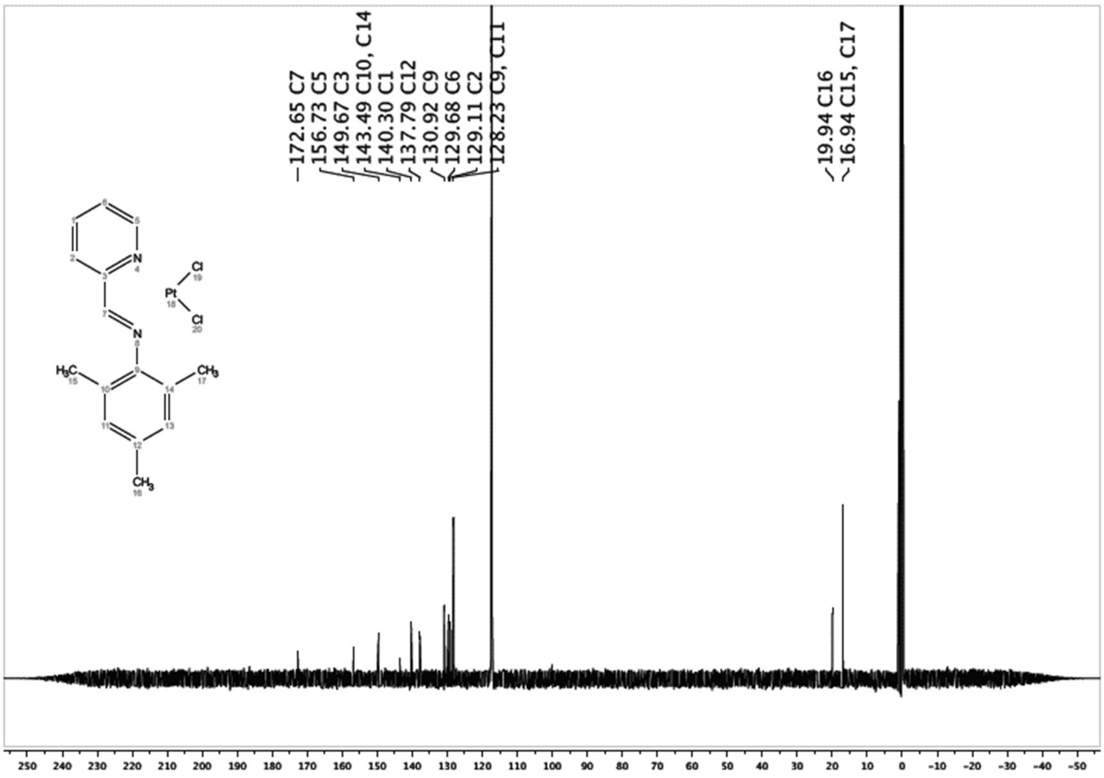

Figure S11. ^{13}C-NMR of **6** in $CDCl_3$.

4 X-ray Crystallography

Table SS1 Crystallographic details for 1, 2, 3, 4, 5 and 6.

	1	2	3	4	5	7
CCDC#	1917207	1920730	1920732	1920733	1920734	1920735
Chemical formula	$C_{29}H_{36}N_2O_2Pt$	$C_{21}H_{16}Cl_4N_2O_2Pt$	$C_{35}H_{34}N_4O_4PtS_2$	$C_{23}H_{26}N_4O_4PtS_2$	$C_{30}H_{39}N_3O_3PtS$ • 0.5 C_6H_{14}	$C_{15}H_{16}Cl_2N_2Pt$
M_r	639.69	665.25	833.87	681.69	759.88	490.29
Crystal system	monoclinic	monoclinic	monoclinic	triclinic	triclinic	monoclinic
Space group	P 2_1/c	P 2_1/n	P 2_1/c	$P\bar{1}$	$P\bar{1}$	P 2_1/c
a (Å)	14.4768(7)	11.125(2)	17.7955(5)	9.6699(6)	10.2672(15)	9.7378(18)
b (Å)	12.8888(6)	14.694(3)	12.8057(4)	17.6926(12)	17.956(3)	12.806(2)
c (Å)	16.1874(8)	13.207(3)	14.3029(4)	21.7192(14)	19.004(3)	12.279(2)
α (°)	90	90	90	106.865(2)	72.154(5)	90
β (°)	115.336(2)	93.018(4)	102.9310(10)	94.247(2)	89.488(5)	91.733(4)
γ (°)	90	90	90	90.264(2)	87.200(5)	90
V (Å^3)	2729.9(2)	2156.0(7)	3176.74(16)	3544.9(4)	3330.9(8)	1530.6(5)
Z	4	4	4	6	4	4
D (g/cm^3)	1.556	2.049	1.744	1.916	1.515	2.128
F(000)	1272	1272	1656	2004	1532	928
Radiation Type	MoK_α	MoK_α	MoK_α	MoK_α	MoK_α	MoK_α
Crystal size	0.13x0.05 x0.02	0.20x0.19 x0.17	0.45x0.38 x0.05	0.20x0.05 x0.01	0.37x0.08 x0.07	0.15x0.10 x0.09
μ (mm^{-1})	5.167	7.026	4.595	6.153	4.311	9.507
Meas. Refl.	19348	14217	28040	74165	137801	19956
Indep. Refl.	4944	4918	7817	17639	13732	3511
Obsvd. [$I > 2\sigma(I)$] refl.	3944	3536	6717	13034	12019	3079
R_{int}	0.0544	0.0550	0.0412	0.0986	0.0492	0.0317
R [$F^2 > 2\sigma(F^2)$]	0.0297	0.0444	0.0256	0.0473	0.0295	0.0183
wR(F^2)	0.0529	0.0950	0.0565	0.0716	0.0643	0.0480
S	1.042	1.095	1.010	1.036	1.101	1.089
$\Delta\rho_{max}$, $\Delta\rho_{min}$ (e Å^{-3})	0.631 -0.891	2.436 -1.440	1.400 -1.213	1.486 -1.961	2.839 -1.076	1.414 -0.919

5 Photocatalysis

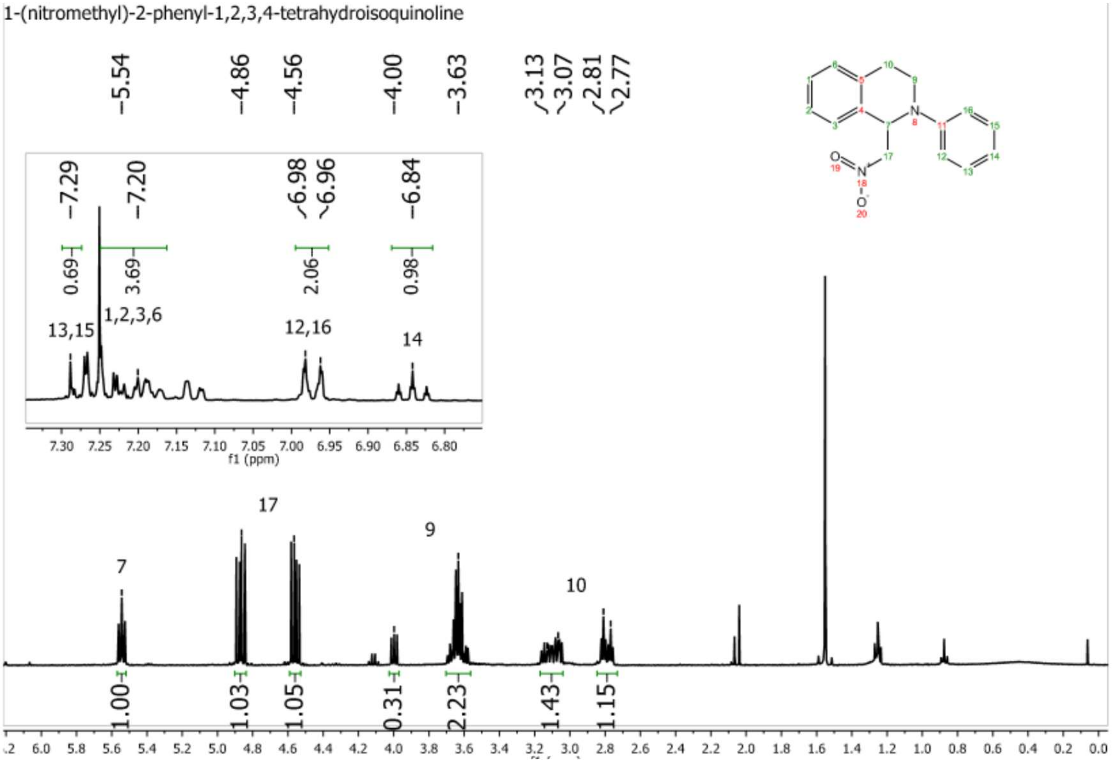

Figure S12. ^{1}H-NMR of 1-(nitromethyl)-2-phenyl-1,2,3,4-tetrahydroisoquinoline in $CDCl_3$.

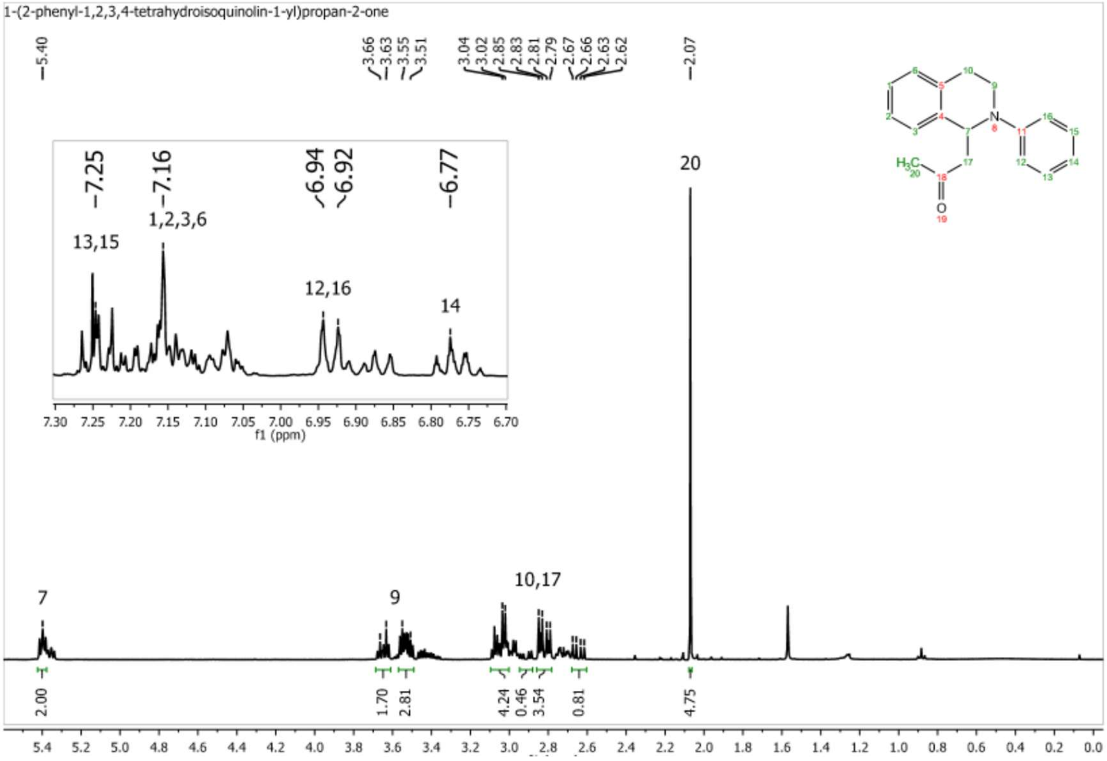

Figure S13. ^{1}H-NMR of 1-(2-phenyl-1,2,3,4-tetrahydroisoquinolin-1-yl)propan-2-one in $CDCl_3$.

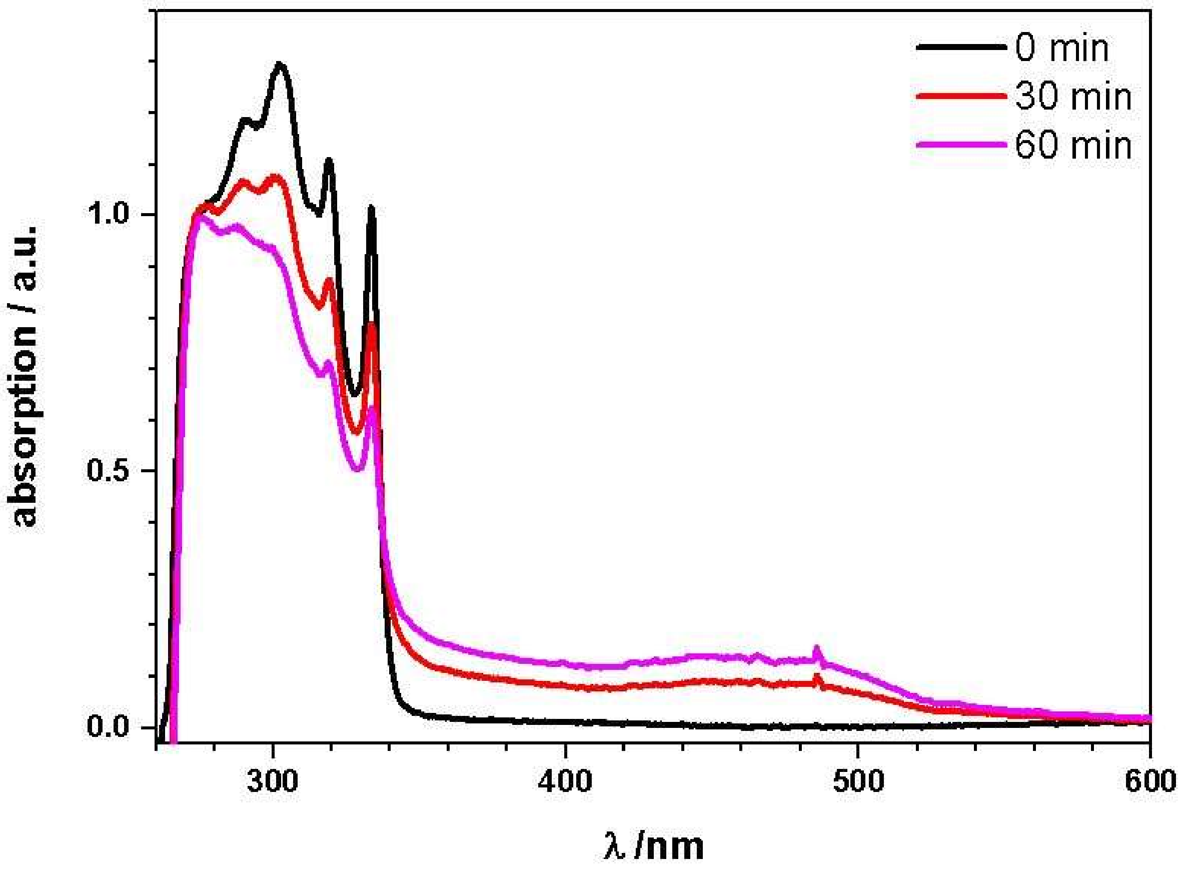

Figure S14. UV/Vis spectra following the oxidation of 1,5-dihydroxynaphthalene to juglone using molecular oxygen and complex **1** as sensitizer in a DMF solution.

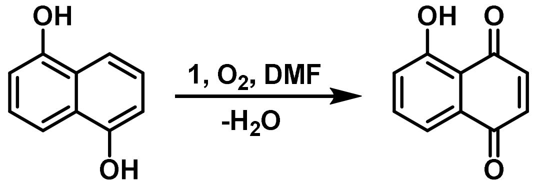

Figure S15. Oxidation of 1,5-dihydroxynaphthalene to juglone. Complex **1** was used as a sensitizer to generate singlet oxygen.

6 Cyclic Voltammetry

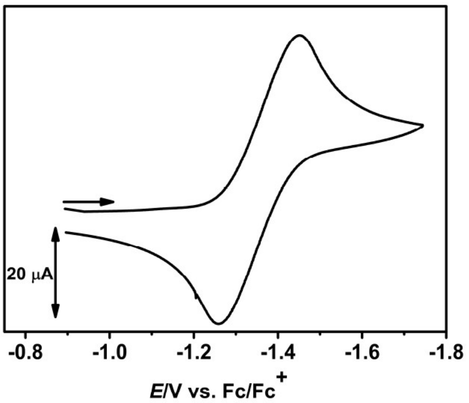

Figure S16. Cyclic voltammogram of **6** measured in 0.1 M Bu_4NPF_6/CH_2Cl_2 at room temperature and a scan rate of 100 mVs^{-1} with glassy carbon working electrode.

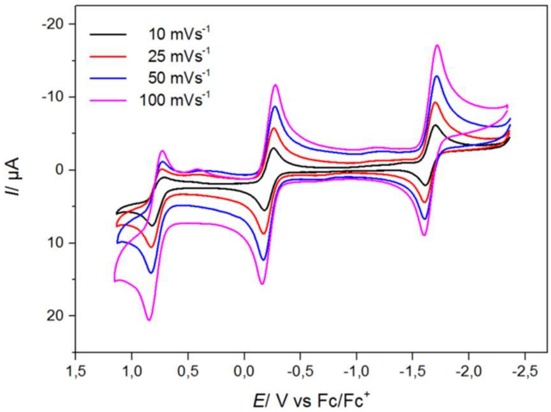

Figure S17. Cyclic voltammogram of **1** measured in 0.1 M Bu_4NPF_6/CH_2Cl_2 at room temperature with glassy carbon working electrode. Comparison of scan rates.

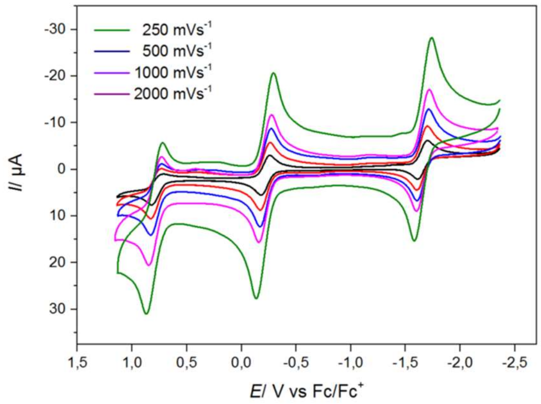

Figure S18. Cyclic voltammogram of **1** measured in 0.1 M Bu_4NPF_6/CH_2Cl_2 at room temperature with glassy carbon working electrode. Comparison of scan rates.

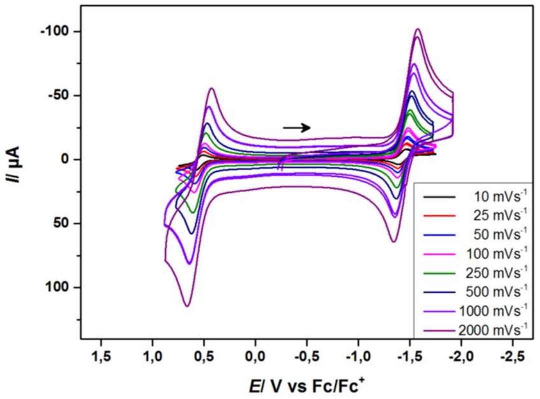

Figure S19. Cyclic voltammogram of **3** measured in 0.1 M Bu_4NPF_6/CH_2Cl_2 at room temperature with glassy carbon working electrode. Comparison of scan rates.

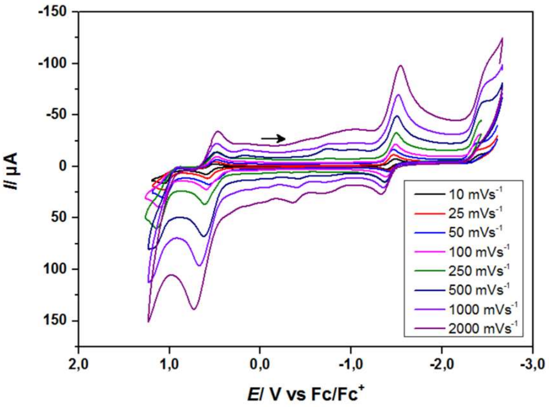

Figure S20. Cyclic voltammogram of **3** measured in 0.1 M Bu_4NPF_6/CH_2Cl_2 at room temperature with glassy carbon working electrode. Comparison of scan rates.

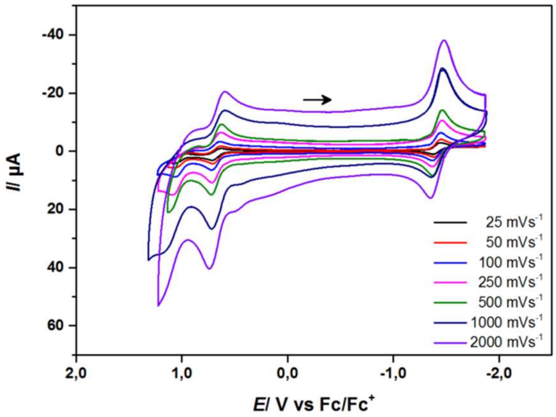

Figure S21. Cyclic voltammogram of **4** measured in 0.1 M Bu_4NPF_6/CH_2Cl_2 at room temperature with glassy carbon working electrode. Comparison of scan rates.

7 DFT calculations

7.1 Stereochemistry of 1 and 5

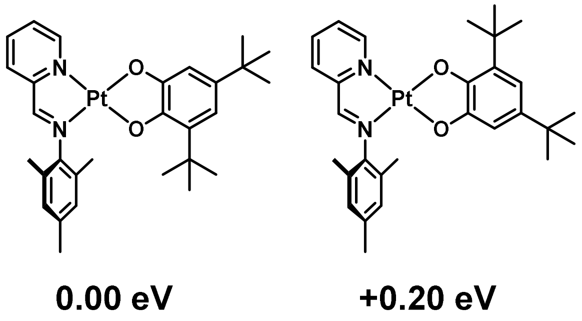

Figure S22. Relative energy differences of isomers for **1** (B3LYP/def2-TZVP//BP86/def2-TZVP).

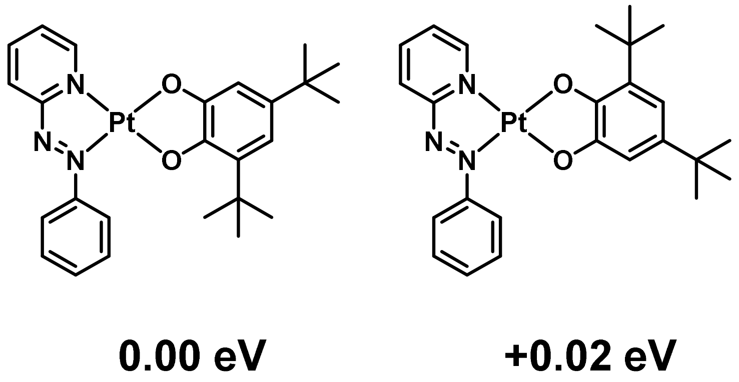

Figure S23. Relative energy differences of isomers for (pap)Pt(Q_{tBu}) taken from the literature.[6]

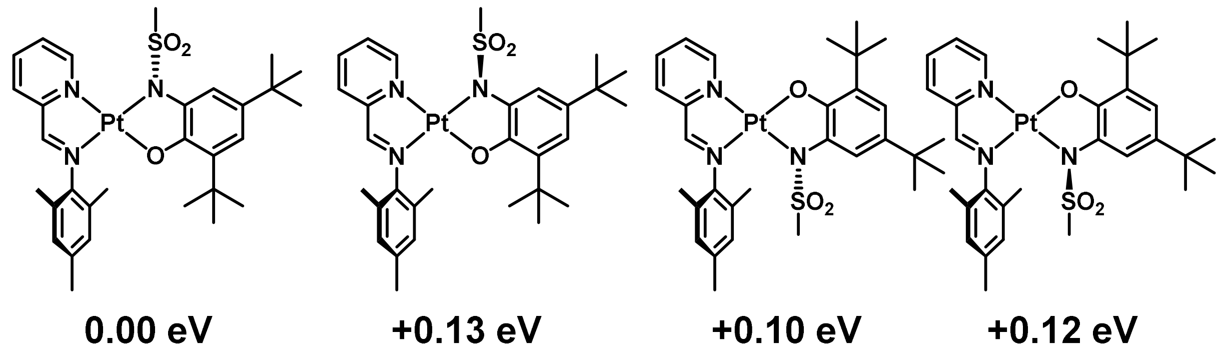

Figure S24. Relative energy differences of isomers for **5** (B3LYP/def2-TZVP//BP86/def2-TZVP).

[6] N. Deibel, D. Schweinfurth, J. Fiedler, S. Zalis, B. Sarkar, *Dalton Trans.* **2011**, *40*, 9925.

7.2 Rearrangement of 4

Figure S25. All studied isomers of **4⁺** with the relative energies (B3LYP/def2-TZVP//BP86/def2-TZVP for themochemistry).

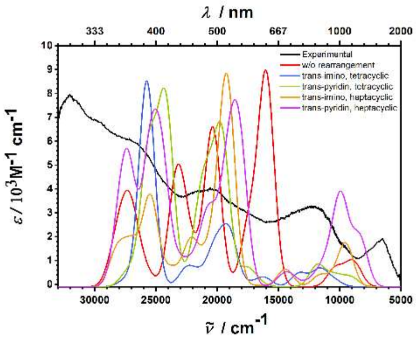

Figure S26. Comparison of calculated UV/Vis/NIR spectra with the experimental spectrum for all isomers at B3LYP/def2-TZVP.

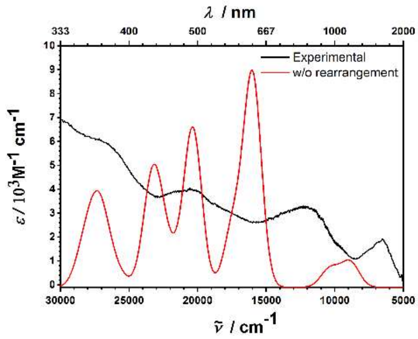

Figure S27. Comparison of calculated UV/Vis/NIR spectra with the experimental spectrum for **4⁺** without rearrangement at B3LYP/def2-TZVP.

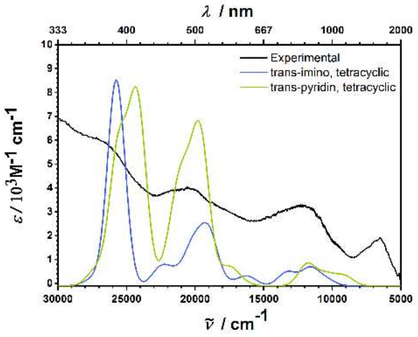

Figure S28. Comparison of calculated UV/Vis/NIR spectra with the experimental spectrum for the tetracyclic isomers of **4⁺** at B3LYP/def2-TZVP.

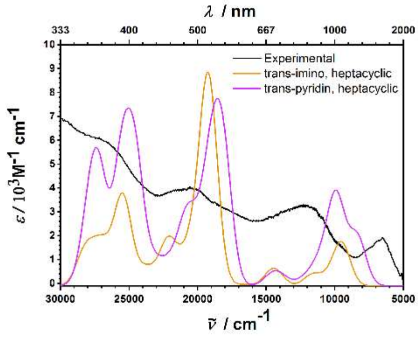

Figure S29. Comparison of calculated UV/Vis/NIR spectra with the experimental spectrum for the heptacyclic isomers of **4⁺** at B3LYP/def2-TZVP.

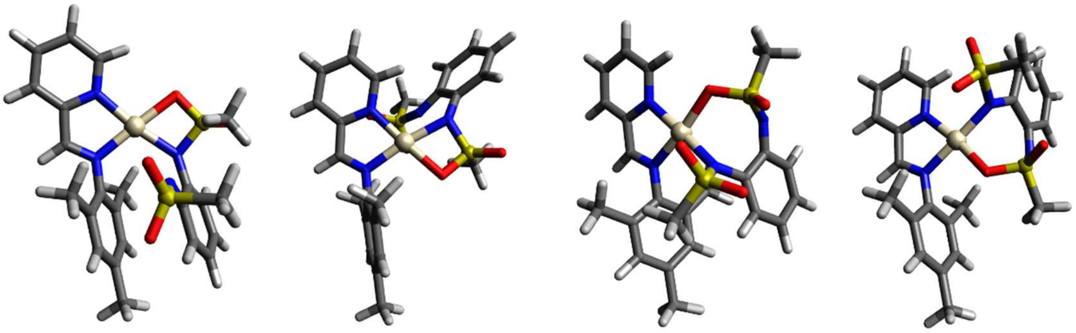

Figure S30. Calculated geometries for different isomers of **4⁺** at BP86/def2-TZVP. From left to right: Tetracyclic trans-imino **4⁺**, tetracyclic trans-pyridin **4⁺**, heptacyclic trans-imino **4⁺** and heptacyclic trans-pyridin **4⁺**.

8 EPR-Spectroelectrochemistry and Spin Densities

Table S1: Overview of EPR parameters used for simulation.

Compound	$g_{ox.}$	A_{ox}(Pt)	$\rho_{MullikenOx}$	g_{red}	A_{red}(Pt)	$\rho_{MullikenRed}$
**1**	1.997	2.73	3.7	1.994	8.95	7.9
**2**	2.001	3.67	4.8	2.000	3.13	7.1
**3**	2.011	11.90	9.4	1.991	9.04	5.8
**4**	2.012	4.59	10.7	1.991	8.49	5.6
**5**	2.001	5.63	5.3	1.992	9.36 mT	5.8

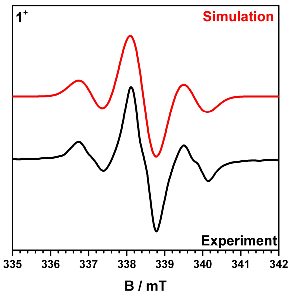

Figure S31. EPR spectrum of electrochemically generated $\mathbf{1}^{+}$ in 0.1 NBu_4PF_6 / CH_2Cl_2.

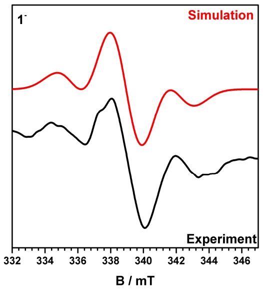

Figure S32. EPR spectrum of electrochemically generated $\mathbf{1}^{-}$ in 0.1 NBu_4PF_6 / CH_2Cl_2.

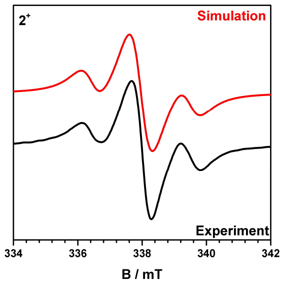

Figure S33. EPR spectrum of electrochemically generated $\mathbf{2}^{+}$ in 0.1 NBu_4PF_6 / CH_2Cl_2.

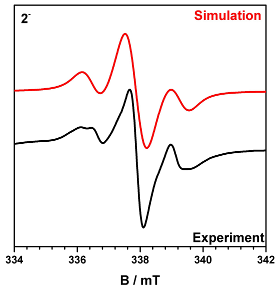

Figure S34. EPR spectrum of electrochemically generated $\mathbf{2}^{-}$ in 0.1 NBu_4PF_6 / CH_2Cl_2.

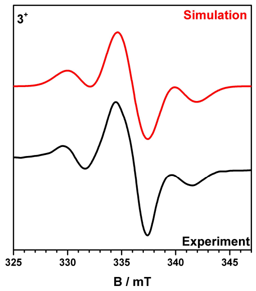

Figure S35. EPR spectrum of electrochemically generated $\mathbf{3}^{+}$ in 0.1 NBu_4PF_6 / CH_2Cl_2.

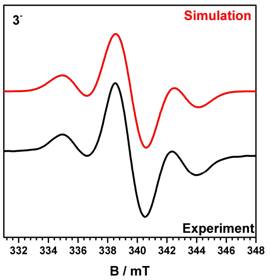

Figure S36. EPR spectrum of electrochemically generated $\mathbf{3}^{-}$ in 0.1 NBu_4PF_6 / CH_2Cl_2.

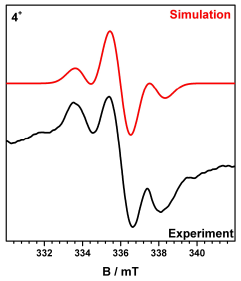

Figure S37. EPR spectrum of electrochemically generated $\mathbf{4}^{+}$ in 0.1 NBu_4PF_6 / CH_2Cl_2.

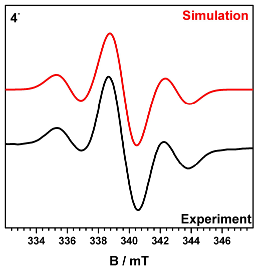

Figure S38. EPR spectrum of electrochemically generated $\mathbf{4}^{-}$ in 0.1 NBu_4PF_6 / CH_2Cl_2.

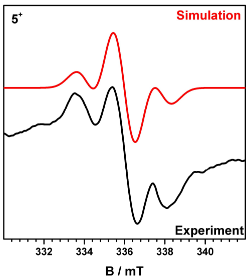

Figure S39. EPR spectrum of electrochemically generated **5**$^+$ in 0.1 NBu_4PF_6 / CH_2Cl_2.

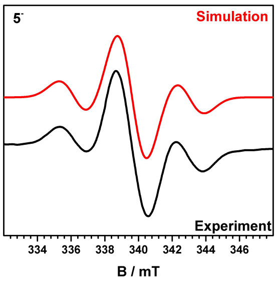

Figure S40. EPR spectrum of electrochemically generated **5**$^–$ in 0.1 NBu_4PF_6 / CH_2Cl_2.

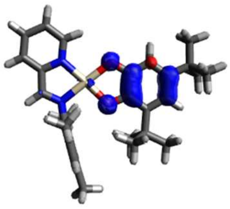

Figure S41. Spin population analysis plot for **1**$^+$.

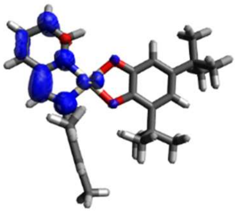

Figure S42. Spin population analysis plot for **1**$^–$.

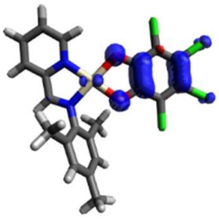

Figure S43. Spin population analysis plot for **2**$^+$.

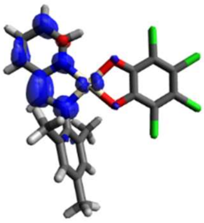

Figure S44. Spin population analysis plot for **2**$^–$.

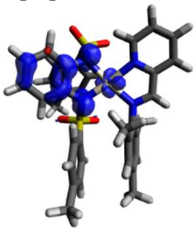

Figure S45. Spin population analysis plot for **3**$^+$.

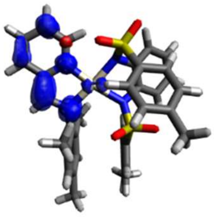

Figure S46. Spin population analysis plot for **3**$^–$.

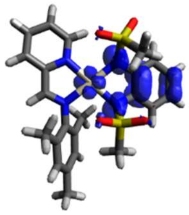

Figure S47. Spin population analysis plot for **4**$^+$.

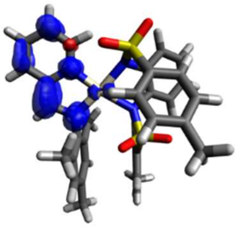

Figure S48. Spin population analysis plot for **4**$^–$.

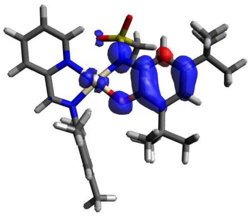

Figure S49. Spin population analysis plot for **5**$^+$.

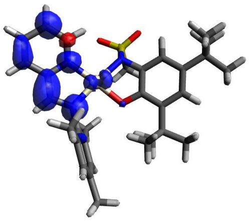

Figure S50. Spin population analysis plot for **5**$^–$.

9 UV-Vis-NIR-Spectroelectrochemistry

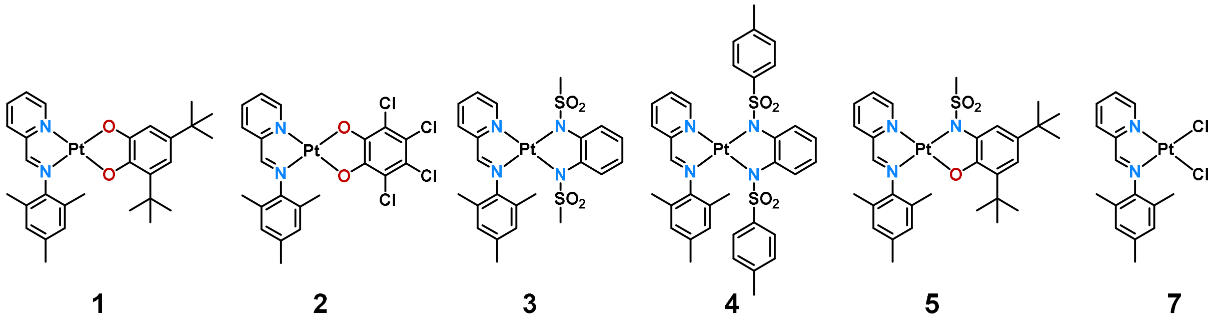

1 **2** **3** **4** **5** **7**

Table S2: (UV-)Vis-NIR data for compounds for **1**, **2**, **3**, **4**, **5** and **7** in the respective oxidation states from OTTLE-spectroelectrochemistry in DCM with 0.1 NBu_4PF_6 measured with agold or platinum mesh working electrode.

	**λ / nm (e / 10^3 M^{-1} • cm^{-1})**
**1**	306 (13.0), 388 (2.6) sh, 552 (2.2), 717 (8.7)
**1$^+$**	303 (10.6), 372 (4.9), 396 (4.3), 470 (9.6), 536 (5.9), 614 (3.2), 979 (0.2)
**1^{2+}**	303 (12.2), 348 (9.0), 375 (8.2), 443 (7.5), 559 (2.7)
**1$^-$**	313 (15.1), 360 (8.4), 450 (5.1), 470 (5.1), 613 (4.7), 857 (0.6) sh
**2**	301 (1.2), 312 (1.0), 366 (0.35) sh, 580 (0.7)
**2$^+$**	326 (0.9), 373 (0.6) sh, 482 (0.9), 504 (0.9), 632 (0.4), 965 (0.03)
**2$^-$**	297 (1.4), 353 (1.0) sh, 441 (0.8), 467 (0.83), 514 (0.6), 578 (0.4) sh, 827 (0.07) br
**3**	310 (3.6), 375 (2.2) sh, 537 (1.8), 889 (0.1)
**3$^+$**	309 (6.2), 368 (4.3) sh, 708 (4.5) sh, 907 (5.9)
**3^{+reox}**	313 (6.0), 381 (3.3), 454 (2.5), 900 (1.3), 1531 (1.6)
**3$^-$**	312 (6.0), 348 (4.9) sh, 438 (4.3) 464 (3.9), 532 (1.2), 569 (0.8), 807 (0.3)
**4**	323 (6.2), 374 (4.6) sh, 530 (2.9)
**4$^+$**	312 (8.0), 367 (6.1) sh, 486 (4.0), 822 (3.2), 1537(1.9)
**4^{2+}**	308 (12.5), 359 (8.6), 508 (6.8), 829 (15.3)
**4^{+reox}**	313 (12.6), 374 (6.7) sh, 452 (5.2), 833 (3.5), 1544 (3.4)
**4$^-$**	299 (16.0), 351 (11.2), 434 (11.2), 459 (10.2), 530 (2.7), 560 (2.0), 788 (1.2) br
**5**	301 (9.9), 338 (5.5) sh, 373 (3.3), 489 (1.6), 645 (4.1), 1010 (0.03) br
**5$^+$**	305 (10.6), 371 (4.3), 482 (5.4), 541 (6.5), 606 (5.0), 1026 (0.7)
**5^{2+}**	302 (11.9), 426 (7.6), 537 (5.1), 1027 (0.1)
**5$^-$**	303 (12.6), 353 (7.4), 440 (6.1), 466 (5.6), 557 (2.7) sh, 833 (1.1) br
**7**	305 (0.13), 347 (0.14) 446 (0.17)
**7$^-$**	308 (0.37), 356 (0.29), 439 (0.36), 530 (0.04), 569 (0.03), 734 (0.02) br, 823 (0.01) br
Obtained from spectroelectrochemical measurements using an OTTLE-cell with a gold working electrode in CH_2CL_2/0.1 M NBu_4PF_6 at 295K.	

9.1 Spectra for 1

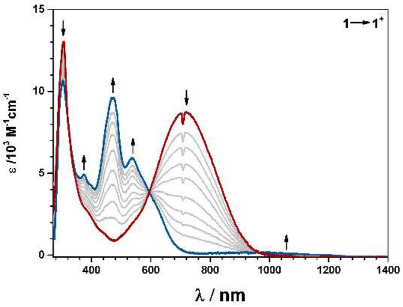

Figure S51. Changes during UV/Vis/NIR-SEC for **1** to **1**$^+$ in 0.1 M NBu_4PF_6 / CH_2Cl_2.

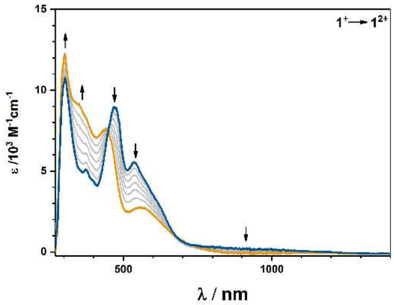

Figure S52. Changes during UV/Vis/NIR-SEC for **1**$^+$ to **1**$^{2+}$ in 0.1 M NBu_4PF_6 / CH_2Cl_2.

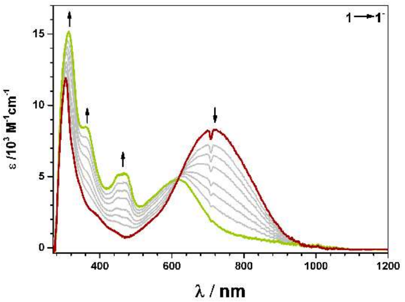

Figure S53. Changes during UV/Vis/NIR-SEC for **1** to **1**$^-$ in 0.1 M NBu_4PF_6 / CH_2Cl_2.

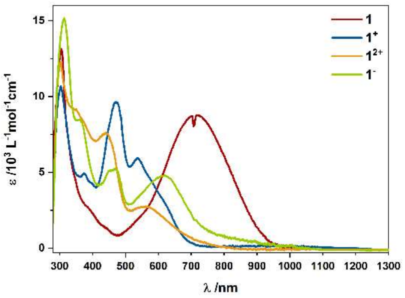

Figure S54. Comparison of UV/Vis/NIR spectra for **1**, **1**$^+$, **1**$^{2+}$ and **1**$^-$.

9.2 Spectra for 2

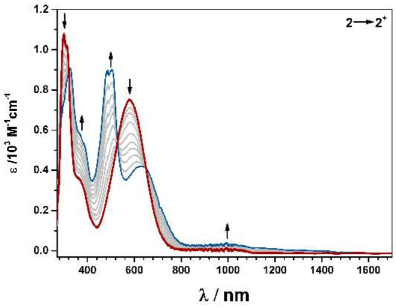

Figure S55. Changes during UV/Vis/NIR-SEC for **2** to **2**$^+$ in 0.1 M NBu_4PF_6 / CH_2Cl_2.

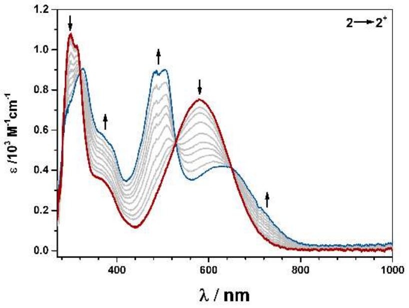

Figure S56. Zoom of the changes during UV/Vis/NIR-SEC for **2** to **2**$^+$ in 0.1 M NBu_4PF_6 / CH_2Cl_2.

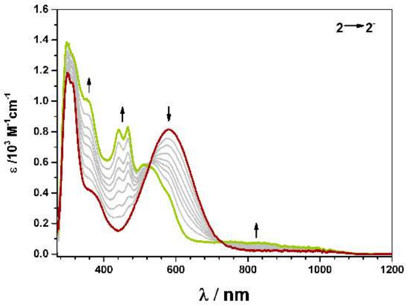

Figure S57 Changes during UV/Vis/NIR-SEC for **2** to **2**$^-$ in 0.1 M NBu_4PF_6 / CH_2Cl_2.

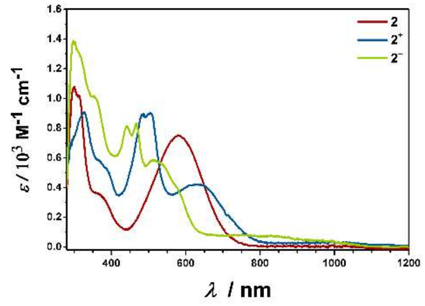

Figure S58. Comparison of UV/Vis/NIR spectra for **2**, **2**$^+$ and **2**$^-$.

9.3 Spectra for 3

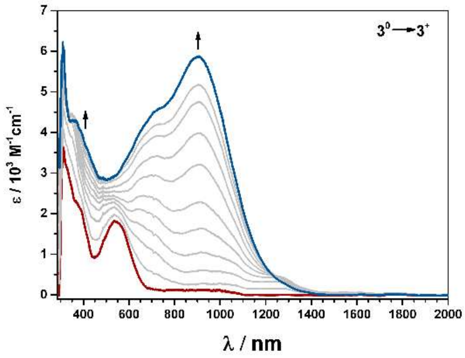

Figure S59. Changes during UV/Vis/NIR-SEC for **3** to $\mathbf{3}^+$ in 0.1 M NBu_4PF_6 / CH_2Cl_2.

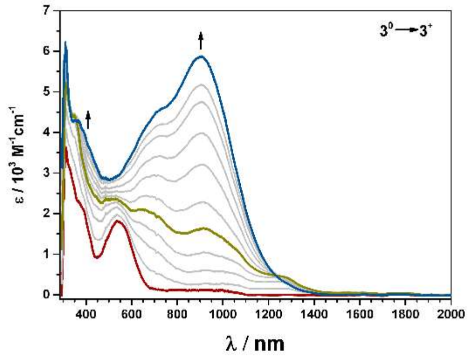

Figure S60. Changes during UV/Vis/NIR-SEC for **3** to $\mathbf{3}^+$ in 0.1 M NBu_4PF_6 / CH_2Cl_2.

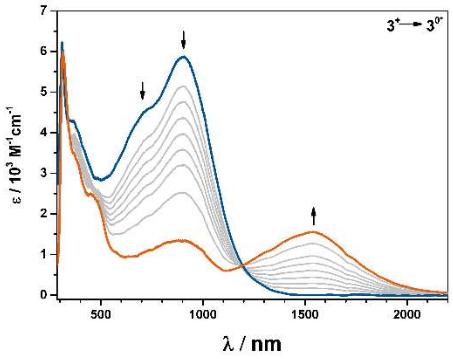

Figure S61. Changes during UV/Vis/NIR-SEC for $\mathbf{3}^+$ to **3** in 0.1 M NBu_4PF_6 / CH_2Cl_2.

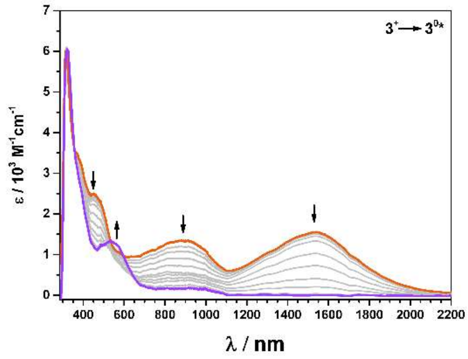

Figure S62 Changes during UV/Vis/NIR-SEC for $\mathbf{3}^+$ to **3** in 0.1 M NBu_4PF_6 / CH_2Cl_2. Part 2.

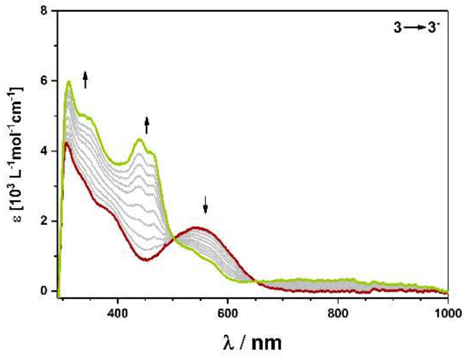

Figure S63 Changes during UV/Vis/NIR-SEC for **3** to $\mathbf{3}^-$ in 0.1 M NBu_4PF_6 / CH_2Cl_2.

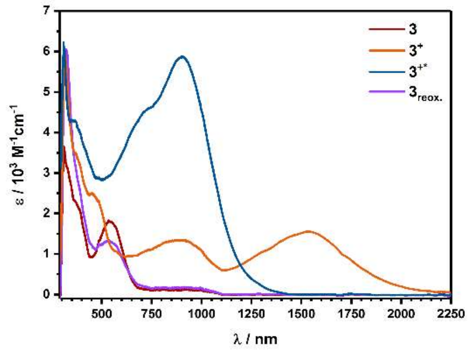

Figure S64. Comparison of UV/Vis/NIR spectra for **3**, $\mathbf{3}^+$, $\mathbf{3}^{+*}$ and $\mathbf{3}_{reox}$.

9.4 Spectra for 4

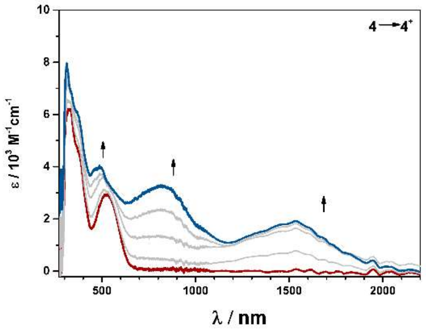

Figure S65. Changes during UV/Vis/NIR-SEC for **4** to **4^{+}** in 0.1 M NBu_4PF_6 / CH_2Cl_2.

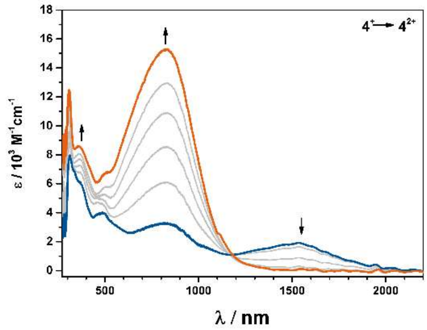

Figure S66. Changes during UV/Vis/NIR-SEC for **4^{+}** to **4^{2+}** in 0.1 M NBu_4PF_6 / CH_2Cl_2.

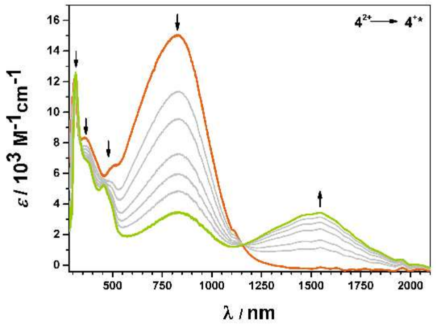

Figure S67. Changes during UV/Vis/NIR-SEC for **4^{2+}** to **4^{+}*** in 0.1 M NBu_4PF_6 / CH_2Cl_2.

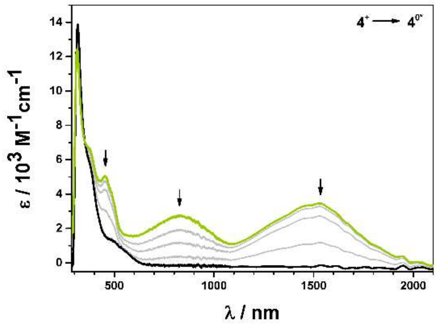

Figure S68. Changes during UV/Vis/NIR-SEC for **4^{+}** to **4*** in 0.1 M NBu_4PF_6 / CH_2Cl_2.

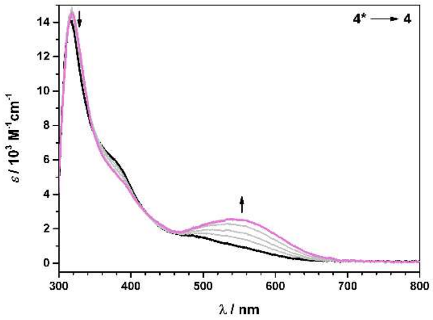

Figure S69. Changes during UV/Vis/NIR-SEC for **4*** to **4** in 0.1 M NBu_4PF_6 / CH_2Cl_2.

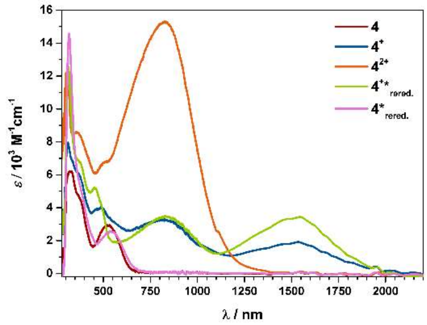

Figure S71. Comparison of UV/Vis/NIR spectra for **4**, **4^{+}**, **4^{2+}**, **4^{+}***and **4_{reox}**.

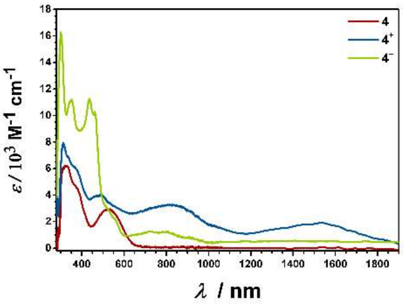

Figure S70. Comparison of UV/Vis/NIR spectra for **4**, **4^{+}** and **4^{-}**.

9.5 Spectra for 5

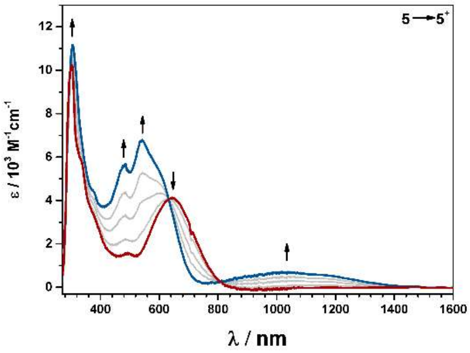

Figure S72. Changes during UV/Vis/NIR-SEC for **5** to **5⁺** in 0.1 M NBu_4PF_6 / CH_2Cl_2.

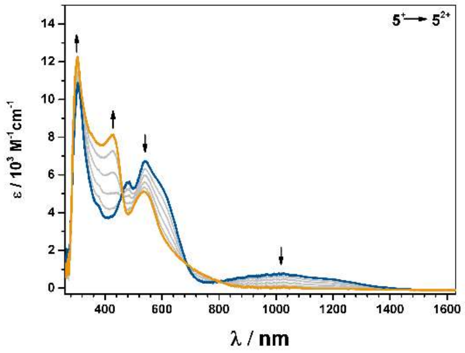

Figure S73. Changes during UV/Vis/NIR-SEC for **5⁺** to **5²⁺** in 0.1 M NBu_4PF_6 / CH_2Cl_2.

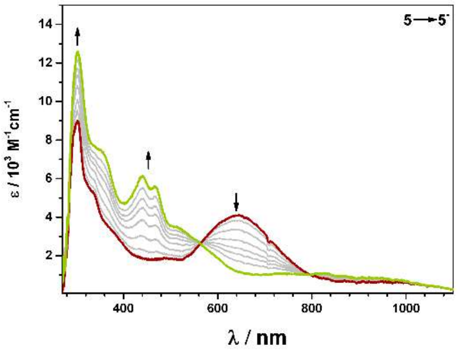

Figure S74. Changes during UV/Vis/NIR-SEC for **5** to **5⁻** in 0.1 M NBu_4PF_6 / CH_2Cl_2.

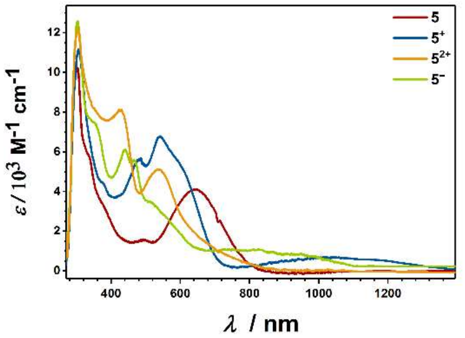

Figure S75. Comparison of UV/Vis/NIR spectra for **5**, **5⁺**, **5²⁺** and **5⁻**.

9.6 Spectra for 7

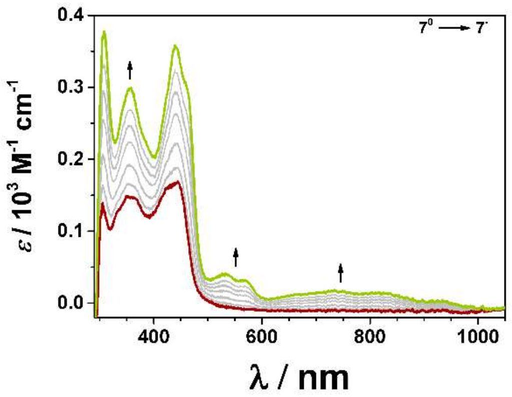

Figure S76. Changes during UV/Vis/NIR-SEC for **7** to **7⁻** in 0.1 M NBu_4PF_6 / CH_2Cl_2.

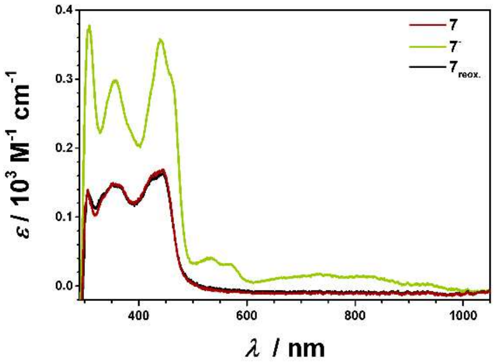

Figure S77. Comparison of UV/Vis/NIR spectra for **7**, **7⁻**.and **7reox.**

9.7 Comparison of UV/Vis/NIR spectra

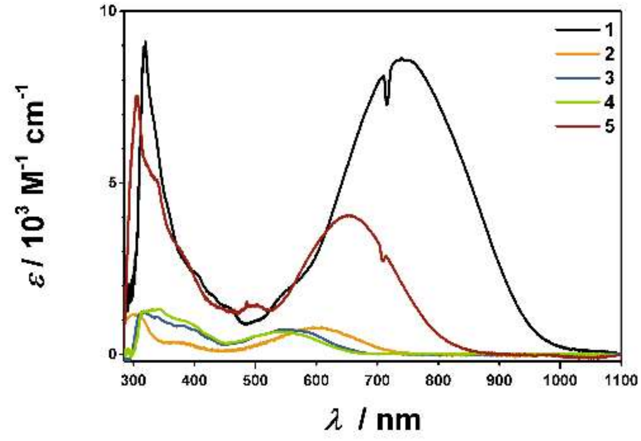

Figure S78. Comparison of UV/Vis/NIR spectra for **1**, **2**, **3**, **4** and **5**.

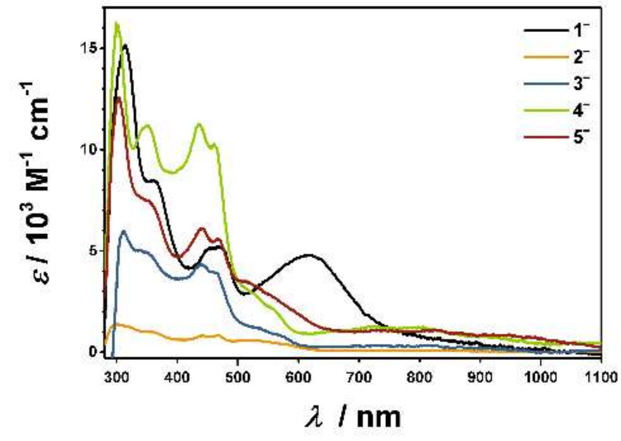

Figure S79. Comparison of UV/Vis/NIR spectra for **1**$^{-}$, **2**$^{-}$, **3**$^{-}$, **4**$^{-}$ and **5**$^{-}$.

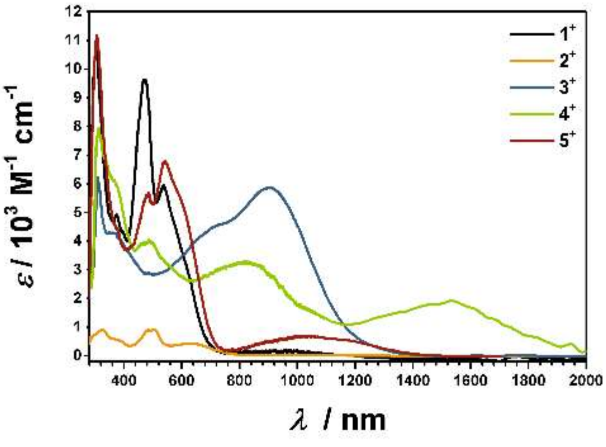

Figure S80. Comparison of UV/Vis/NIR spectra for **1**$^{+}$, **2**$^{+}$, **3**$^{+}$, **4**$^{+}$ and **5**$^{+}$.

10 Chemical oxidation of 3

In order to further characterize the product of the linkage isomerism for compound 3 chemical oxidation with $AgPF_6$ and $[NO]BF_4$ in DCM was attempted. Although a color change was observed, the spectrum does not match with the spectroelectrochemically generated spectrum, which indicates decomposition of the complex (see below). This precluded further analysis.

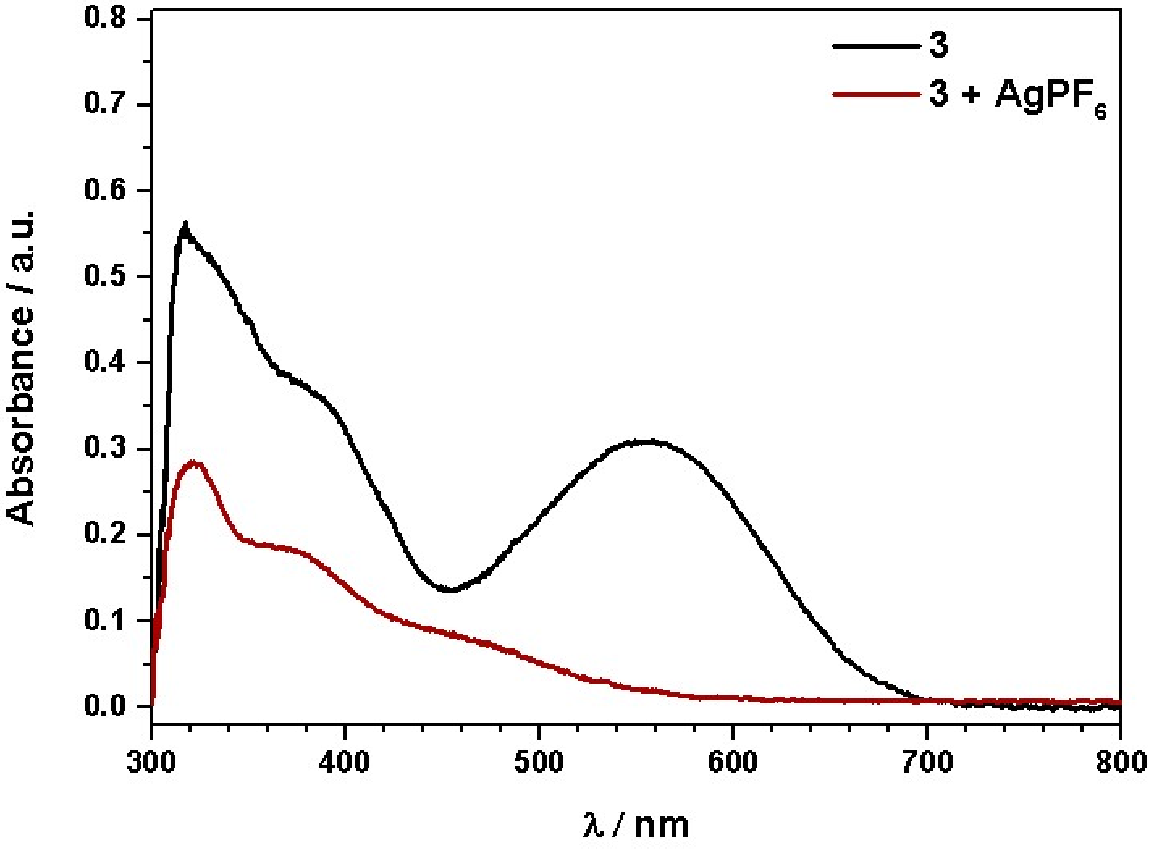

Figure S81. Generic spectrum of the oxidation of complex **3** with a chemical oxidation agent ($AgPF_6$ in this case).

11 (TD)DFT

11.1 DFT Calculation for 1 (singlet state)

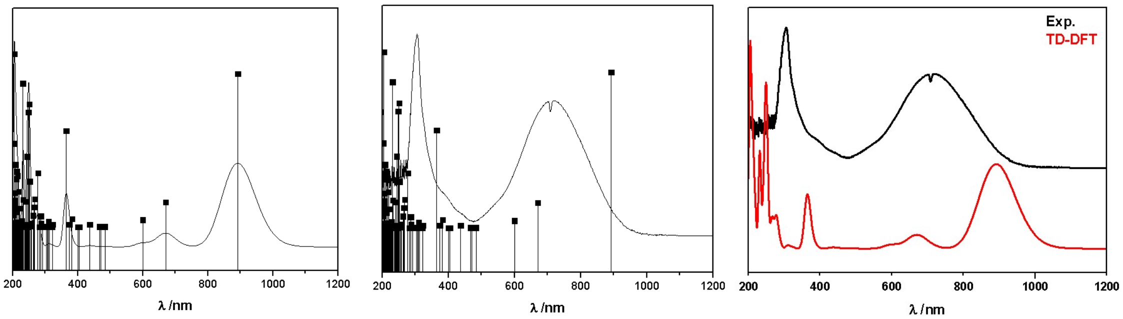

Figure S82: Calculated TD-DFT spectrum with discrete transitions (left), experimental spectrum with discrete calculated transitions (middle) and experimental (black) and calculated TD-DFT spectrum (red) (left).

Table S3: TD-DFT transitions for **1**.

State	Difference density (iso value 0.001)	Transition homo lumo	Calculated Transition energy	Oscillator strength	Experimental transition energy	Molar absorption coefficient 10^3
1	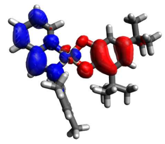	HOMO -> LUMO (0.91)	891.7	0.242	717	8.7
2	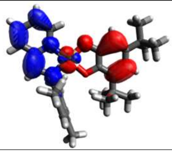	HOMO-1 -> LUMO (0.89)	670.3	0.039		
3	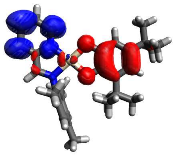	HOMO -> LUMO+1 (0.97)	599.7	0.011		
11	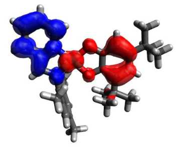	HOMO-6 -> LUMO (0.37)<br>HOMO-1 -> LUMO+1 (0.49)	364.4	0.151	306	13.0

Table S4: Selected molecular orbitals for **1**.

HOMO-6	HOMO-1	HOMO	LUMO	LUMO+1
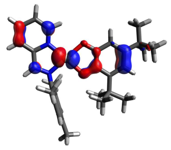	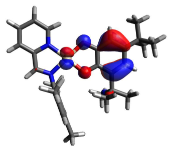	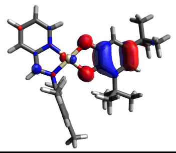	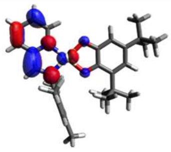	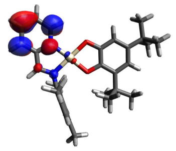

Table S5: Selected molecular orbital energies for **1**.

Orbital No.	HOMO/LUMO	Energy (Eh)	Energy (eV)
152	HOMO-6	-0.251501	-6.8437
153	HOMO-5	-0.247783	-6.7425
154	HOMO-4	-0.245415	-6.6781
155	HOMO-3	-0.242405	-6.5962
156	HOMO-2	-0.235934	-6.4201
157	HOMO-1	-0.207120	-5.6360
158	HOMO	-0.172852	-4.7035
159	LUMO	-0.104633	-2.8472
160	LUMO+1	-0.053943	-1.4679

11.2 DFT Calculation for 1^{+} (doublet state)

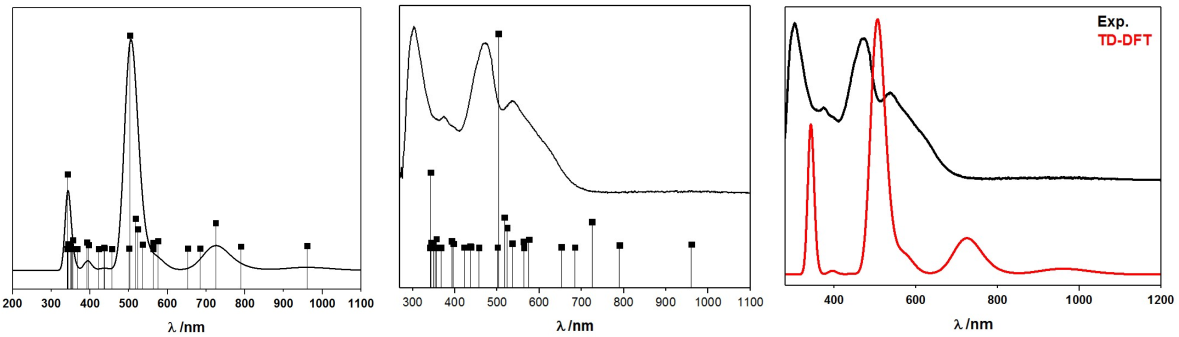

Figure S83. Calculated TD-DFT spectrum with discrete transitions (left), experimental spectrum with discrete calculated transitions (middle) and experimental (black) and calculated TD-DFT spectrum (red) (left).

Table S6. TD-DFT transitions for $\mathbf{1}^{+}$.

State	Difference density (iso value 0.002)	Transition homo lumo	Calculated Transition energy	Oscillator strength	Experimental transition energy	Molar absorption coefficient 10^3
1	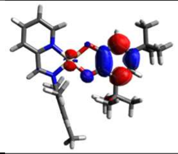	HOMOβ -> LUMOβ (0.96)	960.4	0.0028	979	0.2
3	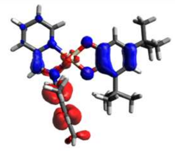	HOMOα -> LUMOα (0.76)	724.7	0.0222	614	3.2
8	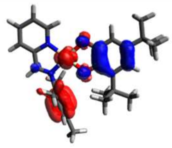	HOMOβ-4 -> LUMOβ (0.85)	503.5	0.1835	470	9.6
30	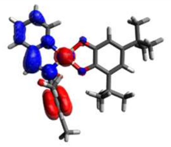	HOMOα-6 -> LUMOα (0.33) HOMOβ-4 -> LUMOβ+1 (0.35)	342.6	0.0644	372	4.9

Table S7: Selected molecular orbitals for **1**$^+$.

HOMOβ-4	HOMOβ	LUMOβ	LUMOβ+1
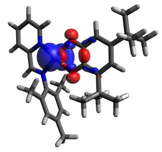	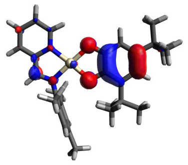	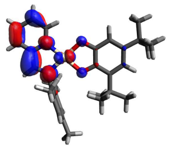	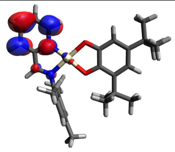
HOMOα-6	HOMOα	LUMOα	LUMOα+1
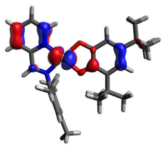	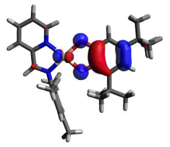	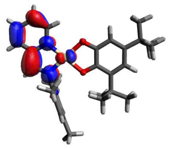	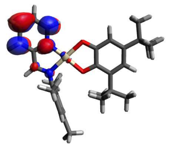

Table S8: Orbital energies for **1**$^+$ (doublet state).

Spin up Orbitals alpha				Spin down orbitals beta			
Orbital No.	Orbital	Energy (Eh)	Energy (eV)	Orbital No.	Orbital	Energy (Eh)	Energy (eV)
122	HOMOα-6	-0.290423	-7.9028	122	HOMOβ-5	-0.283488	-7.7141
123	HOMOα-5	-0.285208	-7.7609	123	HOMOβ-4	-0.281119	-7.6496
124	HOMOα-4	-0.279044	-7.5932	124	HOMOβ-3	-0.277848	-7.5606
125	HOMOα-3	-0.257707	-7.0126	125	HOMOβ-2	-0.257693	-7.0122
126	HOMOα-2	-0.255012	-6.9392	126	HOMOβ-1	-0.253002	-6.8845
127	HOMOα-1	-0.253028	-6.8852	127	HOMOβ	-0.247416	-6.7325
128	HOMOα	-0.226226	-6.1559	128	LUMOβ	-0.158243	-4.3060
129	LUMOα	-0.128280	-3.4907	129	LUMOβ+1	-0.123923	-3.3721
130	LUMOα+1	-0.076349	-2.0776	130	LUMOβ+2	-0.075434	-2.0527

11.3 DFT Calculation for 1^{2+} (singlet state)

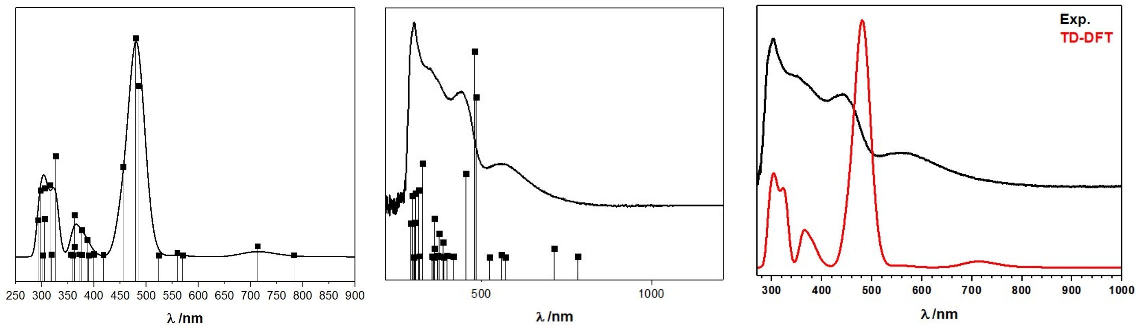

Figure S84: Calculated TD-DFT spectrum with discrete transitions (left), experimental spectrum with discrete calculated transitions (middle) and experimental (black) and calculated TD-DFT spectrum (red) (left).

Table S9: TD-DFT transitions for **1**$^{2+}$.

State	Difference density (iso value 0.001)	Transition homo lumo	Calc. Transition energy	Oscillator strength	Exp. transition energy	Molar absorption coefficient 10^3
3	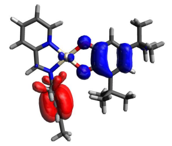	HOMO-2-> LUMO (0.98)	713.3	0.0070	559	2.7
7	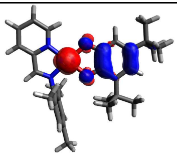	HOMO-5 -> LUMO (0.26) HOMO-4 -> LUMO (0.18) HOMO -> LUMO+1 (0.53)	484.7	0.1269	443	7.5
8	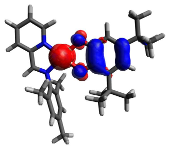	HOMO-5 -> LUMO (0.30) HOMO-4 -> LUMO (0.21) HOMO -> LUMO+1 (0.45)	479.8	0.1630		
10	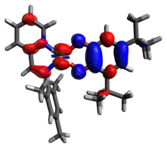	HOMO-7 -> LUMO (0.92)	455.9	0.0665		
18	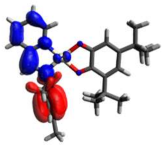	HOMO-3 -> LUMO+2 (0.27) HOMO -> LUMO+2 (0.52)	326.8	0.0745	348	9.0

Table S10: Selected molecular orbitals for **1**$^{2+}$.

HOMO-7	HOMO-5	HOMO-4	HOMO-3	HOMO-2
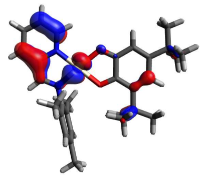	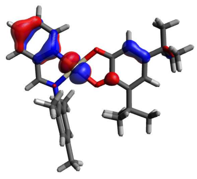	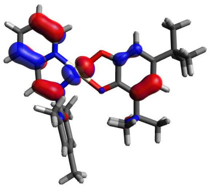	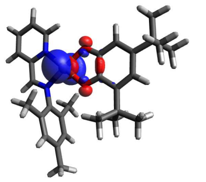	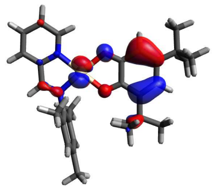
HOMO-1	HOMO	LUMO	LUMO+1	LUMO+2
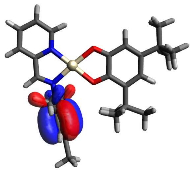	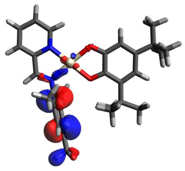	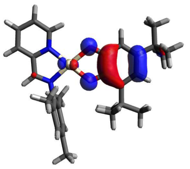	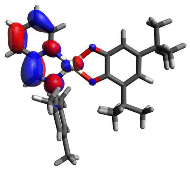	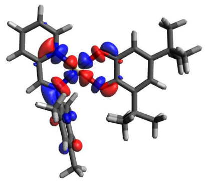

Table S11: Selected molecular orbital energies for **1**$^{2+}$.

Orbital No.	HOMO/LUMO	Energy (Eh)	Energy (eV)
120	HOMO-7	-0.344581	-9.3765
121	HOMO-6	-0.336137	-9.1468
122	HOMO-5	-0.322479	-8.7751
123	HOMO-4	-0.318159	-8.6575
124	HOMO-3	-0.312989	-8.5169
125	HOMO-2	-0.298221	-8.1150
126	HOMO-1	-0.274080	-7.4581
127	HOMO	-0.271414	-7.3855
128	LUMO	-0.203496	-5.5374
129	LUMO+1	-0.144458	-3.9309
130	LUMO+2	-0.102234	-2.7819

11.4 DFT Calculation for 1^{-} (doublet state)

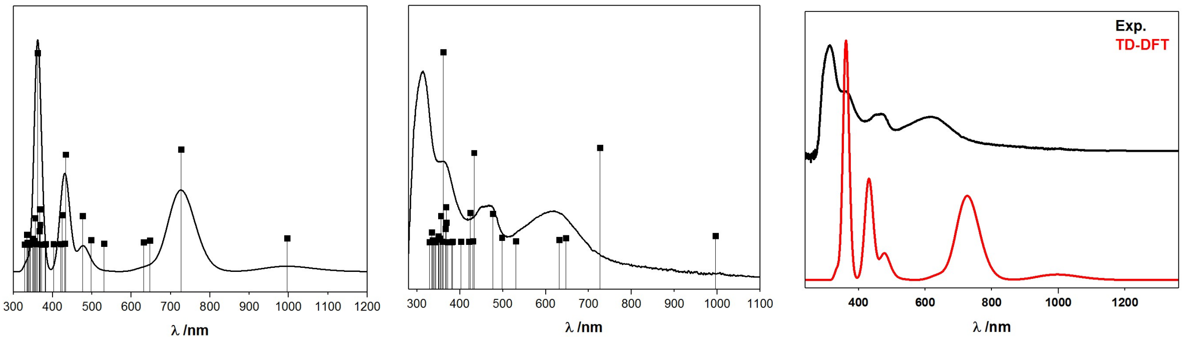

Figure S85: Calculated TD-DFT spectrum with discrete transitions (left), experimental spectrum with discrete calculated transitions (middle) and experimental (black) and calculated TD-DFT spectrum (red) (left).

Table S12: TD-DFT transitions for **1**$^{-}$.

State	Difference density (iso value 0.001)	Transition homo lumo	Calc. Trans. energy	Oscillator strength	Exp. transition energy	Molar absorption coefficient 10^3
1	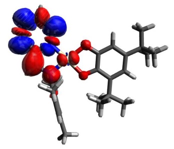	HOMOα -> LUMOα (0.96)	996.1	0.0045	857	0.6
2	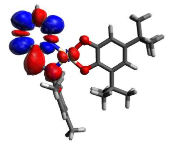	HOMOβ -> LUMOβ (0.95)	726.5	0.0689	613	4.7
6	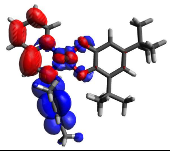	HOMOα -> LUMOα+4 (0.73)	433.0	0.0652	450	5.1
9	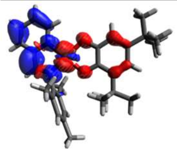	HOMOα-1 -> LUMOα (0.39) HOMOβ -> LUMOβ+1 (0.41)	476.6	0.0205		

Table S13: Selected molecular orbitals for **1**⁻.

HOMOα-1	HOMOα	LUMOα	LUMOα+4
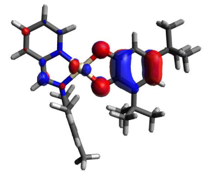	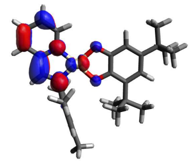	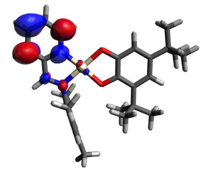	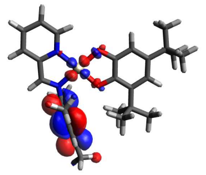
HOMOβ-1	HOMOβ	LUMOβ	LUMOβ+1
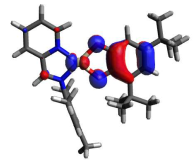	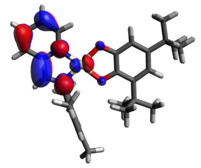	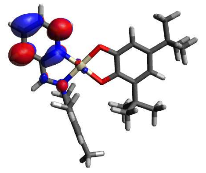	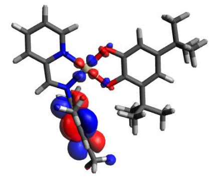

Table S14: Orbital energies for **1**⁻ (doublet state)

Spin up Orbitals alpha				Spin down orbitals beta			
Orbital No.	Orbital	Energy (Eh)	Energy (eV)	Orbital No.	Orbital	Energy (Eh)	Energy (eV)
128	HOMOα-1	-0.151169	-4.1135	128	HOMOβ	-0.145718	-3.9652
129	HOMOα	-0.107807	-2.9336	129	LUMOβ	-0.043699	-1.1891
130	LUMOα	-0.016324	-0.4442	130	LUMOβ+1	-0.010445	-0.2842
131	LUMOα+1	0.003012	0.0820	131	LUMOβ+2	0.003273	0.0891
132	LUMOα+2	0.010002	0.2722	132	LUMOβ+3	0.010368	0.2821
133	LUMOα+3	0.033068	0.8998	133	LUMOβ+4	0.033618	0.9148
134	LUMOα+4	0.035898	0.9768	134	LUMOβ+5	0.041025	1.1163

11.5 DFT Calculation for 2 (singlet state)

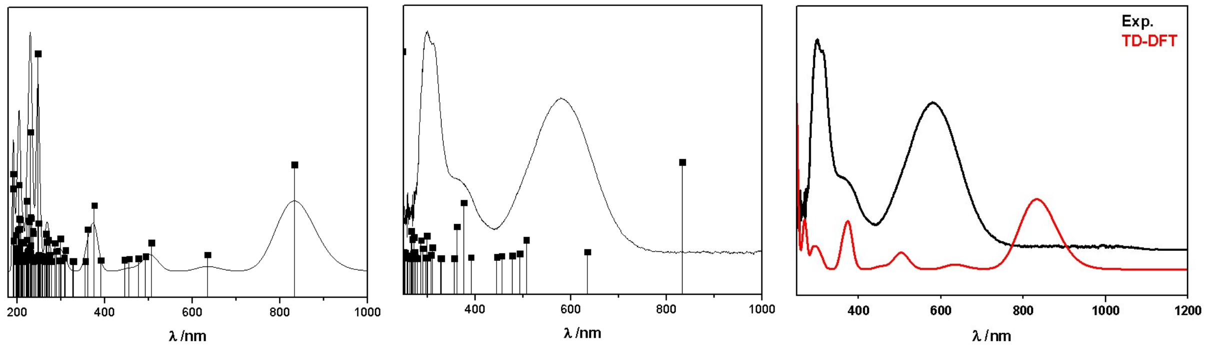

Figure S86: Calculated TD-DFT spectrum with discrete transitions (left), experimental spectrum with discrete calculated transitions (middle) and experimental (black) and calculated TD-DFT spectrum (red) (left).

Table S15: TD-DFT transitions for **2**.

State	Difference density (iso value 0.001)	Transition homo lumo	Calc. Trans. energy	Oscillator strength	Exp. transition energy	Molar absorption coefficient 10^3
12	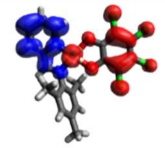	HOMO-1 -> LUMO+1 (0.78)	376.4	0.1065	366	0.35
9	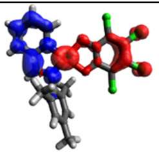	HOMO-6 -> LUMO (0.60) HOMO-1 -> LUMO+1 (0.17)	361.9	0.0614		
1	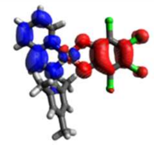	HOMO -> LUMO (0.94)	833.1	0.1845	580	0.7
2	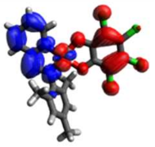	HOMO-1 -> LUMO (0.90)	634.9	0.0124		
3	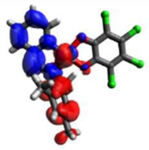	HOMO-2 -> LUMO (0.83)	506.8	0.0355		

Table S16: Selected molecular orbitals for **2**.

HOMO-6	HOMO-2	HOMO-1	HOMO	LUMO	LUMO+1
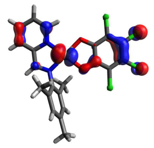	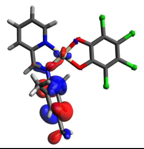	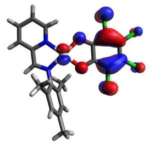	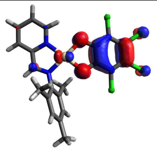	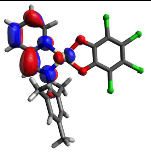	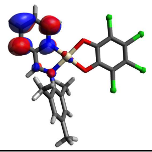

Table S17: Selected molecular orbital energies for **2**.

Orbital No.	HOMO/LUMO	Energy (Eh)	Energy (eV)
152	HOMO-6	-0.261825	-7.1246
153	HOMO-5	-0.260193	-7.0802
154	HOMO-4	-0.255982	-6.9656
155	HOMO-3	-0.245102	-6.6696
156	HOMO-2	-0.240593	-6.5469
157	HOMO-1	-0.220963	-6.0127
158	HOMO	-0.194125	-5.2824
159	LUMO	-0.114131	-3.1057
160	LUMO+1	-0.060219	-1.6386
161	LUMO+2	-0.028819	-0.7842
162	LUMO+3	-0.017012	-0.4629

11.6 DFT Calculation for 3 (singlet state)

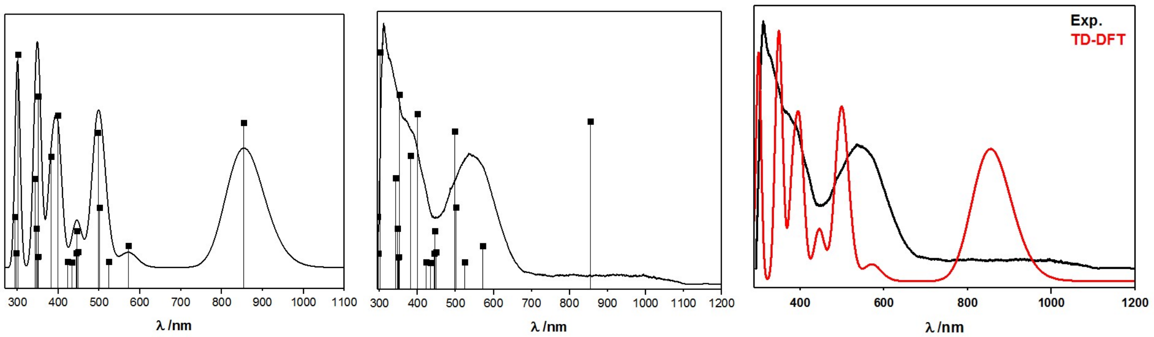

Figure S87: Calculated TD-DFT spectrum with discrete transitions (left), experimental spectrum with discrete calculated transitions (middle) and experimental (black) and calculated TD-DFT spectrum (red) (left).

Table S18: TD-DFT transitions for **3**.

State	Difference density (iso value 0.001)	Transition homo lumo	Calc. Transition energy	Oscillator strength	Exp. transition energy	Molar absorption coefficient 10^3
1	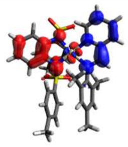	HOMO -> LUMO (0.98)	854.4	0.0391	889	0.1
4	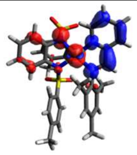	HOMO-4 -> LUMO (0.17)<br>HOMO-2 -> LUMO (0.58)	497.2	0.0363	526	2.9
10	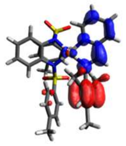	HOMO-8 -> LUMO (0.78)	399.0	0.0412	323	6.2
17	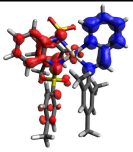	HOMO-1 -> LUMO+1 (0.83)	351.5	0.0464		

Table S19: Selected molecular orbitals for **3**.

HOMO-8	HOMO-4	HOMO-2	HOMO-1	HOMO	LUMO	LUMO+1
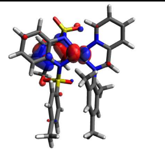	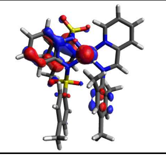	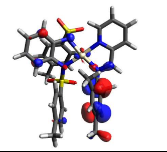	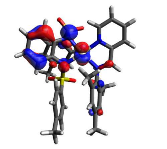	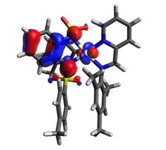	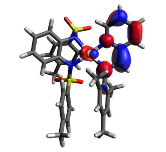	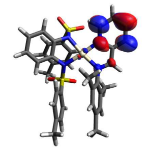

Table S20: Selected molecular orbital energies for **3**.

Orbital No.	HOMO/LUMO	Energy (Eh)	Energy (eV)
168	HOMO-8	-0.257236	-6.9997
169	HOMO-7	-0.255666	-6.9570
170	HOMO-6	-0.251286	-6.8378
171	HOMO-5	-0.248471	-6.7612
172	HOMO-4	-0.240822	-6.5531
173	HOMO-3	-0.238216	-6.4822
174	HOMO-2	-0.233325	-6.3491
175	HOMO-1	-0.225171	-6.1272
176	HOMO	-0.199102	-5.4178
177	LUMO	-0.108052	-2.9402
178	LUMO+1	-0.054930	-1.4947

11.7 DFT Calculation for 3^+ (singlet state)

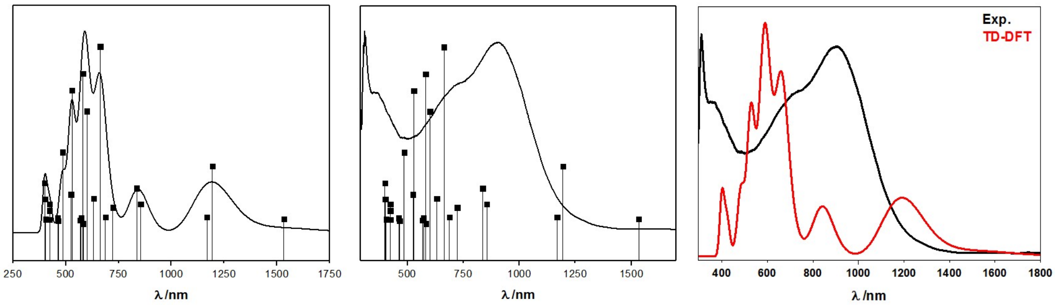

Figure S88: Calculated TD-DFT spectrum with discrete transitions (left), experimental spectrum with discrete calculated transitions (middle) and experimental (black) and calculated TD-DFT spectrum (red) (left).

Table S21: TD-DFT transitions for **3⁺**.

State	Difference density (iso value 0.001)	Transition homo lumo	Calc. Trans. energy	Oscillator strength	Exp. transition energy	Molar absorption coefficient 10^3
1	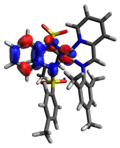	HOMOβ-2 -> LUMOβ (0.21) HOMOβ-1 -> LUMOβ (0.50) HOMOβ -> LUMOβ (0.23)	1194.8	0.0110	907	5.9
4	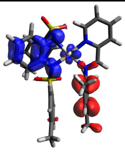	HOMOβ-4 -> LUMOβ (0.31) HOMOβ-3 -> LUMOβ (0.28)	836.0	0.0068		
8	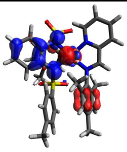	HOMOβ-7 -> LUMOβ (0.60)	663.7	0.0337	708	4.5
9	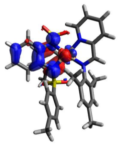	HOMOβ-9 -> LUMOβ (0.62)	581.9	0.0285		

Table S22: Selected molecular orbitals for **3⁺**.

HOMOβ-7	HOMOβ-4	HOMOβ-3	HOMOβ-2	HOMOβ-1	HOMOβ	LUMOβ
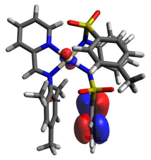	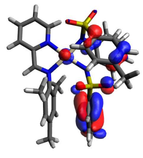	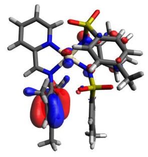	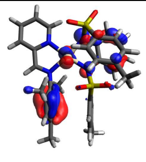	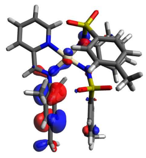	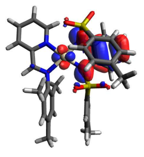	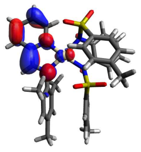

Table S23: Orbital energies for **3⁺** (doublet state).

Spin up Orbitals alpha				Spin down orbitals beta			
Orbital No.	Orbital	Energy (Eh)	Energy (eV)	Orbital No.	Orbital	Energy (Eh)	Energy (eV)
166	HOMOα-10	-0.296403	-8.0655	166	HOMOβ-9	-0.290114	-7.8944
167	HOMOα-9	-0.291405	-7.9295	167	HOMOβ-8	-0.289512	-7.8780
168	HOMOα-8	-0.285149	-7.7593	168	HOMOβ-7	-0.282012	-7.6739
169	HOMOα-7	-0.280730	-7.6390	169	HOMOβ-6	-0.280338	-7.6284
170	HOMOα-6	-0.276753	-7.5308	170	HOMOβ-5	-0.275900	-7.5076
171	HOMOα-5	-0.274589	-7.4719	171	HOMOβ-4	-0.271347	-7.3837
172	HOMOα-4	-0.268267	-7.2999	172	HOMOβ-3	-0.266914	-7.2631
173	HOMOα-3	-0.263018	-7.1571	173	HOMOβ-2	-0.253091	-6.8869
174	HOMOα-2	-0.252778	-6.8784	174	HOMOβ-1	-0.252092	-6.8598
175	HOMOα-1	-0.248940	-6.7740	175	HOMOβ	-0.248090	-6.7509
176	HOMOα	-0.237530	-6.4635	176	LUMOβ	-0.176751	-4.8096
177	LUMOα	-0.128258	-3.4901	177	LUMOβ+1	-0.126490	-3.4420
178	LUMOα+1	-0.073874	-2.0102	178	LUMOβ+2	-0.073221	-1.9924

11.8 DFT Calculation for 4 (singlet state)

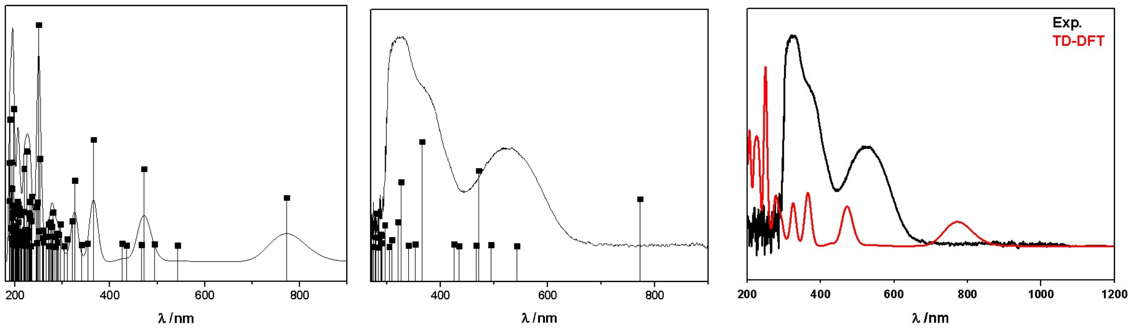

Figure S89: Calculated TD-DFT spectrum with discrete transitions (left), experimental spectrum with discrete calculated transitions (middle) and experimental (black) and calculated TD-DFT spectrum (red) (left).

Table S24: TD-DFT transitions for **4**.

State	Difference density (iso value 0.002)	Transition homo lumo	Calc. Trans. energy	Oscillator strength	Experimental transition energy	Molar absorption coefficient 10^3
1	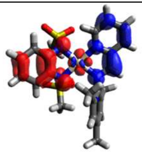	HOMO-> LUMO (0.96)	772.4	0.049	526	2.9
4	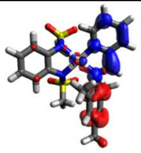	HOMO-2-> LUMO (0.87)	471.7	0.078		
8	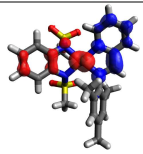	HOMO-6 -> LUMO (0.67)	365.4	0.107	377	4.5
12	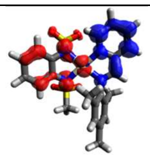	HOMO-1-> LUMO+1 (0.52)	326.7	0.066	323	6.2

Table S25: Selected molecular orbitals for **4**.

HOMO-6	HOMO-1	HOMO	LUMO	LUMO+1
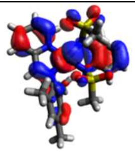	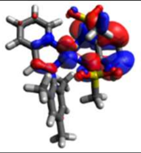	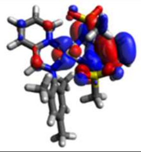	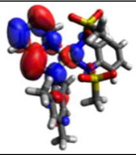	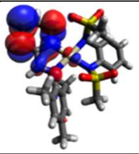

Table S26: Selected molecular orbital energies for **4**.

Orbital No.	HOMO/LUMO	Energy (Eh)	Energy (eV)
159	HOMO-7	-0.279308	-7.6004
160	HOMO-6	-0.261448	-7.1144
161	HOMO-5	-0.256815	-6.9883
162	HOMO-4	-0.251817	-6.8523
163	HOMO-3	-0.247305	-6.7295
164	HOMO-2	-0.242521	-6.5993
165	HOMO-1	-0.230940	-6.2842
166	HOMO	-0.203597	-5.5402
167	LUMO	-0.111339	-3.0297
168	LUMO+1	-0.057187	-1.5561
169	LUMO+2	-0.031262	-0.8507
170	LUMO+3	-0.018452	-0.5021

11.9 DFT Calculation for 4^+ (doublet state)

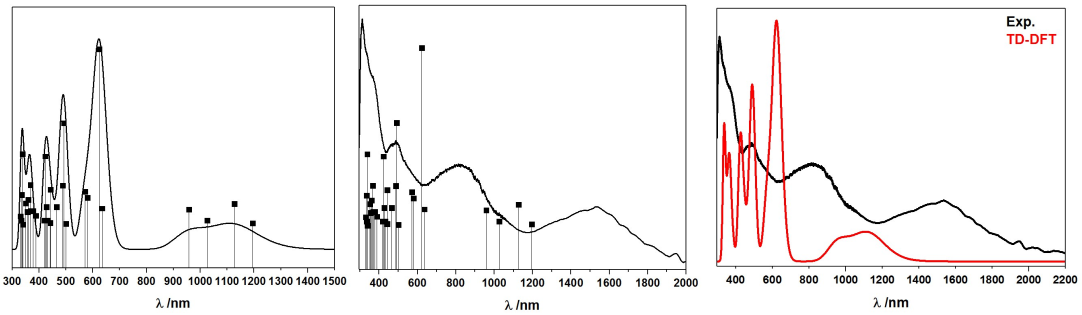

Figure S90: Calculated TD-DFT spectrum with discrete transitions (left), experimental spectrum with discrete calculated transitions (middle) and experimental (black) and calculated TD-DFT spectrum (red) (left).

Table S27: TD-DFT transitions for **4⁺**.

State	Difference density (iso value 0.002)	Transition homo lumo	Calculated Transition energy	Oscillator strength	Experimental transition energy	Molar absorption coefficient 10^3
1	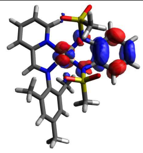	HOMOβ-1 -> LUMOβ (0.63) HOMOβ -> LUMOβ (0.33)	1127.0	0.0082	1537	1.9
2	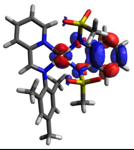	HOMOβ-3 -> LUMOβ (0.35) HOMOβ -> LUMOβ (0.36)	1026.4	0.0016		
3	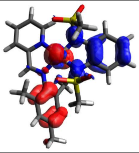	HOMOβ-3 -> LUMOβ (0.43) HOMOβ -> LUMOβ (0.26)	958.7	0.0060		
6	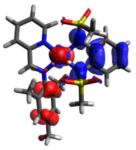	HOMOβ-4 -> LUMOβ (0.86)	623.7	0.0697	822	3.2
12	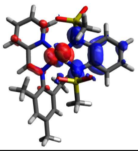	HOMOβ-1 -> LUMOβ+1 (0.33) HOMOβ -> LUMOβ+1 (0.36)	492.0	0.0403	486	4.0

Table S28: Selected molecular orbitals for **4**⁺.

HOMOβ-4	HOMOβ-3	HOMOβ-2	HOMOβ-1	HOMOβ	LUMOβ	LUMOβ+1
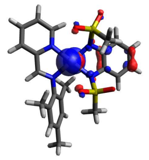	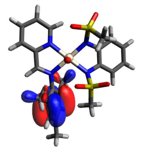	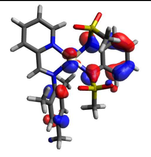	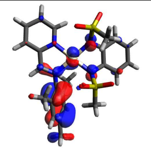	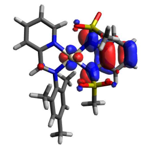	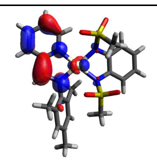	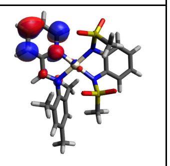

Table S29: Orbital energies for **4**⁺ (doublet state).

Spin up Orbitals alpha				Spin down orbitals beta			
Orbital No.	Orbital	Energy (Eh)	Energy (eV)	Orbital No.	Orbital	Energy (Eh)	Energy (eV)
131	HOMOα-5	-0.294303	-8.0084	131	HOMOβ-4	-0.289316	-7.8727
132	HOMOα-4	-0.281172	-7.6511	132	HOMOβ-3	-0.277399	-7.5484
133	HOMOα-3	-0.272038	-7.4025	133	HOMOβ-2	-0.263330	-7.1656
134	HOMOα-2	-0.263316	-7.1652	134	HOMOβ-1	-0.260026	-7.0757
135	HOMOα-1	-0.257384	-7.0038	135	HOMOβ	-0.256498	-6.9797
136	HOMOα	-0.242598	-6.6014	136	LUMOβ	-0.179941	-4.8964
137	LUMOα	-0.129708	-3.5295	137	LUMOβ+1	-0.127484	-3.4690
138	LUMOα+1	-0.074747	-2.0340	138	LUMOβ+2	-0.074062	-2.0153

11.10 DFT Calculation for 5 (singlet state)

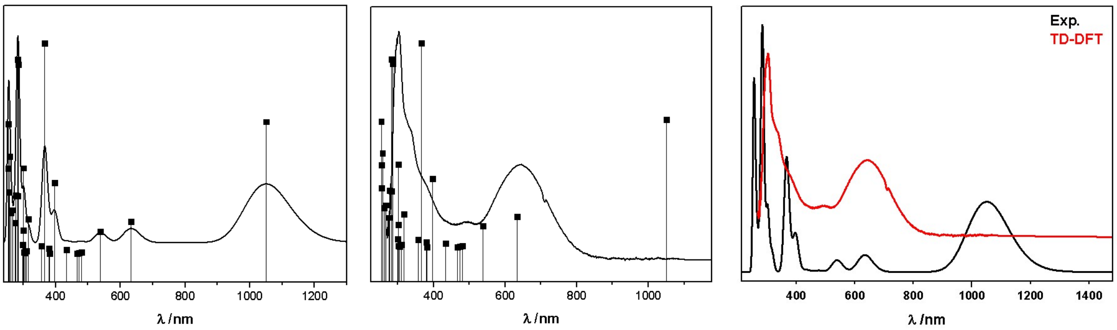

Figure S91: Calculated TD-DFT spectrum with discrete transitions (left), experimental spectrum with discrete calculated transitions (middle) and experimental (black) and calculated TD-DFT spectrum (red) (left).

Table S30: TD-DFT transitions for **5**.

State	Difference density (iso value 0.002)	Transition homo lumo	Calculated Transition energy	Oscillator strength	Experimental transition energy	Molar absorption coefficient 10^3
1	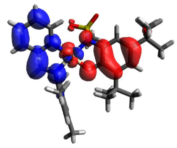	HOMO -> LUMO (0.98)	1050.6	0.0514	1010	0.3
2	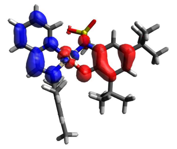	HOMO-1 -> LUMO (0.93)	633.6	0.0124	655	4.0
5	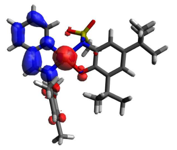	HOMO -> LUMO+1 (0.94)	538.5	0.0086		
10	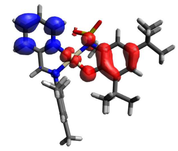	HOMO-7 -> LUMO (0.11) HOMO-6 -> LUMO (0.72)	366.5	0.0821	338	5.0
11	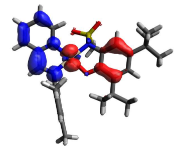	HOMO-1 -> LUMO+1 (0.90)	396.7	0.0277		

Table S31: Selected molecular orbitals for **5**.

HOMO-7	HOMO-6	HOMO-1	HOMO	LUMO	LUMO+1
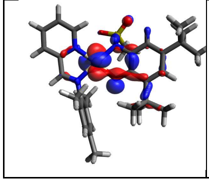	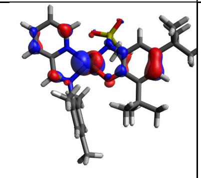	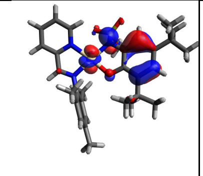	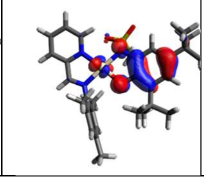	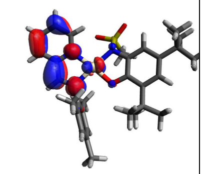	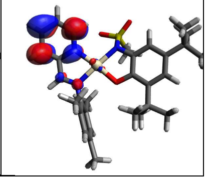

Table S32: Selected molecular orbital energies for **5**.

Orbital No.	HOMO/LUMO	Energy (Eh)	Energy (eV)
141	HOMO-7	-0.265806	-7.2329
142	HOMO-6	-0.254614	-6.9284
143	HOMO-5	-0.248949	-6.7743
144	HOMO-4	-0.245359	-6.6766
145	HOMO-3	-0.242741	-6.6053
146	HOMO-2	-0.238361	-6.4861
147	HOMO-1	-0.212813	-5.7909
148	HOMO	-0.182641	-4.9699
149	LUMO	-0.100268	-2.7284
150	LUMO+1	-0.050339	-1.3698

12 Coordinates of optimized structures

1

Pt	4.72064755470248	1.23568249072540	2.75514358082042
O	4.52381133432520	-0.48437004799324	1.76172612482843
O	2.83646998044393	0.89074553806841	3.23453235161652
N	4.90551538316884	2.92431301711768	3.73778385511453
N	6.61030986033302	1.68357623123430	2.33593211178973
C	2.36826480646558	-0.25494415453823	2.69919905741798
C	7.45005858805565	0.95035662226930	1.56982908263118
H	7.03110484327207	0.03436215670688	1.15503916772544
C	7.06771455839297	2.86400741036026	2.90075570446888
C	1.58690237412364	-2.67642533756016	1.49234131532009
C	8.75726476508718	1.34766490498155	1.33377608340018
H	9.39567722098995	0.72383516218957	0.71013996248662
C	8.38203785373321	3.30185505211552	2.68723330143035
H	8.70956044139383	4.23438948056089	3.14608490141475
C	2.88790161209838	-2.19978123444218	1.30639171628104
H	3.62001188992800	-2.73745014768942	0.70422334969439
C	0.06719443984219	0.09856803180468	3.76411525460612
C	1.04579180291998	-0.71171196581946	2.90520696013298
C	9.23556958309773	2.54206894583026	1.89951260596814
H	10.26036532533653	2.86657699263630	1.72263103666790
C	-0.10563344298081	-3.69413575339168	-0.06136079194847
H	0.18751469646428	-3.00904200044395	-0.87054530206879
H	-0.93545918408238	-3.23153698481167	0.49063959900782
H	-0.47654908283167	-4.62640221712790	-0.51482478895044
C	3.61982969537698	3.02244421253481	5.80844550515691
C	3.79626654036416	3.44273294428385	4.48112508530413
C	0.69862693597393	-1.91907702623469	2.28788413801553
H	-0.31306198855684	-2.29654132030425	2.42797118908572
C	2.84460909206269	4.23052363183545	3.81050357013971
C	1.09469507092177	-3.98758505046963	0.86443973009382
C	4.62001532279210	2.11707199313323	6.47278045393135
H	4.75133899479151	1.19275144228020	5.88839751387936
H	4.28884432076825	1.84896341431079	7.48336887706028
H	5.61156055948425	2.58790410977093	6.54861306588420
C	6.07490911117497	3.53014071985583	3.68285671322513
H	6.25269785835880	4.47055389552137	4.20409801314341
C	0.63234613822740	0.25010356982538	5.19447859383094
H	0.77088077246095	-0.73663537580483	5.66175991352152
H	-0.06935861514285	0.82921782368874	5.81459884764365
H	1.59407380381549	0.77603561656266	5.18326015060809
C	0.64988303784420	-4.95633999001318	1.98147120925993
H	0.28664920643375	-5.90066128044961	1.54713781479666
H	-0.16044701286035	-4.52746857357272	2.58711182575074
H	1.49048225501082	-5.18576605519378	2.65289613191745
C	-1.30822690188263	-0.57835711247663	3.86141989525407
H	-1.78185530968677	-0.68674740913146	2.87442707448513
H	-1.97121315057634	0.03809302678766	4.48617411515705
H	-1.24187080176195	-1.57455597366776	4.32340113375961
C	-0.12769747026029	1.50048578774417	3.14372209645613
H	0.82223527603615	2.04591537889623	3.10037291489200
H	-0.83331085853156	2.08510576911350	3.75424035563116
H	-0.53912292981095	1.41907076587842	2.12625927256619
C	3.02995700643502	4.60040922416941	2.36487695887626
H	3.12953479761056	3.69496269871467	1.74563984483258
H	3.94315538522009	5.19375551725267	2.20816819738240
H	2.17447722821410	5.18216619680470	2.00078399073960
C	2.45946236463157	3.43628836314674	6.47354195349131
H	2.30152499966888	3.10818776884146	7.50364854822889
C	2.18518502375955	-4.68020606451567	0.03358425362745
H	2.52499202461883	-4.04669958618865	-0.79884181997767
H	1.78613239548132	-5.61173954559204	-0.39376755990625
H	3.05978478383780	-4.93965731007590	0.64796009381069
C	3.27557255609829	-0.99482511535292	1.90733295480912
C	1.48890831122928	4.22513368894141	5.84722564287356
C	1.70391377763485	4.61710252743030	4.51925534413438
H	0.95017632643601	5.22054317888456	4.00753417426585
C	0.22132764292339	4.61052552073753	6.56388001888658
H	0.02541499761878	5.68972020140480	6.48185938647644
H	0.26890726006696	4.34769559855244	7.62864024757971
H	-0.64585256959269	4.09108122635668	6.12681636656339

1^{+}

Pt	4.74735142037232	1.21717096844750	2.77812468084936
O	4.52185484055851	-0.51958358056219	1.76886820267967
O	2.85474577578787	0.82614945008962	3.27715550805526
N	4.91926661690444	2.91796733565628	3.75550979310996
N	6.62790298355478	1.68687492162315	2.32800425471142
C	2.38791033311275	-0.27718452303875	2.74514188429072
C	7.45568260357328	0.95840915936942	1.55898829615057
H	7.04911163142156	0.03254054526800	1.15505707617709
C	7.06877775160386	2.87143298884679	2.87421315853634
C	1.61529747012479	-2.67611136741859	1.49872104887005
C	8.75986431474044	1.37822071668767	1.30154555493132
H	9.39901718279533	0.75949817364979	0.67405372333847
C	8.36245388215568	3.33381841083520	2.64611103168665
H	8.68065689122247	4.27518098977999	3.09196232006359
C	2.90882069648002	-2.22002513076815	1.29727889770927
H	3.62749784420332	-2.75503690398991	0.67964158406507
C	0.08632298058142	0.05593007761968	3.84637412216384
C	1.05240401090901	-0.73425616555982	2.96211571733491
C	9.22128591985541	2.57642674348309	1.84846447588025
H	10.23757240704094	2.91801646896747	1.65658274330260
C	-0.11718343236537	-3.62667891364210	-0.02912989487756
H	0.17618656540300	-2.94062276736950	-0.83649451176742
H	-0.93296454154763	-3.15995400572791	0.53920716623929
H	-0.50648754169280	-4.54995205187891	-0.48236424006687
C	3.52381416810022	2.93182835327691	5.75321151207664
C	3.79104121242045	3.43738821168193	4.47128075233178
C	0.71939504632743	-1.91587149764475	2.32827046207612
H	-0.28769610819166	-2.30553273653772	2.46071355879119
C	2.92065052071607	4.31482551840340	3.80124728921395
C	1.09436491942119	-3.96461895452596	0.87095270146022
C	4.45223561680839	1.94865971580865	6.40936162646181
H	4.56892414704608	1.04525460746471	5.78897802392374
H	4.06666860437457	1.64734398632405	7.39040147438272
H	5.45922484200658	2.36944308595867	6.54905929666371
C	6.06539293230646	3.53420831252085	3.67091559677608
H	6.23958024460333	4.48550761095937	4.17464612093873
C	0.65572055943698	0.14017146055697	5.28113874552162
H	0.79218291529450	-0.86497188864850	5.70600743449302
H	-0.04724082255784	0.69219761545100	5.92213732229285
H	1.61625796498712	0.66749522086771	5.29992919811187
C	0.64659733512433	-4.92506060746743	1.99716889128287
H	0.26633549301236	-5.85708847360726	1.55424874867847
H	-0.15451875653849	-4.49364871351285	2.61241910171948
H	1.49041634554913	-5.17395666186072	2.65643768402836
C	-1.29193596558009	-0.61803420995888	3.91040046742211
H	-1.76104943233073	-0.68523087885223	2.91772041344072
H	-1.95343082559575	-0.02104923473782	4.55351236777198
H	-1.23428530985648	-1.62977209333808	4.33808149253838
C	-0.09551084592330	1.48247375638063	3.27796382175423
H	0.84548645456200	2.04419633857653	3.28757013351396
H	-0.82478058195168	2.02904698701360	3.89372630000324
H	-0.47776353167496	1.44605190306685	2.24732540521960
C	3.20909968529236	4.77839212440029	2.39966715605159
H	3.36489507519562	3.92165079935164	1.72513542618993
H	4.12004603019657	5.39366557267464	2.35079007625749
H	2.37542808034048	5.37613368862119	2.01297106951264
C	2.34989633025898	3.35984359451889	6.38179059819570
H	2.12379949090993	2.97978704037691	7.38039212460445
C	2.15790307337178	-4.67057529145895	0.01881327273694
H	2.49833531434771	-4.03817460577086	-0.81380950345120
H	1.72992945986270	-5.58695051028132	-0.41055749372874
H	3.03341303742179	-4.95795475111364	0.61883823687668
C	3.30953712926247	-1.02213245600065	1.91164948380446
C	1.45270683240426	4.23443002478304	5.75811353969083
C	1.75893242671024	4.70400020256087	4.47349700481764
H	1.06426819690415	5.37841965662922	3.96759122686797
C	0.16464059089193	4.62816960361102	6.42981201419527
H	-0.04046384456879	5.70088523508410	6.30645568582350
H	0.18538014776808	4.39679844471479	7.50247146082278
H	-0.68385280526188	4.08358335331137	5.98595008440879

1^{2+}

Pt	4.72307507881660	1.21737438659611	2.73086710175228
O	4.45529056332962	-0.55500967005728	1.73420253978280
O	2.79350484851558	0.75661446274743	3.15469231845719
N	4.87659535840773	2.90747325086990	3.72983085486423
N	6.61699940014899	1.68074381632223	2.32150349736941
C	2.33425331636628	-0.28966454052779	2.68099377449080
C	7.44921388222131	0.97167210629501	1.56906521500079
H	7.07131757145845	0.05718962720669	1.13395937678450
C	7.03919836042534	2.83562579547645	2.89141289659902
C	1.66374959245418	-2.72122339865292	1.43864194823087
C	8.75630706630807	1.39069930035059	1.34996361416455
H	9.40591255875978	0.78816723088838	0.73145831725479
C	8.32118228581237	3.30489362685767	2.71345425644549
H	8.63256559684849	4.22969617046668	3.18005584475647
C	2.93925241865631	-2.28533459912575	1.23344768819618
H	3.65485702439904	-2.82988139387948	0.63502664457140
C	-0.01449587484070	0.01546951296400	3.72850095663929
C	0.98631513619493	-0.76235906599050	2.88734268714613
C	9.19932263835154	2.56557502487211	1.92476266699384
H	10.21238356290029	2.90905990528747	1.76677541470496
C	-0.07506008412768	-3.65919717970291	-0.04225447690386
H	0.20587491763628	-3.00209550702303	-0.86517675145378
H	-0.90232742018778	-3.19772354797592	0.49696075172014
H	-0.45876455281757	-4.58379232995214	-0.47386341803269
C	3.64706572298212	3.04749911268907	5.81615717173369
C	3.77387830796030	3.42396403743367	4.47822118346267
C	0.72573708406810	-1.93440062606156	2.26662742001942
H	-0.26281172549886	-2.35482135788259	2.37393055822933
C	2.84044898746078	4.22383369024491	3.81754083728079
C	1.13477836718660	-3.99940405638381	0.85295082935433
C	4.66400571425890	2.18062091071037	6.49930741217264
H	4.84103993352038	1.24262398349750	5.96810187656138
H	4.33673354065481	1.92224632255754	7.50382218296361
H	5.62835869596319	2.68145849564589	6.60361196555244
C	6.01275242615774	3.49016151827748	3.68089022553243
H	6.18938392370183	4.42421532563413	4.20492153188440
C	0.51003655815284	0.15921620310151	5.16676433339090
H	0.72079173125677	-0.81207062942646	5.61631941924555
H	-0.25552883791453	0.63812661413065	5.77755103723982
H	1.40200752266579	0.77957525235538	5.23849520636013
C	0.68887442839779	-4.91513217629233	2.01213800225737
H	0.30771425975127	-5.84625222913742	1.59244131784409
H	-0.11201206354969	-4.49362408758203	2.61967431483995
H	1.52132567955057	-5.16539102245149	2.66963248127362
C	-1.35352628837687	-0.72136925214108	3.77877165003726
H	-1.80948268783684	-0.83054874412240	2.79280948241546
H	-2.05436483633741	-0.14932495156196	4.38511533558279
H	-1.27219344040987	-1.70734842609367	4.24046605791397
C	-0.25063098354156	1.40142597503132	3.10592478393910
H	0.62971983784324	2.04149081924570	3.13930240758109
H	-1.03318329969109	1.91384315666417	3.66590932881078
H	-0.59041843237037	1.32489582554176	2.07221205990703
C	3.00283505287433	4.61004717321506	2.37676204144109
H	3.06823832198226	3.74295454850575	1.71504202518009
H	3.89772737638281	5.21205157119446	2.20939885083448
H	2.15713373810648	5.20520934089289	2.03981469526700
C	2.53705165263528	3.51924380714976	6.50243624147901
H	2.41744791862886	3.24976651990233	7.54514581815129
C	2.17766211851316	-4.73490973319262	0.01548219925920
H	2.51246957113286	-4.14726993985082	-0.84071105483200
H	1.74160410448964	-5.65001593485281	-0.38244780321140
H	3.04861222279248	-5.03048036796482	0.60247847415640
C	3.32451393688522	-1.07647116812730	1.83238269051962
C	1.58212128297177	4.32940062481472	5.89558872942388
C	1.75265416791742	4.66672589867784	4.55615493293811
H	1.01892443897594	5.30032127908727	4.07196785296364
C	0.41634936395026	4.85430231713436	6.67494491546470
H	0.66629211756234	5.81267590907781	7.13355205874955
H	0.12878108008007	4.17833309000107	7.47852147086681
H	-0.45412183792465	5.01809239639780	6.04178172842763

1^{-}

Pt	4.72423789269014	1.22671242216321	2.76839450535143
O	4.52345440251219	-0.48773446304732	1.76631948797055
O	2.84603227489897	0.87217709808077	3.26424928979412
N	4.90953978543833	2.91571778696744	3.75045808034769
N	6.60827499375338	1.68333570611806	2.33394146555738
C	2.37527493913151	-0.26972553732825	2.72318575915887
C	7.44501785959287	0.95430004269973	1.56056933798569
H	7.02682764468086	0.03640328524212	1.14915692364176
C	7.06460891540384	2.86607194998287	2.89468917381663
C	1.58552679189481	-2.67848916187991	1.49708410276540
C	8.74824915954667	1.35792449452046	1.31356524922395
H	9.38435815432538	0.73736323944271	0.68429240579610
C	8.37504753399003	3.31020258744813	2.67034180160112
H	8.70164651883008	4.24459469960432	3.12611979120746
C	2.88477950395663	-2.19925893093739	1.30580706165442
H	3.61234137318328	-2.73021493299112	0.69223673081617
C	0.08676069271408	0.06489705619578	3.82077595628597
C	1.05540781864767	-0.73048243266861	2.93686341111877
C	9.22558792849440	2.55451751753381	1.87551041172232
H	10.24737954740502	2.88384941635913	1.69014026184117
C	-0.11642289021985	-3.67590570538513	-0.05855157218557
H	0.17214339292979	-2.97735717990612	-0.85785409444518
H	-0.94398376344765	-3.22327368389342	0.50506844611457
H	-0.48823201415042	-4.60130200707417	-0.52520808948265
C	3.55183649968437	2.97195728316193	5.77678618865602
C	3.79424869911619	3.44411982721606	4.47739419535454
C	0.70358608937080	-1.93096504189153	2.30886818253258
H	-0.30655770517286	-2.31094024514958	2.45333940799085
C	2.90055644195540	4.30019217480699	3.81094768762016
C	1.08831566284498	-3.98209988001755	0.85751528653087
C	4.49264804615563	2.00170486326439	6.43544609268518
H	4.58255141012412	1.08424965955403	5.83256954839240
H	4.13283016722467	1.73053732545569	7.43542232742593
H	5.50658212077081	2.41756046904988	6.53313845366810
C	6.07453096419355	3.52875330536165	3.68308047020098
H	6.25065138588483	4.47260014174363	4.19868506086943
C	0.66453442626488	0.17483326600086	5.24977313836826
H	0.79422465486353	-0.82446342010326	5.69215294516141
H	-0.02390478357876	0.74865820241666	5.88944241233877
H	1.63279941507172	0.68873506935425	5.24158050990312
C	0.64740749564999	-4.96270973344944	1.96572172726311
H	0.28402900055960	-5.90283175314139	1.52237218449016
H	-0.16208158343164	-4.54154255263907	2.57787746290300
H	1.48991134449088	-5.19847289923412	2.63257645629879
C	-1.29182101440752	-0.60669196766989	3.91057432372337
H	-1.77163685116367	-0.68871397709967	2.92396558896995
H	-1.94764228889261	-0.00186223069272	4.55403127349760
H	-1.22855233526561	-1.61402476373940	4.34818566562127
C	-0.10555719188803	1.48428144535500	3.24028143009906
H	0.84444264111209	2.03063091157115	3.21223956131009
H	-0.81102538298254	2.05135906969392	3.86734334574003
H	-0.51756668619565	1.43262108300346	2.22105633879909
C	3.15519474416017	4.72911811261262	2.39205462679211
H	3.29342246348973	3.85122092473168	1.74158984159272
H	4.06916583507402	5.33580412853426	2.30540119183478
H	2.31436998997345	5.32035998233567	2.00953819041025
C	2.38506758349399	3.40242580166558	6.41868995786850
H	2.17813735085258	3.03745906019039	7.42748150548395
C	2.17354827520699	-4.66731998629805	0.01383479573628
H	2.51364267718017	-4.02404441320668	-0.81097429262262
H	1.76956746627676	-5.59118687710395	-0.42535161481798
H	3.04878280454837	-4.93818715639873	0.62243877095972
C	3.27668970924550	-1.00053534331556	1.91641766944010
C	1.46803498149638	4.25450141763955	5.79405605706989
C	1.74788904182690	4.69771610304928	4.49482508806345
H	1.03703698627313	5.35278740279584	3.98516631605386
C	0.18481023124436	4.64603028470585	6.47866066252814
H	-0.04398507864505	5.71065626694891	6.32754975481838
H	0.23086333939321	4.44942677598753	7.55787760255135
H	-0.66211749965186	4.07142861569682	6.07075071216488

2

Pt	0.98259656729771	0.60525721835271	-0.09057133387330
Cl	-3.64110162411476	-1.15096189444758	-0.15223043572844
Cl	-1.28730235833108	-5.12765794422400	2.75690513369306
Cl	-3.82686461607998	-3.94934584224220	1.32445176347898
Cl	1.45935494120157	-3.53569921678818	2.65029548033534
C	-3.06941847268246	3.38439127873665	-2.51825626128900
C	-1.04693246960825	-1.15272220721867	0.58177136383190
C	-2.26636207164962	-1.84425053415931	0.63677090936133
C	-1.20772740179104	1.81040308670346	-2.71825656467935
O	-0.90753789744362	-0.00026604255112	-0.07668313857444
N	0.78288557904072	2.11513960831632	-1.32520541252318
O	1.24327985367255	-0.99425537889256	1.06645150122123
N	2.85804191982870	1.22900246771967	-0.17335258499974
C	-0.49712100232348	2.57727718244277	-1.77598946569503
C	0.10786525993062	-1.68797670936030	1.20637405945552
C	1.89507358924788	2.73557594864482	-1.68308238647816
H	1.87665182896959	3.54589084506611	-2.41161253711928
C	-1.18959169586181	-3.60413290825148	1.94131359153557
C	4.37739169470842	2.80344399362250	-1.21282780374116
H	4.52591776989201	3.61772770642844	-1.92040279565724
C	-4.45615134926251	3.81176059902031	-2.91665145575592
H	-5.00388772219542	4.24130550088384	-2.06649523657655
H	-5.03929935045736	2.96486083203912	-3.30351944850688
H	-4.42431710848805	4.57909320645640	-3.70583862403276
C	5.17150745775088	1.23229049414653	0.42785971894699
H	5.96310910376141	0.80870081573257	1.04405578140988
C	-1.04671817564635	3.73143571862403	-1.18516673234244
C	3.08712092032618	2.27617524390645	-1.05092192848132
C	-2.33402749848787	4.11337047250624	-1.57878345855387
H	-2.78732775832610	4.98752362181452	-1.10481709860848
C	-0.63533501455472	0.54798181263377	-3.29867217565199
H	-1.30468532454645	0.15190056868612	-4.07213671488150
H	-0.54197712873033	-0.21598495662341	-2.51562112679145
H	0.35941373491546	0.69859503065024	-3.74282960732071
C	-2.33490484186473	-3.07402527965636	1.30585749452185
C	-2.48578558882996	2.24280341829483	-3.07830661667843
H	-3.05902460457606	1.63875799333749	-3.78217294489102
C	5.42650203175144	2.28615774971988	-0.46907855351750
H	6.43250572766882	2.69465143357982	-0.56694997887204
C	0.02933588090366	-2.90818665654099	1.89923320648019
C	3.88781975749635	0.72399221481995	0.54331127024090
H	3.62806435565936	-0.09485441957743	1.21235587439753
C	-0.32744788538341	4.50589771528945	-0.11336365044326
H	-0.55540706845233	5.57654672783929	-0.19190848294355
H	0.76008354161884	4.39438784056455	-0.17351158313562
H	-0.62282548595441	4.16640164395475	0.89122898943331

2^{+}

Pt	0.99298733639158	0.59858247703332	-0.10639449865437
Cl	-3.64807558686586	-1.15806196364113	-0.21697529855253
Cl	-1.32484359063666	-5.11879590467303	2.70767791283181
Cl	-3.81792332381266	-3.95987135580623	1.24351933755431
Cl	1.42490296641808	-3.54759944152726	2.67755809974042
C	-3.07762802405115	3.36286733522657	-2.47599887700517
C	-1.06338534150465	-1.16024561082207	0.54502296483492
C	-2.29598293844559	-1.85105428569699	0.58049705164178
C	-1.22539359190206	1.77980244770016	-2.68532240900601
O	-0.91520564423736	-0.02952811744789	-0.09065686422885
N	0.79321468889554	2.11561222737015	-1.35048798345945
O	1.21118073727028	-1.01127073150532	1.10013033552596
N	2.87531631942638	1.22722932230732	-0.17389524761816
C	-0.49282423379509	2.57535492720443	-1.78075303704211
C	0.10607308914210	-1.70381440543554	1.20285250160869
C	1.89659549971654	2.72937122095991	-1.68915541404021
H	1.88696122699488	3.54375885212434	-2.41471741985781
C	-1.19538533044330	-3.61468650751118	1.90875359745662
C	4.37460085648892	2.81509129334908	-1.19700800245230
H	4.52245876628375	3.63182175530544	-1.90149202502091
C	-4.46878842989115	3.78464641785694	-2.85389955421392
H	-5.00579014204162	4.21726717001554	-1.99922513789166
H	-5.05487217308360	2.93903339285513	-3.23724220072627
H	-4.44252290254110	4.55236319383423	-3.64254503018622
C	5.17780555264708	1.25921206013866	0.45291188349378
H	5.96823793750728	0.84334045501250	1.07586488546049
C	-1.01747371310992	3.75383965491592	-1.20828962392073
C	3.10032610398198	2.26968647394204	-1.04319149618337
C	-2.31635527186221	4.11792522333215	-1.57636705730979
H	-2.75588922599575	5.00866838764928	-1.12192637727951
C	-0.65140507762026	0.52122265040769	-3.27228629418402
H	-1.32765067245059	0.11538426549644	-4.03353476346246
H	-0.53490218350686	-0.24702197600273	-2.49563533325215
H	0.33270814363528	0.68026276181043	-3.73617556331255
C	-2.35683721509600	-3.07478495116294	1.24037550848906
C	-2.50874111458537	2.20573316110842	-3.02437218016946
H	-3.09343011123345	1.59868556469082	-3.71544585153395
C	5.42769112833471	2.30653639017884	-0.43755884052032
H	6.42922554691590	2.72706566431193	-0.52868777592628
C	0.02170848723907	-2.93159100430581	1.89962886344852
C	3.89204655390235	0.73228100356527	0.55553846896184
H	3.64164479379279	-0.08993500232524	1.22349034727600
C	-0.27677498617641	4.56828462585837	-0.18290675765985
H	-0.49595398682305	5.63558889688925	-0.30822282617962
H	0.81007713849220	4.45211319147029	-0.24195750009178
H	-0.56628806176497	4.28800879394251	0.84152548261760

2^{-}

Pt	0.99278998503115	0.57909525076522	-0.11445409943896
Cl	-3.61612092710630	-1.25492393327609	-0.22899696881297
Cl	-1.27649789622368	-5.17995173869754	2.77665199282072
Cl	-3.81378445489885	-4.03912996933691	1.28069068413908
Cl	1.45381916874937	-3.55343154277045	2.69795977981877
C	-3.08043598524134	3.42649348983736	-2.51002150707716
C	-1.03131029157676	-1.21051047193488	0.54195246210293
C	-2.23632640065022	-1.92484526184442	0.59260646274564
C	-1.26201727430849	1.80276272373277	-2.70159310903640
O	-0.91381029582801	-0.06458913497721	-0.12315692158755
N	0.77363142020011	2.08479454209824	-1.38585358780590
O	1.25511514985217	-1.02674714972863	1.10115517551115
N	2.86288173877404	1.23628529206908	-0.16011265870474
C	-0.49340347279461	2.56703244139089	-1.79738268461166
C	0.12613570336091	-1.72431746920062	1.20205518163844
C	1.91941349604180	2.73405281850685	-1.70258859405941
H	1.91592253372989	3.53017918256224	-2.44733790589589
C	-1.18529141912989	-3.65504520497728	1.93743376541698
C	4.39297698439327	2.82487615609282	-1.19266975019362
H	4.55289490649333	3.62990613588862	-1.91013517178312
C	-4.46598408541251	3.87836378561027	-2.89318914019573
H	-5.00296643367790	4.31003245795232	-2.03550086861996
H	-5.06485186166752	3.03906707699640	-3.27543667077009
H	-4.43846728862063	4.64975957640515	-3.68030834418217
C	5.16899826774411	1.28116873819899	0.49658434961593
H	5.95061621232173	0.87717276091286	1.13781248362563
C	-1.01581989234889	3.75381818764329	-1.23808478255131
C	3.08165659520175	2.29828045769815	-1.05585860481471
C	-2.29963714379518	4.16537376044970	-1.61583033850656
H	-2.71497475336873	5.06584879170420	-1.15264215750387
C	-0.73081786300522	0.51507520477914	-3.26129182426142
H	-1.40746042158154	0.12453695660590	-4.03376058099728
H	-0.65864074901340	-0.23230201671749	-2.45915906932574
H	0.27466002294958	0.63509503789827	-3.68915300124640
C	-2.31577024261228	-3.15022116637982	1.27924003732509
C	-2.54067425412920	2.25053120728865	-3.04118574432729
H	-3.14555827245635	1.63982893091682	-3.71384367720337
C	5.42545981966617	2.33246915179086	-0.42497077686252
H	6.43010612767035	2.75068837144502	-0.51468494987426
C	0.02831628029758	-2.93791964122149	1.90417758028075
C	3.88517167559833	0.76589009570874	0.58280185295022
H	3.62002176540754	-0.04823998968553	1.25690316152096
C	-0.25982392887613	4.52715983000871	-0.19119516112415
H	-0.47395233749757	5.60226400586463	-0.26994080243991
H	0.82319973893251	4.38785592708407	-0.27728379789691
H	-0.53394964659446	4.19479634484217	0.82261828219873

3

Pt	10.89162897505121	7.63891043332048	3.56140090042711
N	9.75621040495693	9.20986889129271	3.06581646334936
N	10.19898403341031	6.42900159526220	2.08945327860634
N	11.94110887749400	5.95270115285486	3.98719145963136
N	11.45249707409545	8.98855090016246	4.98193655751733
C	10.09364915776722	10.38834244132420	3.68594084277588
C	9.45740122584051	11.59052832801087	3.36901090387486
C	11.02957074118693	10.20771342689683	4.75658119140336
C	8.73246634152438	9.19914279332085	2.19047162174822
C	12.20770561869203	8.77543820198586	6.18288287987057
H	11.29198246356383	11.02668834231218	5.42789100246733
S	10.76794427612947	6.77480544759601	0.54389003178277
O	10.10274238572536	5.88119228875243	-0.40288444989287
O	10.63386885237223	8.21383724816427	0.33174356914087
C	12.49108560212561	6.36125128712800	0.57372437153578
C	8.42986049624356	11.58280463863939	2.42932984293733
H	9.76501951778568	12.50584905154269	3.87329654556623
C	10.36570319050431	5.05093719082307	2.45013733036048

3^{+}

Pt	10.96133586881544	7.67157064620627	3.53861038782735
N	9.73015903236856	9.20621739911957	3.06339570170079
N	10.39332471881374	6.48257409318036	2.04129047554214
N	11.98213108060815	5.98609937886674	3.99231722975593
N	11.37922481458751	9.00477128855050	5.03967177666817
C	9.88453563364309	10.33932605378210	3.81736959116193
C	9.11508241595907	11.48058979074280	3.59681185967676
C	10.81584601304962	10.17045320766556	4.90184680620812
C	8.77873353555368	9.17969606471178	2.11535820093972
C	12.23341929747847	8.83066554204539	6.18260780660354
H	10.97820046128334	10.96414009113779	5.63467109932419
S	10.88389982702966	6.88233794587443	0.41305371373632
O	10.12367847270171	6.02769781909237	-0.47703064273589
O	10.78965775483794	8.32762789284818	0.32177412630013
C	12.57024032679291	6.38687976233285	0.48433977636753
C	8.15195487643074	11.46176873250445	2.58829307222574
H	9.25716336181807	12.35143739801736	4.23645070691240
C	10.46082619370522	5.14223864224494	2.38762694010900

3^{-}

Pt	10.97644617914332	7.73705852262704	3.51039077713536
N	9.69626165736771	9.21628366354500	3.08547951017207
N	10.37760528161215	6.54659052273978	1.96541919751446
N	12.07395425381496	6.02438285633940	3.90263639583669
N	11.38138498907231	9.02193919661754	5.03578386809634
C	9.77794689078509	10.33432174616254	3.92356800270367
C	8.91475985932719	11.43871478308481	3.70908580566409
C	10.70331163448999	10.18675475477266	4.96278103836342
C	8.75413513556843	9.18456614677856	2.12405388018620
C	12.23676618176231	8.85027949875097	6.15536708217142
H	10.83453041574419	10.95751872100189	5.72416397181183
S	10.95416095424301	6.88100648284520	0.43171467926305
O	10.27372347011596	6.00340202351166	-0.51859630905466
O	10.88214324941852	8.32164970105252	0.22868680685923
C	12.66232813857487	6.39193100180051	0.49947206164283
C	7.97645168645885	11.39904113417872	2.70074701334320
H	8.99399399545448	12.28854045506768	4.38875340941849
C	10.48730385313407	5.17315345385908	2.33863723838278

S	13.52797156965580	5.91413552703352	4.44376178047246
O	14.08066410125170	7.25094907922922	4.28633716184095
O	14.21912555907718	4.80533939040805	3.78785237002373
C	13.51825855622922	5.51920787057215	6.18231768592722
C	8.05414730271118	10.36603580620199	1.85201042722286
H	7.91617907757654	12.50605841853603	2.16390443898382
C	11.33431045931125	4.79160752971484	3.44363419735352
C	12.30614914328838	7.90200697863780	8.41729199033973
C	13.57932809393378	8.44935599460717	8.59504066039655
C	11.60356238666090	8.03868019413303	7.21599491060336
H	11.84687921140385	7.33337711433557	9.22813515205406
C	10.22583238352744	7.45876054683733	7.06033964934599
C	13.48364936593949	9.35215612450833	6.32044323108040
H	8.47755121516988	8.23221290866630	1.76620354209825
H	7.23880254219745	10.31064069042444	1.13250357001786
C	14.15252666415215	9.15898692586371	7.53200569905040
C	14.31507011062692	8.28851130140173	9.89812946638268
C	14.12798647613106	10.13444491067005	5.20762284641938
H	15.15934657304753	9.56926935534291	7.64261390204134
H	10.14370539055764	6.86547907852324	6.13677795136950
H	9.46492465204551	8.25185160263260	6.99012939366548
H	9.97846914759974	6.81948174489686	7.91638632555841
H	15.38695645216157	8.11387585536928	9.73136253401125
H	13.91376622757276	7.44892398143940	10.48007038803332
H	14.22490302042180	9.19659409470666	10.51573879459259
H	13.91236594528476	9.68168989311489	4.23242048858197
H	15.21630617471083	10.15671935269040	5.34597466289147
H	13.77715240865072	11.17812821047097	5.18611333818627
C	12.88574284555504	5.04316457915384	0.32670869304248
C	13.42058638991823	7.33293096875286	0.95387732154971
C	9.63788428232151	4.00821004503375	1.87301600871660
C	9.89145005616840	2.68971286482303	2.26064578744871
H	8.89222372507790	4.23984963104361	1.11459053013338
C	10.86775387671351	2.42487987458426	3.22684186382856
H	9.32285149992275	1.87362111637658	1.81340852898497
C	11.58356336636577	3.46518745351810	3.82443146074222
H	11.06794404845456	1.39758370994851	3.53499293715170
H	12.34246648040260	3.24092878439100	4.57050071152775
C	14.22984642707316	4.70428513943244	0.46790654371787
H	12.14781625197368	4.29790048450825	0.03370880478807
C	12.41490186556073	4.91134289996268	6.78460576777195
C	14.69147453831331	5.74011099000845	6.90745034129075
C	16.61076509767743	5.26201152597721	1.10214473910854
C	15.18182966640430	5.65731399620685	0.85672266071908
H	16.89459450417232	4.38057319407660	0.51184047421595
H	16.74327593603090	5.00788701688318	2.16623096491413
H	17.30353292751979	6.08218597137221	0.86919834581535
C	14.75900636115293	6.97672659172799	1.08008903048016
H	13.08787676968619	8.34862953950665	1.16424423673668
H	15.48541464480604	7.72918889733273	1.39063494981970
H	14.54235860062672	3.67344356906281	0.29108266975672
C	14.75575708053932	5.33615313415306	8.24020626053565
C	13.66252126854264	4.72299071305317	8.86797247948437
H	15.66965617686976	5.51494826327985	8.80933862002465
C	12.49269780092759	4.51970740300490	8.11879253900507
C	13.72751645084433	4.31126447273419	10.31418464976968
H	11.62299903535104	4.05831152608416	8.59101840272758
H	11.49552062842490	4.76483338782349	6.22065719950691
H	13.55791537108824	3.22979273341540	10.42711225586292
H	12.94976477399483	4.82026083084118	10.90380891654865
H	14.70339674664692	4.55241270576996	10.75459327462445
H	15.54230505950664	6.23050184579919	6.43497112800188

S	13.61406575072531	5.90517366891021	4.52371227549444
O	14.16403665594971	7.23200577402944	4.33456992429439
O	14.24275100268186	4.76625751832966	3.87669899012434
C	13.48148166866660	5.52901456241261	6.24845614904162
C	7.98043606827588	10.29201016007638	1.85017465763765
H	7.52765865564000	12.33478086239083	2.40121898417658
C	11.38574345349702	4.85798050655937	3.45870204400067
C	12.53680385439660	8.02497520245591	8.41703479087832
C	13.80817268668863	8.60389795794687	8.46465038938494
C	11.72786143014847	8.11947042572243	7.28346173096912
H	12.15947446973435	7.48427770596817	9.28704030867358
C	10.33820083685139	7.54652529093252	7.27269018858273
C	13.50248696948401	9.43251419762545	6.18268190332725
H	8.66789188261638	8.25740358926416	1.55376424437883
H	7.23078425547326	10.22700620433212	1.06480256501886
C	14.27577730310578	9.29131641385083	7.33924889095473
C	14.63730022698143	8.50410186846178	9.71497646190347
C	14.03135335965190	10.19169076798049	4.99547298216081
H	15.27357595308762	9.73732127013354	7.36241051623471
H	10.12460827462690	6.99508876091971	6.34189558511747
H	9.58102673675913	8.34219691352690	7.34258007613066
H	10.20639280627518	6.85668396478236	8.11215631341802
H	15.70888821637300	8.43962940842797	9.49014617025587
H	14.34703707049417	7.62687471490899	10.30724083826315
H	14.49000910076922	9.39009859387886	10.35180033989700
H	13.58978969175629	9.85012374760584	4.05293443355426
H	15.11715482223116	10.06013456250084	4.92115547551085
H	13.84211459726660	11.27209924718617	5.09235212744856
C	12.89297935325220	5.03899232817010	0.27714424926198
C	13.53239656047663	7.30953242409693	0.90624456990568
C	9.78915846393276	4.09674393442586	1.73016775864504
C	10.02739972037336	2.79129046920093	2.12575602860404
H	9.10406139742054	4.33664531649549	0.92169526153606
C	10.95141883807142	2.50478789980104	3.15874079107998
H	9.48374601944680	1.97823051477706	1.64417178679939
C	11.63201095303342	3.51347213054600	3.81562365221386
H	11.11400725398640	1.46706597665446	3.45060815067945
H	12.34924141882624	3.27977781140517	4.59606677613925
C	14.19596146765181	4.62028304869914	0.51447388397873
H	12.12372204206924	4.34368889165345	-0.05471104705913
C	12.31501110728000	4.96995803948567	6.77628518428642
C	14.63332124334571	5.67023926471068	7.02815281487940
C	16.56739632118980	5.04467410693380	1.27206167645203
C	15.18405306566966	5.52249312186349	0.95152788932987
H	17.04582601780978	4.56305864817163	0.40614467544168
H	16.53486159297183	4.29822820428134	2.07962002439047
H	17.21042326236276	5.87121106528274	1.59764099703531
C	14.83474397755520	6.86950348844481	1.11884016838378
H	13.27109535469610	8.35591830828629	1.05572878190149
H	15.58986119435932	7.58568348160953	1.44355310936014
H	14.44062330603583	3.56545850548213	0.38252941166825
C	14.60677459291733	5.22641546839962	8.34793975573862
C	13.45656024507654	4.64131688475897	8.89848547647126
H	15.50067237644617	5.32042345617921	8.96603054975836
C	12.30891937306841	4.53382390515789	8.09690691071042
C	13.46241721681642	4.09901297567676	10.29889925426103
H	11.40517805827830	4.08973650178378	8.51764435812531
H	11.41713757036792	4.90941824687765	6.16646730075045
H	13.43905996810977	2.99972647915869	10.27572207346992
H	12.58329977930862	4.42202435759560	10.87554376845526
H	14.36568736000630	4.39455088449262	10.84871076833253
H	15.52921204210264	6.12075824939770	6.60355520385689

S	13.61346235130698	5.90663195083684	4.43378734999467
O	14.27368779649844	7.19873838576816	4.32549298928255
O	14.28313642661836	4.73457917962615	3.86724381847711
C	13.48867964030228	5.52501725924506	6.18160069342357
C	7.88420941398911	10.23848302762965	1.89272504769773
H	7.29418362940076	12.23642085886597	2.54911914050099
C	11.42342303816844	4.88224357384691	3.36014968884626
C	12.58447344137656	8.04906780901311	8.40331086268218
C	13.86939618425114	8.59700259474053	8.41989177966173
C	11.75387892032955	8.16675556287921	7.28625025614761
H	12.22260660729725	7.50049385313059	9.27702361592394
C	10.36121171314650	7.60175589628827	7.29077742478321
C	13.52961376629557	9.40492604534581	6.13868337727768
H	8.72350311729942	8.28255924781914	1.51937933261869
H	7.15216761592039	10.16111695453752	1.09268614851259
C	14.32745334391253	9.26452626101377	7.28010553146037
C	14.72470046927177	8.47093559643676	9.65267264888353
C	14.03418933678009	10.12297622853278	4.91746828516623
H	15.34124119927917	9.67777267329963	7.27090697000283
H	10.14611570605167	7.06937969084911	6.34988136762158
H	9.61068946354061	8.40412732628915	7.35879065489073
H	10.23309480270082	6.90560519424578	8.12815502187332
H	15.79276555797991	8.39495859907846	9.40662687643423
H	14.44055883801650	7.58023112364185	10.23048576930193
H	14.60724754549084	9.34198798231975	10.31929831030443
H	13.54819596281429	9.72944477495848	4.01876152118255
H	15.11908174233759	9.98915371885191	4.81848185604824
H	13.82887215939312	11.20445933003564	4.97474507483929
C	12.98453344365111	5.03941896276125	0.33998725986651
C	13.63340748622955	7.31573701905057	0.88757035504111
C	9.72831342157391	4.15224417091270	1.76166939522643
C	9.90411922975864	2.82958904080557	2.17815179552303
H	9.03618164475023	4.41469804353091	0.96419786695048
C	10.83976867192427	2.53428416374772	3.17409920290166
H	9.29597894452614	2.03959367514728	1.73349111311114
C	11.59579697305546	3.54977154908184	3.76526410884135
H	10.97837589027672	1.50118501630967	3.50006842083719
H	12.34369260771415	3.30401475429074	4.51379412135151
C	14.28443478147954	4.61585702649419	0.59766047423285
H	12.19934483578371	4.33540138151100	0.07043688700906
C	12.30738857329343	5.00496335321248	6.71142823937655
C	14.63094897507254	5.64320673913113	6.97617873068817
C	16.63227934366054	5.02846255014517	1.42072808165643
C	15.27426806030862	5.52209619153864	1.00331692634075
H	17.07961332180385	4.35362680444370	0.67385259754598
H	16.53504093715083	4.46759784596171	2.36337792237684
H	17.32634672826899	5.86159715418830	1.58950995334677
C	14.93419466131119	6.87572448149972	1.12047153323670
H	13.36890365469615	8.36316143216963	1.02626525649498
H	15.68730114762837	7.59458549579125	1.44810063395322
H	14.50633441307675	3.54812387863413	0.55408978001671
C	14.58844908791295	5.20888816514012	8.29990060385350
C	13.42151157849152	4.65486127593545	8.84365584141107
H	15.47617398922162	5.29390894054915	8.93060743242311
C	12.27956407722179	4.57046712966825	8.03456073791724
C	13.40767615737456	4.11733625167253	10.24994550354198
H	11.36047985314127	4.15244008874091	8.45211480664185
H	11.42143895947878	4.96351875567846	6.08155110430482
H	13.37797802915613	3.01699739181002	10.23897873199519
H	12.52898033005909	4.45451565228542	10.82152161715666
H	14.30979617044167	4.41255111997140	10.80553883695291
H	15.53286640464888	6.08197913432739	6.55172828452600

4

Pt	2.60809865997126	4.54298686668800	3.90486727040655
N	1.99484702627629	4.90711225900843	5.78074161008211
N	3.22509750048428	4.25486413697316	1.99240723958827
N	4.34122742608641	5.60207730268114	3.95756044088376
N	0.83512656812073	3.57869098958994	3.91468199089536
C	2.54869512991083	5.77465104419707	6.64763020439799
C	0.89996650784822	4.17302676976914	6.17410397067626
C	0.40019916256122	4.24776153946052	7.47554741029651
C	0.27812983789314	3.46056574038969	5.09312966770025
C	0.15999067801192	3.04741036490922	2.77006637776696
H	-0.66840778513519	2.93411226230037	5.22288240161387
C	4.66885243061790	6.04946028933387	2.63263949184690
S	5.61112142153587	4.93651732011843	4.81039087633416
O	6.74810957361239	5.85669546558637	4.76458417607966
O	5.10866344741457	4.55131645467901	6.12853019281071
C	6.08814938110587	3.44694218983425	3.94490825495239
C	1.00427780970756	5.11572270199894	8.38227504494214
H	-0.45960353006254	3.63909800411945	7.75297376116854
C	2.07844903206164	5.89946921968268	7.95237665934321
H	2.56453770698790	6.60743918288744	8.62172275587849
H	3.38653773351413	6.35521335555394	6.27284321131454
C	-0.31507917606819	3.95595236226191	1.80159726790281
C	-0.01841450990282	1.65785426816099	2.64337205229766
C	-0.68713113257126	1.19100583989758	1.50429789345351
C	0.50250058060939	0.68708571378723	3.66857624100858
C	-1.18839660555905	2.05634210527932	0.52614114533117
H	-0.80752630372685	0.11269585846004	1.37736301891416
C	-0.99392183586768	3.43430558336674	0.69657118734323
C	-1.91919647296827	1.52707244178327	-0.67924774594181
H	-1.37255874564621	4.12860270512643	-0.05719314159260
C	-0.13099718742227	5.43968680539652	1.95629290128445
H	0.61468439435084	-0.30821565516914	3.22092870080456
H	-0.18803644796969	0.58871758512622	4.52038618492688
H	1.47825466079485	1.00500024266612	4.05515750869909
H	-1.57872567387837	2.02189199947213	-1.60003834091521
H	-3.00144157211704	1.71295847298519	-0.59343243986443
H	-1.77273422144705	0.44499555783727	-0.79086184844448
H	0.93742852232924	5.70378157678748	1.97101726428825
H	-0.55827961052188	5.80183942668792	2.90308451656199
H	-0.61468415166813	5.97457867984282	1.12993985013126
C	5.48484702565509	7.14922917291848	2.35640547734524
C	4.07218493784984	5.32352523762117	1.58049020268155
C	4.30643930249844	5.70110031758420	0.25508414557700
C	5.13730540084538	6.79194080416515	-0.01762248691369
H	3.84347822567822	5.14870915928363	-0.56200624788884
C	5.72268542033462	7.51492205706341	1.02728623670089
H	5.31349457087571	7.08655644595221	-1.05299469133721
H	6.35595444395649	8.37590491771494	0.81011196068985
H	5.93235668973052	7.70193391946972	3.18060757042033
O	4.89592874945003	2.69007736057837	0.83260874795013
S	3.53973015186849	2.74158029532967	1.38476146865895
C	2.41970867650802	2.52311324628767	0.01612328629483
H	2.62961598432078	3.29075815709013	-0.73633979530254
H	1.39400587301804	2.60996993865700	0.38425198251572
H	2.60789418606955	1.52319507038199	-0.39291367605974
O	3.17621550489402	1.76607170404015	2.41008358806583
H	5.25686724905067	2.73586505607483	4.00055135583309
H	6.98302361965707	3.05540871116180	4.44300827783683
H	6.30288497492096	3.70281620856007	2.90098484977785
H	0.63375878354412	5.19295719054826	9.40378252198625

4^+

Pt	2.57584661782693	4.52898225411660	3.87036568345834
N	1.97877466293178	4.86893885651580	5.78203830297405
N	3.14013622912981	4.27614864034863	1.95858565677757
N	4.30795605148129	5.51826109614463	3.87630340382104
N	0.77553178934841	3.58730565588701	3.88760906931029
C	2.55435918654953	5.69714014768517	6.66883666722188
C	0.88371733380308	4.13617385921942	6.16426343484096
C	0.40053695310697	4.17258626977330	7.47139635625672
C	0.24229299642983	3.45270012777259	5.06971734727731
C	0.09733035325619	3.05561881230610	2.74505708998863
H	-0.70424196233628	2.92462441133493	5.20042920692408
C	4.64331491075790	5.99369182726576	2.62104724237739
S	5.59539523297251	5.10479850589546	4.95295259109597
O	6.36836577544362	6.30220475462122	5.21708817322564
O	4.97146561480140	4.37681217517450	6.04201156880418
C	6.55649218070341	3.95896907146288	3.97579060742759
C	1.02961983218369	4.99978408119062	8.40210562362946
H	-0.46342413306291	3.56622366758701	7.74143588242811
C	2.10329137503814	5.78508316261278	7.98575544527332
H	2.60774741907287	6.46477281470180	8.67039248682611
H	3.39043477492493	6.29030416351569	6.31171803708909
C	-0.37820260591819	3.97083802898729	1.78118141037381
C	-0.09239413689725	1.66560066257762	2.62441633924129
C	-0.77154468702282	1.20723338364843	1.48912632249893
C	0.42342202321304	0.68400268879224	3.64232914187132
C	-1.27661498122530	2.07572732819631	0.51551304342488
H	-0.90470355130059	0.13062660603626	1.36456345164793
C	-1.06910407712341	3.45288986562403	0.68352145577531
C	-2.03507151051256	1.55353221310031	-0.67341281721389
H	-1.46146921791401	4.14922062031951	-0.06081014320428
C	-0.21253570624836	5.45544995577081	1.95088193868379
H	0.60289669485027	-0.28857427326592	3.16897063981172
H	-0.30759030930142	0.51854765148447	4.44925114210510
H	1.36609490051595	1.01691990711630	4.09127116898595
H	-1.74384444135174	2.07418626488039	-1.59613973883378
H	-3.11662192008197	1.71262805273546	-0.54181869592536
H	-1.87270737148245	0.47783489229139	-0.81437953820747
H	0.84634904087493	5.73764708382499	2.04901308605562
H	-0.71891396552214	5.81356159171330	2.85985034522675
H	-0.63768998686894	5.99101468762912	1.09419810339052
C	5.60473987799164	6.98309276730116	2.33626209385794
C	3.98903798255765	5.28815928400335	1.54299260114476
C	4.32898254071573	5.58968185521507	0.21285265970100
C	5.26582081454165	6.57642563967356	-0.04358199213445
H	3.84464754250323	5.06130534304006	-0.60583082441734
C	5.89993471292306	7.27003829041831	1.01380325156735
H	5.50966393079842	6.83171377601122	-1.07458905854735
H	6.62049729497477	8.05458337300958	0.78336011813730
H	6.08594249721430	7.51122770971368	3.15676003326663
O	4.81578415451901	2.65640563562487	0.86128763494759
S	3.45247927455571	2.67836191472601	1.35768817002524
C	2.33877055705618	2.47148598259183	-0.01221526873747
H	2.60915621396758	3.18126027053380	-0.80075074433308
H	1.31297261876767	2.62727671465404	0.33419084710953
H	2.48338824683680	1.44176875305628	-0.36210596742309
O	3.04639261129550	1.79594965292567	2.43545242812083
H	5.91215835550256	3.12183168750425	3.68570148167604
H	7.36902247607602	3.62047124904255	4.63050947273147
H	6.95673019223764	4.47937204180423	3.09943678696047
H	0.67537071991877	5.04249649255621	9.43142974361072

4^-

Pt	2.62944617587929	4.50087248704530	3.94174924422139
N	2.00864687270365	4.86870218720826	5.81461884473508
N	3.23914655177896	4.23796112207459	1.99949515266400
N	4.38657419422422	5.56587750395908	3.98887010203056
N	0.83318356060730	3.58151579764906	3.89848064943264
C	2.61165488456176	5.66794707714929	6.71457545868999
C	0.80846598759314	4.23080384394072	6.15586842257305
C	0.28855397279336	4.37545076671803	7.46706883456788
C	0.20343026919202	3.54635473377868	5.09440598626306
C	0.17587374095343	3.05200434716680	2.76578894218763
H	-0.77369292200718	3.07039709032943	5.18698450101241
C	4.65874589145357	6.04815295109341	2.66954447776087
S	5.69309624798133	4.90716481249834	4.77944746974753
O	6.85997896662406	5.77201919739150	4.60235874795376
O	5.28133006209752	4.58147885431609	6.14131941150565
C	6.05410823642515	3.35862898606616	3.94554205703463
C	0.93331763108568	5.17995147813601	8.38245046997563
H	-0.63152328433125	3.84781212513316	7.72350771404536
C	2.11547890639520	5.85765714596213	7.99631584406463
H	2.65481325159059	6.50488173188153	8.68562202182800
H	3.53303209755173	6.13444237555374	6.37570295920395
C	-0.29034053082872	3.93265993568599	1.76343666494285
C	-0.01871241636372	1.65972803391209	2.64637283463222
C	-0.70215800204118	1.17257544899238	1.52537552440125
C	0.54770196632625	0.72140520501257	3.67491800421251
C	-1.19447777424693	2.02081519911929	0.52856917593014
H	-0.83294860859776	0.09160053124796	1.42102654545055
C	-0.97348161658299	3.39723241905550	0.66686580580685
C	-1.94094235343303	1.47392998022586	-0.66215281523066
H	-1.33830752450743	4.08027743909597	-0.10586443144255
C	-0.05443645629278	5.41222496100617	1.87281600046774
H	0.50300808260096	-0.31560061634570	3.31580352715677
H	0.00432912498067	0.77907592385240	4.63055415988172
H	1.59490757692519	0.98465987419458	3.87574223614875
H	-1.59041366526841	1.92867394998214	-1.60095513031128
H	-3.02211210229408	1.67727189924578	-0.58808145788095
H	-1.81378031669257	0.38548361331624	-0.74425784976900
H	1.01790196892061	5.63907419906293	1.77274983688453
H	-0.35503680495787	5.79270647437732	2.85913317269030
H	-0.60896589966058	5.95305549990204	1.09357155125521
C	5.44503418874909	7.17137824354761	2.39760917624599
C	4.05012868100964	5.34084950519259	1.60784469710327
C	4.23912210398224	5.76972069819478	0.29068996872643
C	5.03478117772261	6.88820978173665	0.02560928607796
H	3.77559690310631	5.21930729535008	-0.52807924413815
C	5.63496695238375	7.58784441788890	1.07614021273905
H	5.17597219658487	7.21706611239280	-1.00581511243126
H	6.24951795621343	8.46613564273792	0.86994459827715
H	5.91823450707701	7.69003617169273	3.22941448908572
O	4.92391918306855	2.75481170695241	0.74877167557715
S	3.59259714463727	2.75935239767972	1.35863809867179
C	2.43751434609232	2.53246160307272	0.00451002429834
H	2.58578012419399	3.33814836059304	-0.72319704349428
H	1.41824526858568	2.54921983133338	0.39867464307355
H	2.66668104872460	1.55963897753918	-0.44756690961901
O	3.26697047794247	1.73028647874971	2.34204018746643
H	5.19259100395164	2.69286589928908	4.06664768633159
H	6.95397837954734	2.94230760791464	4.41486483125099
H	6.22552478114015	3.56067684917290	2.88156699435306
H	0.53363763214791	5.29567583297627	9.39143107367959

5

Pt	7.72069471622800	7.74252026574727	0.52740147381944
O	7.94167493910283	5.88953248955801	1.09413933511264
N	8.52055861315702	7.48472270799986	-1.29601913143515
N	7.45916963971756	9.58375586801137	-0.27224430341542
N	6.99363105278045	7.93815806047586	2.38606112184635
C	6.76456164856278	10.58977413966276	0.25275263338865
C	8.02109635909088	9.74157834428901	-1.49206421052837
S	7.74892715159922	8.98473080082255	3.41575760026145
O	8.25013386112906	10.10299857336045	2.62229074200404
O	6.89385436081166	9.28422971173700	4.55153070849531
C	9.17496568832736	8.10586109155276	4.05339679838596
C	7.26871594746893	5.58468893047783	2.20579602879377
C	8.62298540803277	8.53655144678243	-2.01906204665738
H	9.10229146350928	8.51856014261842	-2.99328525253958

5^+

Pt	7.79396827938161	7.73628668766127	0.50734562887508
O	7.89485635349920	5.83400014520565	1.06062975899903
N	8.57683533747190	7.45841165463294	-1.31387018672592
N	7.54196721569519	9.58004000359585	-0.29969436776930
N	7.18705397301793	7.85704667016977	2.43175949636988
C	6.82886808558213	10.58582252568993	0.19342928383443
C	8.12248217671523	9.72472286858842	-1.51407528800569
S	7.48905995249852	9.16907756350438	3.43645632261721
O	8.23125904130774	10.11091062477795	2.61840338360338
O	6.26194166585836	9.58696093081993	4.08187845923839
C	8.61423225043934	8.53792507217722	4.66927782406234
C	7.27134344760568	5.53895629941406	2.14358887759360
C	8.71258568626484	8.50592182075887	-2.03064378017766
H	9.20833549293890	8.47594978335160	-2.99617977654988

5^-

Pt	7.83354105072651	7.80160185824521	0.59951268460772
O	8.15927980036264	5.94924267661947	1.25116801840003
N	8.55987998804786	7.48169901303988	-1.24727546197353
N	7.44740516401844	9.59441882956951	-0.24138720696371
N	7.08583054742001	8.00184611647185	2.50031304061706
C	6.78674156396016	10.61237160943665	0.30706844965853
C	7.87562038353863	9.68906230964592	-1.54905384583227
S	7.92507183240431	8.93940774848392	3.54812686645749
O	8.25114573455580	10.20101818801903	2.89310339248354
O	7.25390468956572	9.00850124053067	4.84220407189804
C	9.48318005456532	8.09256837692131	3.85133410005016
C	7.42378999907844	5.64030344323759	2.30313566749223
C	8.46340719634561	8.53274640512493	-2.06234359021575
H	8.82701711608968	8.48028315299044	-3.08302652441540

C	6.71758193286951	6.64866335811345	2.93303977209759
C	9.02263283513928	6.22941152373417	-1.74506594666112
C	5.97590764140914	6.42108560847568	4.07752539140503
C	7.10532740326277	4.26590446670946	2.64927584813001
C	7.95086586722093	10.93564070462829	-2.18769581433306
C	6.64796204201398	11.80806668195320	-0.39599370555360
C	7.25864667300189	11.99406276697952	-1.62445428630928
H	6.08364146595282	12.59931740708875	0.07525523682279
H	6.30112765072587	10.41490383369885	1.20913314163193
C	6.73760409209675	5.75810333418702	-2.69858496813322
C	8.16010967073461	5.37357135077925	-2.42154646997847
H	6.21156017571267	4.95127170453697	-3.20545788902964
H	6.65959330323033	6.64494200865026	-3.32972452618894
H	6.20109210346050	5.97262690539814	-1.77221263723905
H	9.83385455709251	7.83904517136981	3.23087552170435
H	8.85220540240999	7.21056717222404	4.57938160368737
H	9.68795498289002	8.77467145281089	4.74224587738848
C	5.79959413544666	5.12264136053035	4.54828347382256
C	6.37269435388448	4.08314623272071	3.82332913149633
C	4.99421666066368	4.89834731304924	5.82497309981634
C	7.68644436840738	3.08545710773513	1.87020999259450
H	8.42329534162667	11.02290362368343	-3.15654608644170
H	6.23573823816638	3.07492837365421	4.18202754648936
C	10.32665635086983	5.86640515976732	-1.41131466522877
C	10.76380470450579	4.61017710948520	-1.80422475766951
C	11.21639126771445	6.77941362579489	-0.62197308276377
H	12.20042415632468	6.33466647537802	-0.48521189958180
H	10.79951011442277	6.97426628448053	0.36772985062827
H	11.36052141291652	7.74662670699496	-1.10562668710350
H	5.53954952815273	7.26262510214110	4.59629872957469
C	8.64910845393760	4.12731216514898	-2.79515848944643
C	9.94177039397268	3.72565108980389	-2.49318508901430
H	7.98791655002484	3.44524588881652	-3.31695759114355
C	7.41642244428589	1.75075480694750	2.57069596243114
C	9.20976457735379	3.22298303886203	1.72936618751021
C	7.04140618190226	3.01079069113934	0.47874593419041
C	4.89194652426333	3.41970226100346	6.19936105185400
C	5.66956514843365	5.63824940076790	6.98757333607343
C	3.57105292332914	5.43977560255065	5.63700275548520
H	7.43272478407963	2.15319851629320	-0.07510202683797
H	7.24347053460396	3.90209944099716	-0.11005254628998
H	5.95962144441551	2.89025691277610	0.56028896112385
H	7.18655164438153	12.94105496621739	-2.14079394367626
H	11.77309950268383	4.30779377546214	-1.54812436780296
H	2.97759842054391	5.29125111955204	6.54227578876083
H	3.06531113436678	4.93073265837366	4.81515483893777
H	3.56695103495884	6.50712889912301	5.41482519162574
C	10.43911214877246	2.36017808506609	-2.86535245504629
H	5.10367059361462	5.50581728304495	7.91286464052536
H	5.74804218824907	6.70949861824378	6.79974861114858
H	6.67928551397078	5.25971239974137	7.15515237902057
H	9.61821900232394	2.35060039281670	1.21224094062416
H	9.68597247441773	3.27938874035375	2.71007541191074
H	9.49125885605362	4.10577321757688	1.16085892229081
H	7.85472746918767	0.93948790332056	1.98672817914897
H	6.34983144471319	1.53948210109696	2.66511646782600
H	7.86060730140078	1.70964518447665	3.56695778327253
H	11.37629401538011	2.41202410990880	-3.42064606181350
H	9.71885972036319	1.82338450569427	-3.48086564157420
H	10.62334273237838	1.75737204454349	-1.97464968733052
H	4.31199730904700	3.30907661118319	7.11733077038396
H	5.87184851812741	2.97402506375539	6.37934287397122
H	4.39073973261946	2.83469000549379	5.42612861925460

C	6.82767835575136	6.64988497806231	2.95548412643018
C	9.08825180503366	6.19412446584505	-1.73687535869728
C	6.15864388445915	6.41404991311460	4.15287042290461
C	7.05689618170291	4.19187653396962	2.56525887381128
C	8.06959453621762	10.90752064146283	-2.22107975867306
C	6.72065922835224	11.79892689031083	-0.47393832915036
C	7.36431845937370	11.97538591668808	-1.68196194750004
H	6.13474663918347	12.58892368044916	-0.02764815310607
H	6.32873455304633	10.43408655715113	1.13442994031877
C	6.78512149259658	5.66035809748688	-2.62128056460888
C	8.21568771576151	5.30085403729677	-2.35039253882137
H	6.24672178061962	4.81241770955781	-3.03953058021735
H	6.69547014947418	6.48149125397072	-3.33486753475929
H	6.26057425309379	5.96445604903337	-1.71313061982980
H	9.50710435492968	8.15915278425154	4.17870508671416
H	8.15042266959835	7.76336493690376	5.27356659158924
H	8.87385016483493	9.38545388609174	5.30234841146843
C	5.94860915088656	5.11452457227963	4.57393536590916
C	6.41483379528349	4.03940111468715	3.76673058555995
C	5.19596721699850	4.86873064399256	5.87434464632040
C	7.48955334095824	3.00919583245808	1.70480717196233
H	8.55864830507782	10.98516044223058	-3.18234648063642
H	6.23286977797110	3.03881676794012	4.12333751699246
C	10.41806680964704	5.87942910054237	-1.45593380790417
C	10.86677731517361	4.62093981043168	-1.82794677020050
C	11.33211853257590	6.85083805430067	-0.76835584545964
H	12.29868301737693	6.39307968745231	-0.56878482330448
H	10.92675389275512	7.18802314504863	0.18767043584172
H	11.52076477005810	7.74140503336293	-1.37055174388800
H	5.77300370183998	7.24639038247195	4.72448595925013
C	8.71969562000226	4.05547888948153	-2.70544385211059
C	10.03583835007270	3.69505754322068	-2.45106280030953
H	8.05822638716758	3.34652178737828	-3.18919785965702
C	7.19462985470850	1.67988539362953	2.40405813305403
C	8.99993916542197	3.05857691740750	1.42328624166695
C	6.70521334624095	3.02492585224364	0.38412039987642
C	5.09975434842667	3.38547236155904	6.23324067095868
C	5.92068419766666	5.59355292352063	7.01842830407402
C	3.77196308148490	5.42737264936287	5.73036831049527
H	6.97968446129900	2.15596757146124	-0.21646380207139
H	6.91560669448341	3.90883664946820	-0.21363648706341
H	5.63021927699935	2.97610639874785	0.56408629124846
H	7.30539981449666	12.91714716752060	-2.20928110548226
H	11.89645598079330	4.35354300124113	-1.61970496127969
H	3.21106290841477	5.25977064254606	6.65092193144124
H	3.23345065432123	4.93704651084712	4.91860332722784
H	3.76557920134532	6.49973236414118	5.53470176665904
C	10.55513767527137	2.33752018080756	-2.81838623762457
H	5.40056961040974	5.41629358101760	7.96060189038022
H	5.95558376419270	6.67350122138089	6.87145239293127
H	6.94420449129841	5.23348412174533	7.13292570166500
H	9.29776694712186	2.15224366025461	0.89312616072807
H	9.57287014194502	3.09620280466087	2.35121979062580
H	9.28940445888830	3.90377469449383	0.80367549909344
H	7.52164757504137	0.85889782810279	1.76607974320213
H	6.12962756455494	1.53104493992937	2.59000242364908
H	7.72819542474021	1.58118748553641	3.35107077614876
H	11.45865147301658	2.40811158276893	-3.42422381658142
H	9.82254058778353	1.76356335481231	-3.38283730700462
H	10.80958308268800	1.76245117279161	-1.92675904519152
H	4.56944004024932	3.27545625802280	7.17904923733094
H	6.08047178336573	2.92402252567847	6.36130336499885
H	4.54284722717963	2.81221439502686	5.49036900263944

C	6.80219918995961	6.69488407788666	3.00091418735578
C	8.97805557541384	6.22863029414660	-1.71935078059145
C	5.97587642448836	6.44054914989885	4.07732156875170
C	7.24268071592415	4.31597029090645	2.73822671512202
C	7.66960806328040	10.88768214733384	-2.26934623023697
C	6.53784603365112	11.79075593105741	-0.36434918813439
C	7.00756765922834	11.92832720657563	-1.68221409790018
H	6.00369727640172	12.58551240018860	0.13452976479845
H	6.46185448343513	10.47030882582160	1.32518695353165
C	6.76830078091509	5.98800467854129	-2.90371719502199
C	8.12994523134158	5.47501164107301	-2.53841001781947
H	6.18025278631976	5.21165903770017	-3.39315403781582
H	6.81067619434982	6.84609905170925	-3.57622232440228
H	6.22819736281600	6.31575690909064	-2.01388994479168
H	10.05021938849610	8.03640909876250	2.92577158385080
H	9.28313337593277	7.09042150368509	4.22546262197073
H	10.03428966861874	8.66274600795648	4.59763388511157
C	5.77244265948415	5.13647202944916	4.52634534397653
C	6.41859416472771	4.11041676585761	3.84692480668319
C	4.87747584458012	4.89891485135489	5.73886798117602
C	7.92015973068000	3.15345150463140	2.01122069668908
H	8.03258631488008	10.95434175811225	-3.28694521741169
H	6.26879157793067	3.09632715274870	4.18649673032168
C	10.21324717729687	5.71190134901107	-1.31523738564120
C	10.58403939678333	4.44684158192411	-1.75256650399303
C	11.11433693520085	6.49629561792002	-0.41091590130805
H	12.11457376561311	6.06247359638717	-0.38699689035394
H	10.72412775586508	6.50118138720620	0.60794292770084
H	11.19850926435148	7.53720677268583	-0.72140733016708
H	5.49888313771055	7.27773765302309	4.56739082900398
C	8.54394949248922	4.21641590794993	-2.95754514006004
C	9.76402279295543	3.68075487417087	-2.57005959066599
H	7.87868444796939	3.62799534929884	-3.58135038020121
C	7.60386857066240	1.80511457398833	2.66556660346348
C	9.44653004330350	3.32281112800827	2.03814534518257
C	7.43800951835183	3.07941800049968	0.55492504009478
C	4.73460043196380	3.41542732098501	6.08052153094199
C	5.47575863224939	5.61242020624064	6.95918024040967
C	3.47377924300478	5.45575375852333	5.46641737506641
H	7.90104591436921	2.23015459589854	0.04312811289988
H	7.69012286812082	3.97777545387684	-0.00228741355623
H	6.35512181717940	2.94427870123576	0.51480309003747
H	6.84115095333397	12.84757143843368	-2.22973125877695
H	11.54095027806048	4.04537377058573	-1.43467919006481
H	2.81691472223846	5.29962522281608	6.32697785462824
H	3.02318043323164	4.96524459575707	4.60207150027519
H	3.49678689823788	6.52574837103317	5.25968694877860
C	10.15999346819152	2.28929029411838	-2.97225879152496
H	4.83944148171170	5.48600031220521	7.84012196627790
H	5.59747266972909	6.68135425105683	6.78271056825598
H	6.46267476314714	5.21034041005525	7.19428374761435
H	9.93149711221507	2.47835824457727	1.53967667216247
H	9.81293540176515	3.36045172399673	3.06641212241026
H	9.75640439928088	4.23214119991635	1.52956323138818
H	8.11422968991552	1.00805222036105	2.12038730068076
H	6.53589175651401	1.57838341183804	2.64770933024917
H	7.94235447084396	1.75941976948258	3.70288691057897
H	11.24145957213242	2.18979375436604	-3.07165114505261
H	9.71160292956127	2.00035983894213	-3.92333995457719
H	9.83543940741152	1.56001206927575	-2.22614168125635
H	4.08460418400220	3.29325250377623	6.94999588624603
H	5.69580693416580	2.95962056029399	6.32487897668823
H	4.29359164533544	2.84918492545444	5.25795097868681
